# Galectin‐9^high^ Neutrophils Exacerbate Radiation‐Induced Frailty

**DOI:** 10.1111/acel.70448

**Published:** 2026-04-07

**Authors:** Zhuo Cheng, Le Ma, Yan Chen, Jialun Li, Yali Dai, Wentao Xie, Yingjie Li, Tianlang Xu, Zhihe Hu, Weilin Huang, Jingyuan Zhou, Jie Wu, Long Chen, Chunmeng Shi

**Affiliations:** ^1^ Institute of Rocket Force Medicine, State Key Laboratory of Trauma and Chemical Poisoning, Army Medical University Chongqing China

## Abstract

Local radiation injury‐induced frailty seriously impacts the quality of life of patients undergoing radiotherapy or nuclear accident casualties and causes a significant medical and economic burden. However, the underlying mechanisms of the frailty remain unknown. In this study, a unique population of hyperactive GAL‐9^high^ neutrophils is identified with characteristics of elevated ROS, NETs, and IFN‐γ, prolonged lifespan, etc. These neutrophils infiltrate into multiple organs to induce injuries, also disrupt the bone marrow microenvironment, drive sustained bone marrow myeloid‐biased differentiation, and resist clearance by bone marrow macrophages, serving as a crucial factor to exacerbate frailty. GAL‐9 protein is demonstrated to play a vital role in the regulation of neutrophil hyperactivity. EccDNA shedding after skin radiation injury is shown to activate the JAK1/2‐STAT1 pathway in splenic GMP cells, which is a potential origin of GAL‐9^high^ neutrophils. In summary, our results highlight the significance of the previously unrecognized hyperactive GAL‐9^high^ neutrophils to exacerbate frailty through a ‘skin‐spleen‐bone marrow‐multiple organs’ axis after local radiation injury.

## Graphical abstract

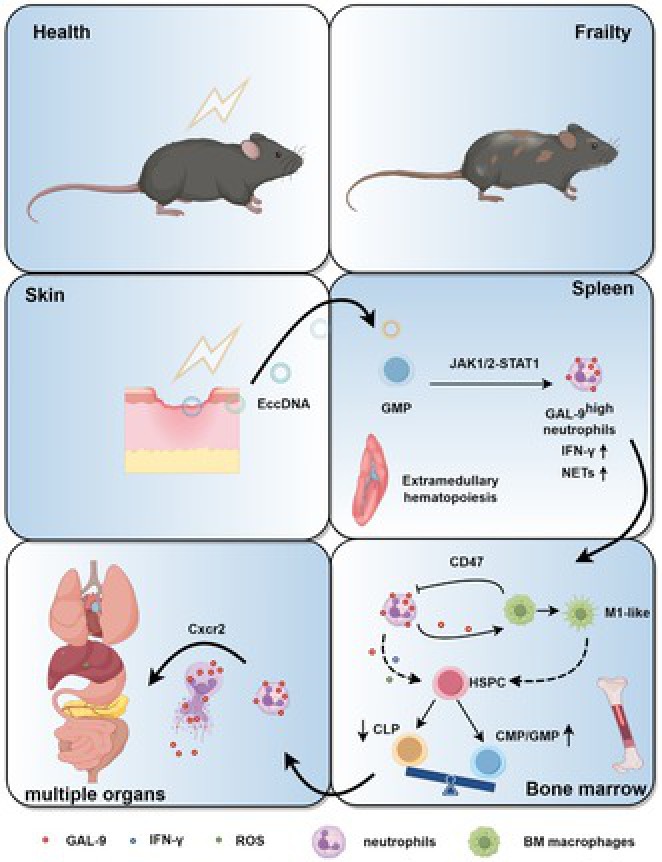

**Abbreviations**
ALP: alkaline phosphatase
ALT: alanine aminotransferase
ANOVA: analysis of variance
AST: aspartate aminotransferase
CLP: common lymphoid progenitor
CMP: common myeloid progenitor
CXCR2/4/6: C‐X‐C motif chemokine receptor 2/4/6
DSBs: DNA double‐strand breaks
eccDNA: extrachromosomal circular DNA
EMH: extramedullary hematopoiesis
ESCA: esophageal carcinoma
FACS: fluorescence‐activated cell sorting
FI: frailty index
GAL‐9: galectin‐9
GBM: glioblastoma multiforme
GMP: granulocyte‐monocyte progenitor
GO: gene ontology
HE: hematoxylin–eosin
HUVEC: human umbilical vein endothelial cell
IFN‐γ: interferon gamma
JAK–STAT: janus kinase‐signal transduction and transcription activation
KEGG: kyoto encyclopedia of genes and genomes
LDH: lactate dehydrogenase
LGG: brain lower grade glioma
LSK: Lin^−^Sca1^+^c‐Kit^+^
LUSC: lung squamous cell carcinoma
MEP: megakaryocyte‐erythrocyte progenitors
MP: myeloid progenitor
NETs: neutrophil extracellular traps
PAAD: pancreatic adenocarcinoma
PGN: peptidoglycan
RER: respiratory exchange ratio
RIPF: radiation‐induced pulmonary fibrosis
RISI: radiation‐induced skin injury
rmGAL‐9: recombinant mouse GAL‐9
ROS: reactive oxygen species
RT‐qPCR: real‐time quantitative PCR
SD: standard deviation
STAD: stomach adenocarcinoma
TC: total cholesterol
TCGA: the cancer genome atlas
TG: triglyceride
THYM: thymoma
TP: total protein
TSNE: t‐distributed stochastic neighbor embedding
UA: uric acid
UMAP: uniform flow approximation and projection

## 1 Introduction

In recent years, the interactions and crosstalk between organs have gained widespread attention, (Shi et al. 2022; Castillo‐Armengol et al. 2019; Franco et al. 2022; Wu et al. 2018; Wang et al. 2024) especially local injuries that can induce distal dysfunction (Nakashima et al. 2019; Yu et al. 2022; Yousefzadeh et al. 2021; Yang, Hayano, et al. 2023; Hao et al. 2023; Liang et al. 2022). Radiation, as a common injury‐causing element, can induce multiple organ dysfunction and is one of the critical clinical dilemmas currently (Dörr and Meineke 2011; Williams and McBride 2011; Kiang and Olabisi 2019). It severely affects the quality of patients' lives, resulting in a heavy medical and economic burden (Hoogendijk et al. 2019; Dent et al. 2019; Ethun et al. 2017). Skin radiation injury is the most frequently observed, (Christensen et al. 2014; DiCarlo et al. 2020) and about 95% of cancer patients develop radiation‐induced skin injury (RISI) after radiotherapy or nuclear accident casualties (Yang, Ren, et al. 2020; Singh et al. 2016). Prolonged RISI wounds induce multiple organ alterations and then result in the frailty of patients (O'Donovan et al. 2017; Güzelöz and Gök 2023). However, the mechanisms involved are still not clearly elucidated. Thus, the current mechanisms of the pathological features and clinical therapeutic needs of local radiation injury‐induced frailty are hardly reflective, and there is an urgent need to search for new key pathogenic points and interventional therapeutic targets.

Neutrophils play vital roles in the regulation of innate and adaptive immunity against infections. However, neutrophils are in a heterogeneous group (Xie et al. 2020; Jaillon et al. 2020; Alshetaiwi et al. 2020; Fan et al. 2022), and under some pathological conditions, hyperactive neutrophils (Adrover et al. 2019) or a subset population (Jaillon et al. 2020; Maas et al. 2023; Yu et al. 2025; Ng et al. 2024) may trigger systemic inflammation and vascular damage (Kessenbrock et al. 2009; Sangaletti et al. 2012) resulting in multiple organ injuries and promoting disease progression (Adrover et al. 2019; Ng et al. 2024; Kessenbrock et al. 2009; Sangaletti et al. 2012; Crossley et al. 2023) The frailty caused by local radiation injury shows in several ways: the weakness in muscle strength, a significant increase in vulnerability to stress, a risk of developing chronic health disorders and long‐term morbidity in multiple organs (Ethun et al. 2017; O'Donovan et al. 2017; Güzelöz and Gök 2023; Kerstens et al. 2023; Cao et al. 2023; Ness et al. 2017; Ness et al. 2015; Eriksen et al. 2023; Kallenbach et al. 2022; Chemaitilly et al. 2022; de Vries et al. 2022) with persistent low‐density inflammation (Rossi et al. 2021; Devarakonda et al. 2023) increased reactive oxygen species (ROS) level (Rossi et al. 2021) accumulation of senescent cells (Ness et al. 2013) and the abnormally elevated neutrophil (Devarakonda et al. 2023; Zhao et al. 2023; Rabold et al. 2022; Yi et al. 2020; Wu et al. 2016), but the mechanism and potential reasons are unknown. Upon the investigation of current clinical and epidemiological related data, patients with local radiation injury usually presented with myeloid‐biased differentiation of bone marrow (Ghosh et al. 2023) and abnormal splenomegaly (Chavakis et al. 2019; Zaorsky et al. 2017; Yang, Chen, et al. 2020) thus we hypothesized that there is crosstalk that have not yet been found among neutrophils, spleen and bone marrow, which might play a critical role in frailty induced by local radiation injury. Here, we established a murine model with local skin radiation injury and identified a previously unrecognized hyperactive Galectin‐9^high^ (GAL‐9^high^) neutrophil population infiltrating into distant multiple organs to induce injuries. These results have characterized a novel ‘skin‐spleen‐bone marrow‐multiple organs’ axis as a significant contributing factor to exacerbate frailty after local radiation injury and suggest that targeting the hyperactive GAL‐9^high^ neutrophils presents an avenue to treat local radiation‐induced frailty.

## 2 Materials and Methods

### 2.1 Mice

All animal studies were approved by the Institutional Animal Care and Use Committee (IACUC) at Army Medical University, with full consideration of animal welfare principles. Sex matched (8–12 weeks old) cohorts of male or female mice were used for the experiments. C57BL/6J mice were maintained in specific pathogen‐free (SPF) facilities under controlled environmental conditions: ambient temperature 24°C ± 1°C, and 12/12‐h light/dark photoperiod (08:00–20:00 light phase). Housing density was strictly limited to ≤ 4 animals per individually ventilated cage. Standard rodent chow and reverse‐osmosis purified water were provided ad libitum. Intraperitoneal injection (i.p.) of the following drugs: recombinant mouse GAL‐9 (rmGAL‐9) protein (200 μg, R&D Systems #3535‐GA‐050), anti‐GAL‐9 antibody (1 mg, BioXCell#BE0218), or IgG isotype control (1 mg, Abcam #ab172730). C57BL/6J mice were used for local skin radiation, anesthetized with 4% isoflurane (RWD), and fixed with a fixator. The skin on the back of the mice was exposed to the radiation field, and the rest of the area was shielded with a 2 cm‐thick lead sheet. Radiation of 60 Gy was applied at a dose rate of 1.3 Gy/min using an X‐ray generator (Precision).

### 2.2 Histological Analysis

Firstly, the mouse tissues were fixed in 4% paraformaldehyde for 24–48 h. Then, the tissues were transferred to the LEICA ASP300S dehydrator for tissue dehydration, followed by tissue embedding on LEICA HistoCore Arcadia H Paraffin Embedder, and the obtained paraffin blocks were stored in a −20 refrigerator. Tissue sections with a single thickness of 3 μm were obtained on a LEICA HistoCore AUTOCUT paraffin slicer.

For hematoxylin–eosin (HE) staining, sections were first deparaffinized by xylene (100%) and gradient ethanol (100%, 95%, 80%), washed in tap water and distilled water, respectively, for 1 min, and then transferred to hematoxylin staining solution for 2 min, washed in tap water for 1 min, differentiated in 0.6% hydrochloric acid alcohol for a few seconds, washed in tap water for 15 min, and the staining effect was observed under the microscope at any time. Subsequently, the sections were transferred to 1% eosin stain for a few seconds, transferred to graded ethanol (80%, 95%, 100%) for dehydration, and finally transparent in xylene for 10 min before sealing with a drop of neutral gum. Images were acquired using an Olympus VS200 microscope and were analyzed using OLYMPUS OlyVIA (version 3.3).

For Masson staining, the Modified Masson's Trichrome Stain Kit (Solarbio #G1346) was used. Briefly, the sections were placed in fixative overnight, treated with Celestite Blue solution staining for 3 min, washed twice with distilled water, treated with Mayer Hematoxylin solution staining for 3 min, washed twice with distilled water, differentiated for a few seconds in Acid Differentiation solution, washed with tap water for 10 min, treated with Ponceau‐Acid Fuchsin solution for 10 min, washed twice with distilled water, treated with Phosphmolybic Acid solution for 10 min, and treated with Aniline Blue solution for 5 min. After washing off the Aniline Blue solution with Weak Acid solution, the sections were dehydrated by gradient ethanol, transparent with xylene for 10 min, and finally sealed with a drop of neutral gum. Images were acquired using an Olympus VS200 microscope and were analyzed using OLYMPUS OlyVIA (version 3.3). The percentage of positive signal area for each sample was measured using Image J (version 1.54).

For immunofluorescence staining, sections were deparaffinized as described above and immersed in citrate solution for antigen repair for 30 min, blocked with rapid blocking solution, and incubated with the corresponding primary antibody overnight at 4°C. After washing with TBST, the sections were incubated with the corresponding secondary antibody for 2 h at room temperature, followed by blocking of the sections with Antifade Mounting Medium with DAPI. Images were acquired using an FV3000 confocal microscope. The percentage area of positive signal relative to the DAPI signal was measured for each sample using Image J (version 1.54). Negative controls were performed using the same specimens without the addition of primary antibodies. Primary antibodies used were Gal‐9 (1:500, Abcam #ab69630), Myeloperoxidase (1:50, Abcam #ab90810), Histone H3 (1:1000, Abcam #ab281584), Ly6g + Ly6c (1:500, Abcam #ab25377), and CD47 (1:500, Abmart #T55251S).

### 2.3 Flow Cytometry Analysis

Mouse circulating cells were obtained from the mouse inter‐cardium using a 1 mL syringe and ACD anticoagulant (Macklin #A885470) was added, mouse spleen cells were obtained by grinding them into a single‐cell suspension, mouse bone marrow cells were obtained by blowing single‐cell suspensions from the femur and tibia using a 1 mL syringe, and the above cells were centrifuged and lysed erythrocytes for 10 min, and then filtered through a 70 μm cell sieve. Cell counting was performed after centrifugation to ensure 1 × 10^6^ cells per sample. Cells were treated with FcR blocking reagent (Biolegend #156604) to block and were labeled with Zombie Aqua live/dead dye (Biolegend #423101) for 10 min at room temperature, subsequent staining with fluorescently labeled antibodies. For intracellular staining, which was performed using the Foxp3/Transcription Factor Staining Buffer Set (Thermo #00‐5523‐00), followed by flow analysis using an Attune NxT (Invitrogen) instrument. Data were analyzed using FlowJo (version 10.8.1) software.

### 2.4 RT–qPCR

Total RNA isolation was performed using the BGMG Fast RNAex Kit (BIOGROUND #BG0048). First‐strand cDNA synthesis was carried out with 1 μg input RNA using the RevertAid Reverse Transcriptase system (Therm #K1622). Real‐time quantitative PCR (RT–qPCR) amplification reactions were executed with TB Green Premix Ex Taq II (Takara #RR820A), with thermal cycling parameters set as: 95°C for 20 s, followed by 40 cycles of 95°C for 10 s, 60°C for 20 s, and 70°C for 1–10 s. Gene expression quantification was normalized against ACTB, using the Comparative Threshold Cycling (Ct) method.

### 2.5 Serum Biochemical Analysis

Blood was collected from the intercardium of mice using a 1 mL syringe, and centrifuged after 30 min at room temperature, and the upper layer of serum was collected on a Myriad BS‐460 instrument for blood biochemistry testing. Blood was collected from the intercardium of mice using a 1 mL syringe, and centrifuged after 30 min at room temperature, and the upper layer of serum was collected on a Myriad BS‐460 instrument for serum biochemical analysis. The reagents used were AST (Mindray #105‐000443‐00), ALT (Mindray #105‐000442‐00), TP (Mindray #105‐000451‐00), ALP (Mindray #105‐000444‐00), TC (Mindray #105‐000448‐00), TG (Mindray #105‐000449‐00), UA (Mindray #105‐000476‐00), LDH (Mindray #105‐000446‐00).

### 2.6 Isolation and Treatment of Circulating Neutrophils

Approximately 1.5 × 10^5^ circulating neutrophils were obtained by flow sorting or Neutrophil Isolation Kit (Miltenyi #130‐097‐658) in a round‐bottom 96‐well plate with RPMI. Cells were stimulated with media or 10 mg/mL peptidoglycan (PGN) for 2 h. Corresponding drugs such as rmGAL‐9 protein (1.2 μg/mL, R&D Systems #3535‐GA‐050), Lactose (30 mM, MedChemExpress #HY‐B2123), anti‐GAL‐9 (10 μg/mL, BioXCell #BE0218), and IgG isotype control (10 μg/mL, Abcam #ab172730) were incubated for 2 h, and cells were washed and analyzed by flow cytometry.

### 2.7 EccDNA Purification for Visualization on Agarose Gels

To purify extrachromosomal circular DNA (eccDNA), fix cells in 95% (vol/vol) methanol by resuspending cells with 0.5 mL PBS and mixing with 9.5 mL absolute methanol, then put them on ice for 10 min. EccDNA was extracted in an alkaline lysis buffer at pH 11.8. Cells were resuspended in suspension buffer, and 10 mL Pyr buffer was added, and gently mixed by inverting the tube five to ten times. The lysate would become blue; kept for 5 min at room temperature. After neutralization and precipitation, crude extrachromosomal DNA was bound to a silica column (QIAGEN Plasmid Plus Midi Kit, QIAGEN #12943) in binding buffer (buffer BB from the QIAGEN Plasmid Plus Midi Kit). Linearized mtDNA by PacI and digested linear DNA with ATP‐dependent PS DNase (Lucigen #E3110K) for 4–16 h. Next, we extracted eccDNA with phenol: chloroform: isoamyl alcohol (PCI, Acros #327111000) solution (25:24:1) in a Phase Lock Gel tube to minimize DNA loss. Transferred the aqueous phase to a new tube, added 1/10 volume of sodium acetate (3 M, pH 5.5, Thermo #AM9740), added 1 μL glycogen, and three volumes of 200 proof ethanol, mixed, and put at −80°C for at least 30 min. Centrifuged the DNA for 30 min at > 16,400 g at 4°C, washed the pellet once with 1 mL 80% (vol/vol) ethanol. After pipetting out the ethanol, spun down the tube for 30 s at > 16,400 g at 4°C, pipetted out residual ethanol, dried the pellet by leaving the tube lid open for 1–2 min, then the pellet was resuspended with 50 μL 2 mM Tris–HCl 7.0 before the pellet totally dries out. For comparisons of eccDNA production among treatments or genotypes, both total DNA (Quick‐DNA microPrep Plus Kit, Zymo #D4074) and eccDNA were purified from equal numbers of cells, eluted, and loaded onto an agarose gel with equal volume. Toaccurately measure the concentration of eccDNA that is hundreds of base pairs in size, we used a SYBR gold‐based quantification method that could accurately quantify both linear dsDNA and circular dsDNA with a curve generated by linear standards from the Qubit 1× dsDNA HS Assay Kit (Thermo #Q33231). Mixed 1.0 g UltraPure agarose with 100 mL 1× TAE in a microwave flask and microwaved for 3–5 min until the agarose was completely dissolved. Poured the agarose into the preassembled glass plates immediately after the agarose was dissolved, without allowing the solution to cool down. Inserted the comb and let the gel cool down for at least 20 min. Removed the comb after dismounting the gel from the casting chamber, and optionally, removed residual gel pieces within the wells using sharp tweezers. Assembled the gel in the apparatus according to the manufacturer's instructions, then filled the chamber with 1× TAE. Carefully pipetted samples (> 1 ng eccDNA could be visualized) into wells after mixing with loading buffer, and loaded a suitable amount of DNA ladder in an adjacent well. Separated DNA at 80 V for 35 min. Running time may be different depending on the concentration of agarose in the gel. Turned off the power, opened the glass plates, and transferred the gel to a 15‐cm‐diameter plastic dish, then added 50–70 mL 1× TAE. Added 5 μL 10,000× Sybr Gold and shook the gel for > 15 min in the dark. Using Bio Rad (USA) ChemiDoc Mp and SYBR Gold uv590/110 excitation light to visualize gel.

### 2.8 Tissue Whole Genome Purification

To purify the tissue's whole genome, 200–300 μg of tissue was then added to 1 mL of BG DNA extraction lysis buffer (BIOGROUND #BG0046) and mixed thoroughly. Centrifuged at a room temperature of 13,000 rpm for 5 min (if the sample was rich in pigments, a flocculent substance that could not be precipitated and wound in the supernatant, and the pigments would be removed during later cleaning, which did not affect subsequent experiments). Transferred the supernatant to a new 2 mL centrifuge tube, taking care not to suck up the precipitate. Added an equal volume of BGMG for DNA (shaken vigorously before using to evenly disperse the magnetic beads) (confirmed that anhydrous ethanol has been added before using), shaken vigorously 10 times or vortexed for 10 s (vigorously dispersed the nano magnetic beads, otherwise it would affect the purity), and let it stand at room temperature for 30 s. Placed the centrifuge tube containing the mixture in a magnetic frame or other form of magnetic field and let it stand for 10 s until the magnetic beads were completely enriched. Poured out the supernatant in a magnetic field, added 500 μL DNA WB1 (confirm that anhydrous ethanol has been added before using), detached from the magnetic field, shook vigorously 10 times or vortexed for 10 s (vigorously dispersed the nano magnetic beads, otherwise it would affect the purity). Placed the centrifuge tube back into the magnetic frame or other form of magnetic field and let it stand for 10 s. Poured out the supernatant in a magnetic field. Added 500 μL of 75% ethanol (to be prepared), detached from the magnetic field, vigorously oscillated for 10 times or vortexed for 10 s. Placed the centrifuge tube back into the magnetic frame or other form of magnetic field and let it stand for 10 s. Poured out the supernatant in a magnetic field. Added 500 μL of 75% ethanol again, detached from the magnetic field, vigorously oscillated for 10 times or vortexed for 10 s. Placed the centrifuge tube again in a magnetic frame or other form of magnetic field and let it stand for 10 s. Poured out the supernatant in a magnetic field and removed residual ethanol.

### 2.9 EccDNA Purification for Animal Treatment

Purification of whole genome sample eccDNA using plasmid mini AX (A&A Biotechnology #010‐50). Suspended the genome in 600 μL of L1 cell suspension. Added 600 μL of L2 lysis solution and mixed gently. Kept at room temperature for 3 min. Added 600 μL of L3T neutralization solution and gently mixed until the red berries in the lysate disappear. Centrifuged at a speed of 10,000–15,000 rpm for 5 min. Placed plasmid 20 columns into a 20 mL test tube. Installed columns with pipes in the rack. Applied 1 mL of K1 equilibrium solution onto the plasmid 20 column. Waited for the solution to flow through the column. Added 4 mL of K2P washing solution. Waited for the solution to flow through the column. Applied 200 μL K3 elution solution directly onto the plasmid 20 column membrane. Waited for the eluent to flow through the chromatographic column. Transferred plasmid 20 columns to a new 2 mL precipitation tube. Added 1 mL of K3 eluent. Waited for the eluent to flow through the chromatographic column. Removed plasmid 20 columns. Added 800 μL of PM precipitation mixture to the eluted DNA. Mixed the samples by flipping the test tube several times and centrifuged at 10,000 RPM for 10 min. Be careful to discard the supernatant. Be careful not to remove the DNA particles at the bottom of the test tube. Added 500 μL of 70% ethanol. Mixed the samples and centrifuged at 10,000 RPM for 5 min. Be careful to discard the supernatant. Be careful not to remove the DNA particles at the bottom of the test tube. Dried the plasmid DNA particles by air at room temperature for 5 min. Dried DNA particles could be dissolved in 50–150 μL of TE buffer or sterile water. Stored eccDNA at 4°C–8°C.

### 2.10 Efferocytosis Assays

Bone marrow macrophages and circulating neutrophils under different conditions were obtained by flow sorting. The neutrophils were labeled with DIO dye (Beyotime #C1038) at 37°C for 30 min, and subsequently co‐cultured in a SpinSR confocal live cell workstation (Olympus) for 72 h. The image area was analyzed using ImageJ (version 1.54).

### 2.11 Single‐Cell Sequencing Data Analysis

Single‐cell sequencing data were analyzed using Seurat (version 5.0.1) with R (version 4.3.3). Firstly, the data was read in via CreateSeuratObject (min.cells = 3, min.features = 200), and Seurat analysis objects were created, incorporating more than 200, less than 2500 nFeature_RNA, and less than 5% of mitochondrial genes as a quality control tool for subsequent analysis. The expression data were analyzed sequentially for NormalizeData, FindVariableFeatures (method = “vst”, nfeatures = 2000), Scale, RunPCA, FindNeighbors (dims = 1:10), FindClusters (resolution = 1.0), run uniform flow approximation and projection (UMAP), run *t*‐distributed stochastic neighbor embedding (TSNE), etc. In order to reduce batch effects, we used the R package Harmony (version 1.2.0) for integration. Subsequently, gene annotation was performed, and “avg_logFC” greater than 0.5 and “*p*_val_adj” less than 0.05 were selected as differentially expressed genes for gene enrichment analyses, such as gene ontology (GO) analysis and Kyoto Encyclopedia of Genes and Genomes (KEGG) analysis (pvalueCutoff = 0.05, pAdjustMethod = ‘BH’, minGSSize = 10). Analysis of cell–cell interactions relied on CellChat (version 1.6.1). Transcription factor prediction analysis relied on SCENIC (version 1.3.1, which relied on mm10 databases) and decoupleR (version 2.4.0). The Pseudotime analysis relied on Monocle 3 (version 1.3.4). Neutrophil function scores referred to Xie et al. and depended on the ‘AddModuleScore’ function. The Cancer Genome Atlas (TCGA) database analysis relied on packages such as GSVA (version 1.50.5).

### 2.12 Statistical Analysis

Statistical analyses were performed using GraphPad Prism 9.5.1, and the results were expressed as mean ± standard deviation (SD). Comparisons between two groups with homogeneous variance were performed by an unpaired Student's *t*‐test, and comparisons between three groups were performed by one‐way analysis of variance (ANOVA). In cases with two conditions or more, a two‐way ANOVA was employed. The Mann–Whitney *U* test was used in the case of heteroscedasticity. For survival experiments, Kaplan–Meier survival curves were analyzed. As indicated in the Figure legends. Statistical significance thresholds throughout the study were defined as follows: ns not significant; *p < 0.05; **p < 0.01; ***p < 0.001.

## 3 Results

### 3.1 Radiation‐Induced Skin Injury Elicits Frailty and Multiple Organ Dysfunction

**FIGURE 1:**
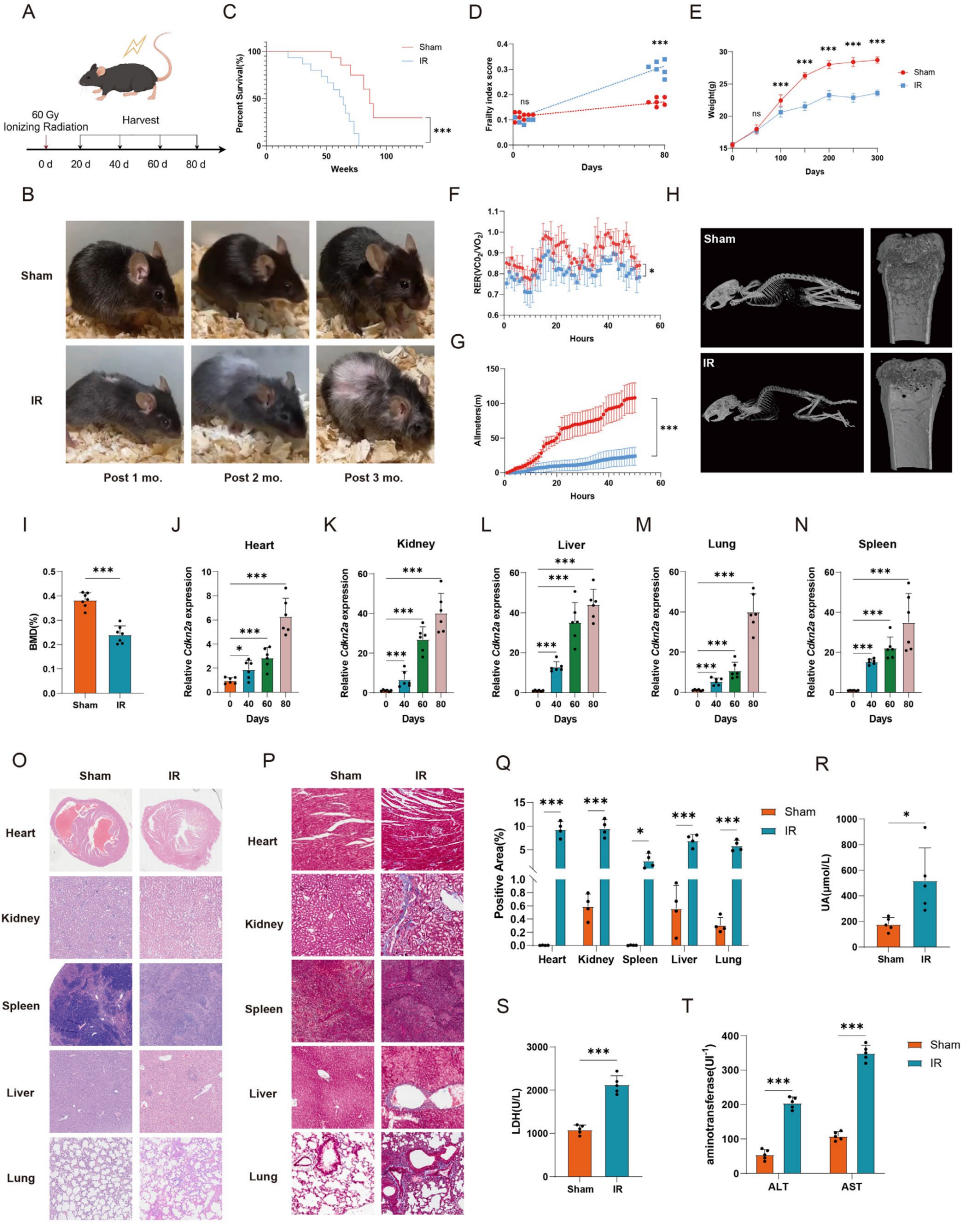
Radiation‐induced skin injury elicits frailty and multiple organ dysfunction. (A) Scheme of local skin radiation protocol (60 Gy, 1.3 Gy/min). (B) Representative plots of mice in the local radiation group and the sham group at different time. (C) Survival curve in the local radiation group and the sham group. *n* = 12. (D) Frailty index score assessment at 80 days post‐radiation and sham group. *n* = 6. (E) Body weights in the local radiation group and the sham group at different time. *n* = 10. (F, G) The mice of the 80 days post‐radiation and sham group were observed in metabolic cages. (F) Respiratory exchange rate (RER, ratio of carbon dioxide consumption to oxygen consumption) and (G) distance of movement of them. *n* = 4. (H, I) Mice and their femur were observed by micro‐CT analysis at 80 days post‐radiation and the sham group. (H) Representative plots of whole body and femur micro‐CT scanning results and (I) bone mineral density (BMD) at 80 days post‐radiation and sham group. *n* = 7. (J–N) *Cdkn2a* mRNA expression relative to *Actin* mRNA housekeeping gene in the (J) heart, (K) kidney, (L) liver, (M) lung, and (N) spleen at 0, 40, 60, and 80 days post‐radiation. *n* = 6. (O–Q) Histopathological assessment of multiple organ injuries. Representative plots of (O) HE staining and (P) Masson staining of heart, kidney, liver, lung, and spleen at 80 days post‐radiation and the sham group. (Q) Quantitative statistical analysis of Masson staining in P. *n* = 4. (R–T) Circulating serum (R) UA, (S) LDH, (T) ALT, and AST assays at 80 days post‐radiation and the sham group. *n* = 5. Data are presented as mean ± SD; each dot represents an individual animal from at least 2–4 independent experiments that used male and female mice. ns, not significant, *p < 0.05, ***p < 0.001. Statistical analyses were calculated using different statistical methods based on data type: Log‐rank test (C), unpaired Student's *t*‐test (D, E, I, J–N, Q–T), and two‐way ANOVA (F, G).

To investigate the critical factors underlying radiation‐induced frailty, we established a murine model of local radiation injury by exposing the mice to a single dose of 60 Gy X‐ray radiation to the dorsal skin at a rate of 1.3 Gy/min, which conformed to IWAKAWA's irradiation protocol (Iwakawa et al. 2003) and previous studies (Ma et al. 2023; Schwentker et al. 1998; Huang et al. 2013; Chen et al. 2024). The progression of radiation‐induced injury was systematically evaluated (Figure 1A and Figure S1A). Characteristic manifestations of frailty include weight loss, reduced metabolic rate, premature aging, and multiple organ injuries. Comparative analysis revealed that the radiation mice exhibited whitening and loss of skin hair on the back and head, and their bodies were curled up (Figure 1B). Following local radiation, we observed an increase in murine mortality (Figure 1C). The frailty index (FI) reflects the overall health status of the body, considering multidimensional parameters including body weight dynamics, coat condition, grip strength, mobility, and other aspects. Notably, radiation mice exhibited substantially higher mean FI scores compared to the sham group (Figure 1D). Mean body weight, subepidermis thickness, and adipose volume were significantly lower in the radiation group than in the sham group (Figure 1E and Figure S1B–D). Premature aging murine models appeared with accelerated cataracts, validating the model's fidelity (Figure S1E). Respiratory Exchange Ratio (RER) refers to the ratio of carbon dioxide excretion to oxygen consumption. At the 80 days post‐radiation, the radiation group exhibited significantly depressed RER values compared with the sham group, indicating that the metabolic rate in the local radiation group was lower (Figure 1F). The motor ability assessment experiment showed that the willingness to exercise was significantly reduced in the radiation group (Figure 1G). Micro‐CT quantification demonstrated exacerbated bowed back and deteriorated osteoporosis in the radiation group compared to the sham group, as evidenced by three‐dimensional reconstruction of vertebral architecture analysis (Figure 1H,I and Figure S1F–G). The above data initially suggested that mice developed frailty after local skin radiation.

Further, we evaluated radiation‐induced premature aging and multiple organ dysfunction. *Cdkn2a* and *Cdkn1a* mRNA expression were quantified across heart, kidney, liver, lung, and spleen, and the results showed that the mRNA levels were consistently elevated at 40 days, 60 days, and 80 days after radiation among multiple organs (Figure 1J–N and Figure S2A–E). Immunofluorescence staining results showed that the expression of P16 in multiple organs was significantly increased after radiation (Figure S2F,G). HE staining showed that the heart of the radiation mice was significantly enlarged, with myocardial hypertrophy, reduced ventricular cavity, and hypertrophy of individual cardiomyocytes being observed; renal histopathology demonstrated diffuse glomerulopathy characterized by atrophy, vacuolated changes, glomerulonephric solid changes, and fibrotic changes of cortical parenchyma; the spleen showed partial loss of splenic nodules and partial fibrotic changes in the splenic parenchyma; the liver showed extensive vacuolated hepatocyte‐like changes and necrotic punctate foci; the lung showed thickening of alveolar walls, hyaline‐like changes, and fibrotic changes (Figure 1O and Figure S2H). Besides, Masson trichrome analysis revealed distinct fibrotic patterns across multiple organ systems at 80 days post‐radiation: myocardial interstitial fibrosis with multifocal collagen deposition; extensive striated fibrosis in the kidney with distribution along the glomeruli and tubules; increased connective tissue in the trabeculae of the spleen; vacuolated changes of hepatocytes and fibrotic changes along the central hepatic vein in the liver; and significant thickening of the alveolar wall and fibrotic changes along the alveolar wall in the lung, suggesting that multiple organs were injured after local skin radiation in mice, which was consistent with the HE results (Figure 1P,Q). Oil red O staining of the liver showed that the positive area was significantly increased in radiation mice (Figure S2I–J). In contrast to the decrease in body weight, the spleen weight/body weight and heart weight/body weight were increased in the radiation group (Figure S2K,L). Blood biochemical tests revealed marked elevation of hepatic indices including aspartate aminotransferase (AST), alanine aminotransferase (ALT), total protein (TP), and alkaline phosphatase (ALP) compared with the sham group, and significant reduction in lipid markers including total cholesterol (TC) and triglyceride (TG), while renal/cardiovascular parameters demonstrated pathological increases among indices including uric acid (UA) and lactate dehydrogenase (LDH) (Figure 1R–T and Figure S2M–P). These results further indicated that multiple organs in the radiation mice showed dysfunction. To exclude the effect of skin trauma on the results in mice, we performed an analysis of *Cdkn2a* and *Cdkn1a* mRNA expression levels across multiple organs in a dorsal excision full‐thickness skin wound murine model, and the results showed that there was no significant change (Figure S3). These collectively demonstrated that local skin radiation induced a clinically relevant frailty in murine models.

### 3.2 Radiation‐Induced Sustained Elevation of Circulating Neutrophils Couples With Bone Marrow Myeloid‐Biased Differentiation and Splenic Extramedullary Hematopoiesis

**FIGURE 2:**
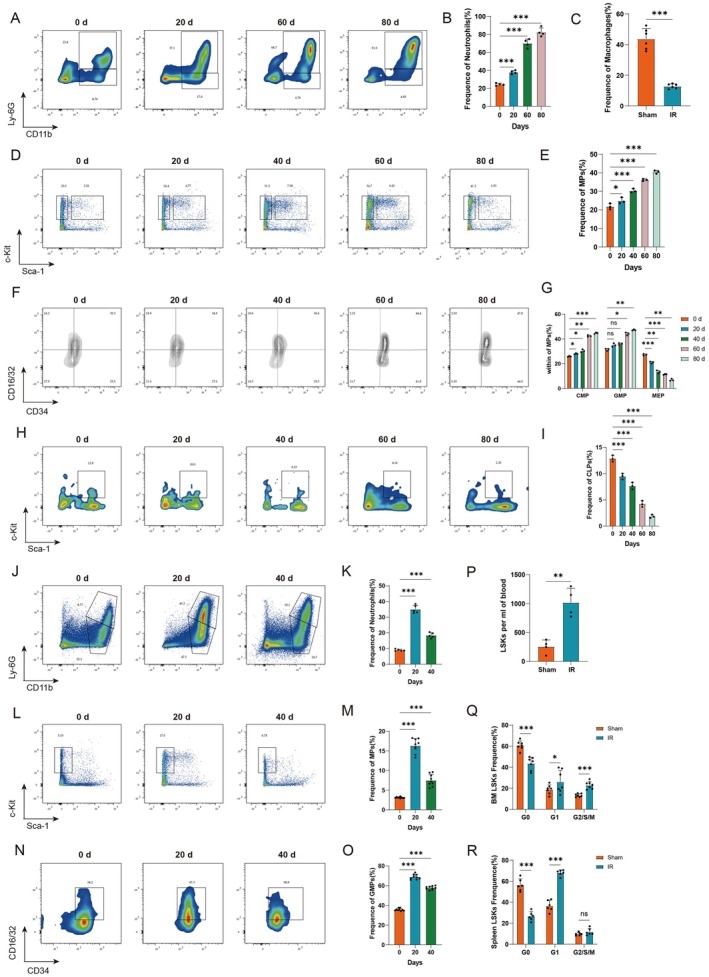
Radiation‐induced sustained elevation of circulating neutrophils couples with bone marrow myeloid‐biased differentiation and splenic extramedullary hematopoiesis. (A–C) Assessment of the frequency of circulating immune cells at different time post‐radiation. (A, B) Representative flow plots and frequency of circulating neutrophils (CD11b^+^ Ly‐6G^+^) and (C) the frequency of circulating macrophages (CD11b^+^ F4/80^+^). *n* = 4–6. (D–I) Assessment of bone marrow hematopoietic progenitor cells frequency at different time post‐radiation. Representative flow plots and frequency of bone marrow (D, E) MP cells (Lin^−^ CD127^−^ Sca‐1^−^ c‐Kit^+^), (F, G) CMP cells (Lin^−^ CD127^−^ Sca‐1^−^ c‐Kit^+^ CD34^+^ CD16/32^−^), GMP cells (Lin^−^ CD127^−^ Sca‐1^−^ c‐Kit^+^ CD34^+^ CD16/32^+^), MEP cells (Lin^−^ CD127^−^ Sca‐1^−^ c‐Kit^+^ CD34^−^ CD16/32^−^), and (H, I) CLP cells (Lin^−^ CD127^+^ Sca‐1^+^ c‐Kit^+^). *n* = 3. (J, K) Representative flow plots and frequency of splenic neutrophils (CD11b^+^ Ly‐6G^+^). *n* = 5. (L–O) Assessment of splenic hematopoietic progenitor cells frequency at different time post‐radiation. Representative flow plots and frequency of splenic (L, M) MP cells (Lin^−^ Sca‐1^−^ c‐Kit^+^), and (N, O) GMP cells (Lin^−^ Sca‐1^−^ c‐Kit^+^ CD34^+^ CD16/32^+^). *n* = 9. (P–R) Assessment of LSK cells frequency and their cell cycle in the local radiation group and the sham group. (P) Circulating LSK cells (Lin^−^ Sca‐1^+^ c‐Kit^+^) frequency and the cell cycle of (Q) bone marrow LSK cells (Lin^−^ Sca‐1^+^ c‐Kit^+^) and (R) splenic LSK cells (Lin^−^ Sca‐1^+^ c‐Kit^+^). *n* = 4–7. Data are presented as mean ± SD; each dot represents an individual animal from at least 2–3 independent experiments that used male and female mice. ns, not significant, *p < 0.05, **p < 0.01, ***p < 0.001. Statistical analyses were calculated using different statistical methods based on data type: Unpaired Student's *t*‐test (C, P), one‐way ANOVA (B, E, I, K, M, O), and two‐way ANOVA (G, Q, R).

Local skin radiation induced frailty with multiple organ injuries and dysfunction in mice; therefore, we initially considered the possibility that local skin radiation induced immune‐related changes in the circulation and then affected the entire body. We performed flow cytometry analysis on circulating immune cells of mice after radiation, and the results revealed that circulating neutrophils were continuously elevated in the 20 days, 60 days, and 80 days after radiation (Figure 2A,B). However, the circulating T cells in the 80 days after radiation were lower than those in the sham group (Figure S4A), and the ratio of CD4^+^/CD8^+^ cells among T cells was significantly lower (Figure S4B). The proportion of Treg cells showed no significant change before and after radiation (Figure S4C), and the circulating macrophages were reduced (Figure 2C and Figure S4D). As the primary site of immune cell production in mammals, bone marrow plays a critical role in hematopoietic regulation. To investigate the underlying mechanism of elevated circulating neutrophils, we conducted flow cytometry analysis on bone marrow cells from radiation mice. The results demonstrated a progressive increase of myeloid progenitor (MP) cells (Figure 2D,E), common myeloid progenitor (CMP) cells, and granulocyte‐monocyte progenitor (GMP) cells, while megakaryocyte‐erythrocyte progenitor (MEP) cells (Figure 2F,G) and common lymphoid progenitor (CLP) cells (Figure 2H,I) gradually decreased within the bone marrow at 20, 40, 60, and 80 days post‐radiation. Bone marrow lymphoid lineage‐associated progenitors decreased, and myeloid lineage‐associated progenitors increased in a time‐dependent manner, suggesting a progressively worsening myeloid‐biased differentiation in the bone marrow.

The spleen plays an important role in stress hematopoiesis as the main extramedullary hematopoietic immune organ. Results showed a gradual rise in neutrophils starting from day 20 post‐radiation, reaching a significant increase in circulating neutrophils 80 days post‐radiation. Furthermore, at 20 days post‐radiation, we observed a progressive myeloid‐biased differentiation in the distal bone marrow and a marked increase in the weight of the spleen (Figure S2K). Consequently, we consider the abnormal changes in the spleen at 20 days post‐radiation to be of particular significance, and we chose to assess changes in splenic immune cells at this time. Flow cytometry analysis of splenic immune cells showed a significant increase in splenic neutrophils (Figure 2J,K) and macrophages (Figure S4E), including circulating recruited and splenic resident macrophages (Figure S4F). Splenic T cells were significantly reduced (Figure S4G), including a significant reduction in the CD4^+^/CD8^+^ ratio (Figure S4H), while there was no significant change in the proportion of Treg cells (Figure S4I), with a trend consistent with the changes in circulating immune cells except macrophages. Further flow cytometric analysis of splenic hematopoietic stem progenitor cells showed that MP cells (Figure 2L,M) and GMP cells were dramatically increased (Figure 2N,O) in the spleen. To further validate the splenic extramedullary hematopoietic, flow cytometric analysis of bone marrow C‐X‐C motif chemokine receptor (CXCR) 4^+^ Lin^−^ Sca1^+^ c‐Kit^+^ (LSK) cells was performed, and the results showed that it was significantly higher in the radiation group than in the sham group at 20 days (Figure S4J), and LSK cells in the circulation were significantly increased (Figure 2P). Flow cytometric analysis of the cell cycle of bone marrow and spleen LSK cells showed a decrease in bone marrow and spleen LSK cells in the G0 phase and an increase in cells in the G1 phase, whereas the bone marrow cells in the G2/S/M phase and the Ki67‐positive bone marrow and spleen LSK cells increased (Figure 2Q,R and Figure S4K–N). To further investigate the relationship between the radiation dose and the above phenotypes, we lowered the dose to 20 Gy and 40 Gy respectively, and flow cytometric analysis of the bone marrow was performed at 80 days (Figure S5A). The results indicated that with the increase of radiation dose, MP cells, CMP cells, and GMP cells showed dose‐dependent escalation in the bone marrow, while MEP cells and CLP cells decreased gradually (Figure S5B–G). Therefore, 60 Gy was chosen for the follow‐up studies. In summary, radiation mice demonstrated time‐ and dose‐dependent myeloid‐biased differentiation in bone marrow, concurrent with robust EMH exhibiting myeloid bias in spleen. The sustained pathological elevation of circulating neutrophils throughout the observation period suggested these innate immune effectors constituted pivotal mediators linking hematopoietic dysregulation to systemic frailty and multiple organ injuries.

### 3.3 Single‐Cell Sequencing Reveals a Subpopulation of Hyperactive GAL‐9^high^ MA2 Neutrophils in Circulation

**FIGURE 3:**
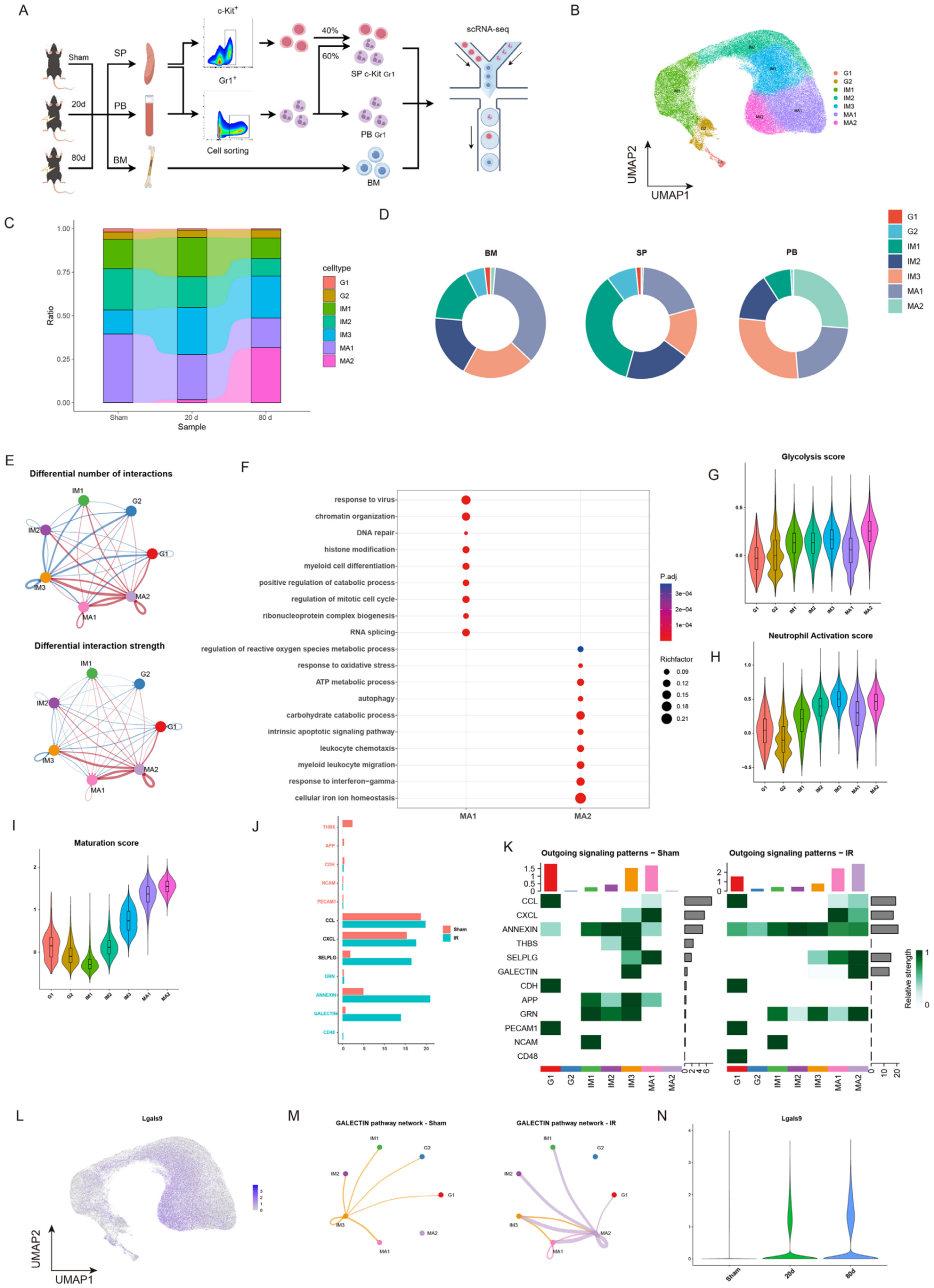
Single‐cell sequencing reveals a subpopulation of hyperactive GAL‐9^high^ MA2 neutrophils in circulation. (A) Scheme of single‐cell sequencing of circulating, splenic, and bone marrow neutrophils in mice. Spleen, bone marrow, and peripheral blood were taken from mice in the sham group, 20 days post‐radiation, and 80 days post‐radiation. 40% c‐Kit^+^ progenitor cells and 60% Gr‐1^+^ neutrophils were sorted from the spleen. Gr‐1^+^ neutrophils were sorted from the peripheral blood. The bone marrow cells were the total number of cells that were flushed out of the bone marrow lumen. (B) UMAP projection delineating neutrophil, preNeu (G1‐G2), immatureNeu (IM1‐IM3), matureNeu (MA1‐MA2). (C) Changes in the ratio of neutrophils according to the different groups. (D) The composition of each population of circulating, splenic, and bone marrow neutrophils by circle plots. (E) Differential number/strength of interactions with each population of neutrophils in the local radiation group relative to the sham group by CellChat analysis (red means higher, blue means lower, and the width of the line represents the interaction strength). (F) Pathway enrichment between MA1 and MA2 neutrophils by bubble plots. (G–I) (G) Glycolysis score, (H) neutrophil activation score, and (I) maturation score for each population of neutrophils by violin plots. (J) The main neutrophil interaction pathways by CellChat analysis for the sham group and 80 days post‐radiation. (K) The main Outgoing signaling patterns of each population of neutrophils in the sham group and 80 days post‐radiation by CellChat analysis. (L) Lgals9 expression in neutrophils UMAP of mice (Figure 3B). (M) Galectin pathway network's enrichment intensity of the sham group and 80 days post‐radiation in each population of neutrophils by CellChat analysis. (N) Lgals9 expression in the sham group, 20 days post‐radiation, and 80 days post‐radiation by violin plots.

To further characterize the underlying mechanism for the abnormal elevation of neutrophils in circulation after radiation, single‐cell RNA sequencing was conducted on bone marrow, splenic, and circulating neutrophils across sham group and radiation groups at 20‐day and 80‐day time, with detailed sequencing protocols referenced to Xie et al. (2020) to clarify the individual developmental order and the background of their origins and to characterize the heterogeneity of the neutrophils (Figure 3A). After stringent quality control, we obtained 111,299 high‐quality neutrophils with an average of 1146 genes per cell profile, and a total of 20,895 genes were detected in all cells. UMAP analysis showed that neutrophils from bone marrow, spleen, and circulation were clustered according to their developmental status into pre‐neutrophils (G1, G2), immature neutrophils (IM1, IM2, IM3), and mature neutrophils (MA1, MA2) (Figure 3B and Figure S6A–D), and the main features of each population were described (Figure S6E–I). Notably, a distinct neutrophil subpopulation designated MA2 (Figure 3C and Figure S6J) emerged exclusively at 80‐day post‐radiation compared to sham controls, which was predominantly distributed in the circulation rather than in the bone marrow and spleen (Figure 3D and Figure S6K). MA1, a subpopulation of terminally differentiated neutrophils in the physiological state, has functions such as responding to viruses, promoting myeloid differentiation and regulation of metabolism, etc., whereas MA2 neutrophil subpopulation, emerging in the circulation after radiation, showed active interaction among pathways including oxidative stress, autophagy, endogenous apoptotic, leukocyte recruitment and chemotaxis, and response to interferon gamma (IFN‐γ) pathways (Figure 3E,F and Figure S7A). However, the MA2 neutrophil subpopulation was markedly more capable than the MA1 subpopulation in terms of glycolysis, neutrophil activation and maturation, phagocytosis, activation of transcription factors, formation of neutrophil extracellular traps (NETs), and activation of Toll‐like receptors. While the MA2 subpopulation was less capable than the MA1 subpopulation in terms of specific granule and cellular proliferation (Figure 3G–I, and Figure S7B–E). Therefore, we speculated that the MA2 neutrophil was hyperactive with high aggressiveness and pro‐inflammatory capacity, which might be one of the key factors inducing frailty.

To further clarify the function and molecular marker of the MA2 neutrophil subpopulation, we performed CellChat analysis, and the number/strength of pathway interactions in neutrophils were significantly increased after radiation, most notably in the MA2 subpopulation (Figure S7F,G). Under physiological conditions, the MA1 neutrophil subpopulation had the highest strength of both output and input pathways. However, after radiation, we found that the MA2 subpopulation exceeded the MA1 subpopulation in both capacities (Figure S7H). We found that radiation exposure triggered significant activation of the GALECTIN, ANNEXIN, and SELPG signaling pathways, with the GALECTIN pathway exhibiting the most pronounced upregulation, about 10‐fold induction (Figure 3J). Neutrophils acted as both senders and receivers of the signals (Figure 3K and Figure S7I) with specific ligand receptors as Lgals9‐Cd45/Cd44 (Figure S7J). Further analysis showed that GAL‐9 was mainly expressed in the MA2 subpopulation (Figure 3L and Figure S7K), and the GAL‐9 pathway was significantly up‐regulated during interactions between the MA2 subpopulation and other neutrophil subpopulations (Figure 3M and Figure S7L). We analyzed the time series of GAL‐9 and found that neutrophil GAL‐9 expression was low under physiological conditions, with a gradual increase from day 20 to day 80 (Figure 3N). We analyzed two other significantly up‐regulated pathways after radiation, the ANNEXIN pathway and the SELPG pathway, and found that they were activated both under physiological conditions and after radiation (Figure S8); therefore, they were not suitable as a post‐radiation‐specific molecular marker. Thus, we selected GAL‐9 as a molecular marker specific for the MA2 subpopulation. In summary, we identified a subpopulation of neutrophils, MA2, which were in a state of hyperactivation, highly aggressive and proinflammatory, and characterized by a high expression of GAL‐9, after analyzing the single‐cell results of neutrophils in the post‐radiation circulation. Previous studies have shown that exogenous addition of GAL‐9 protein promoted neutrophil adhesion, migration, (Iqbal et al. 2022; Wiersma et al. 2019) and degranulation, (Vega‐Carrascal et al. 2014) increases NADPH oxidase activity, (Vega‐Carrascal et al. 2014) and enhances factor secretion (Wiersma et al. 2019; Steichen et al. 2015; Bozorgmehr et al. 2021). Therefore, we hypothesized that the newly emerged GAL‐9^high^ MA2 subpopulation might be one of the key mediators contributing to the frailty.

### 3.4 GAL‐9^high^ Neutrophils Are Crucial Mediators for Inducing Frailty After Local Radiation Injury

**FIGURE 4:**
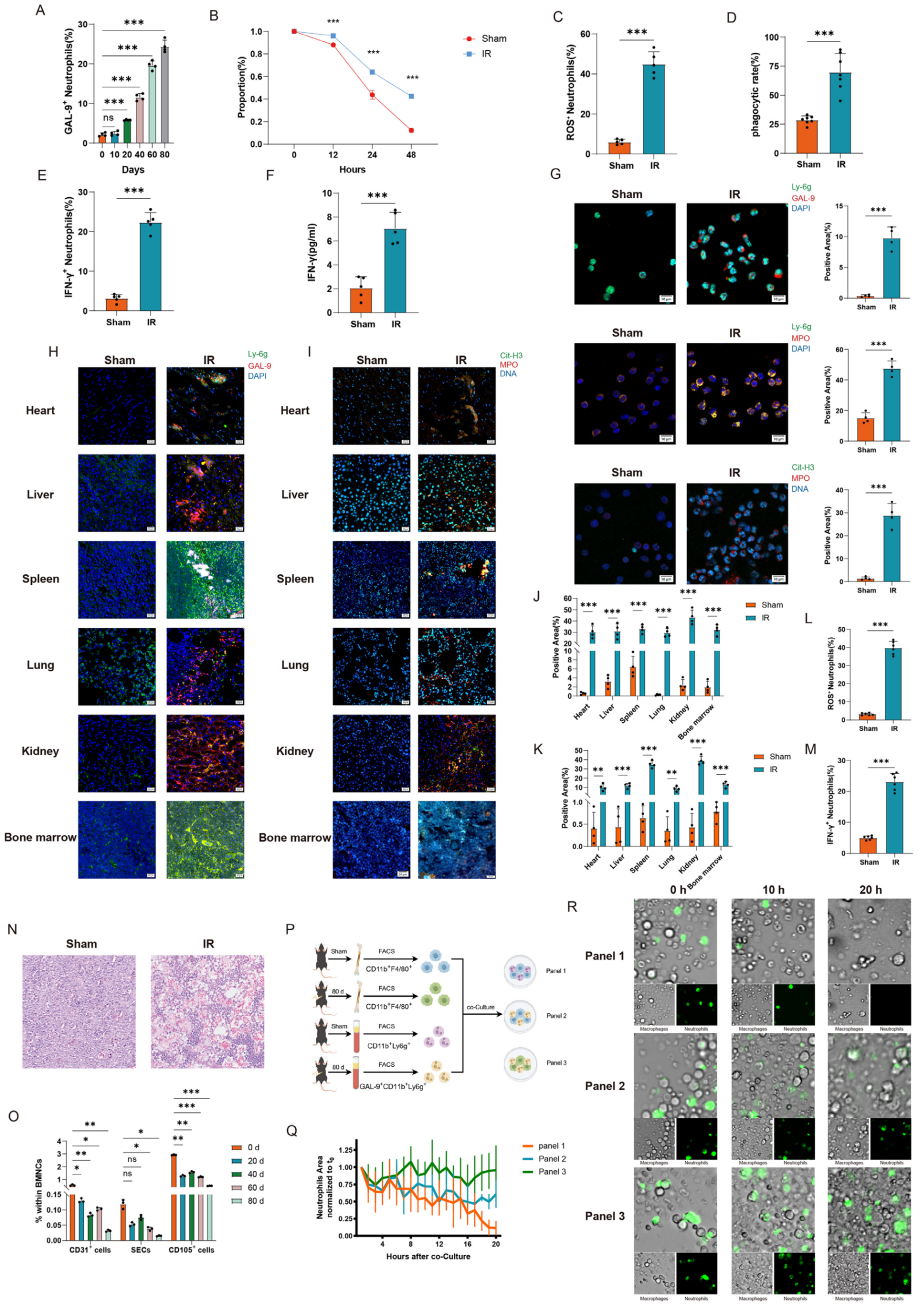
GAL‐9^high^ neutrophils are crucial mediators for inducing frailty after local radiation injury. (A) Circulating GAL‐9^high^ neutrophils at different time post‐radiation. *n* = 4. (B) The survival rate of circulating neutrophils in the sham group and circulating GAL‐9^high^ neutrophils 80 days post‐radiation was analyzed by the CCK‐8 kit. (C–E) Cultured circulating neutrophils in the sham group and GAL‐9^high^ neutrophils 80 days post‐radiation were treated with media with 10 mg/mL PGN for 2 h, after which (C) ROS, (D) phagocytic rate, and (E) IFN‐γ were quantified. *n* = 5–6. (F) Circulating IFN‐γ protein level by ELISA kits. *n* = 5. (G) Cultured circulating neutrophils in the sham group and GAL‐9^high^ neutrophils 80 days post‐radiation were treated with media with 50 mg/mL PGN for 4 h, after which MPO and NETs were visualized by cell smears. *n* = 4. (H–K) Representative plots and statistics of (H, J) GAL‐9^high^ neutrophil and (I, K) NETs expression in multiple organs by immunofluorescence. *n* = 4. (L, M) Bone marrow neutrophils in the sham group and 80 days post‐radiation were treated with media or 10 mg/mL PGN for 2 h, after which (L) ROS and (M) IFN‐γ were quantified. *n* = 6. (N) Representative plots of HE staining of bone marrow in the sham group and 80 days post‐radiation. (O) The frequence of bone marrow endothelial cells (CD45^−^ Ter119^−^ CD31^+^ CD105^−^), SECs (CD45^−^ Ter119^−^ CD31^+^ CD105^+^), and stromal cells (CD45^−^ Ter119^−^ CD31^−^ CD105^+^) at different time post‐radiation. *n* = 3. (P–R) Assessment Scheme of different groups of bone marrow macrophages phagocytosed neutrophils by confocal live cell station imaging in vitro. (P) The scheme, (Q) the survival area statistics of neutrophils, and (R) the representative plots of phagocytosis in different groups at different time were shown. Data are presented as mean ± SD; each dot represents an individual animal from at least 2–4 independent experiments that used male and female mice. ns, not significant, *p < 0.05, **p < 0.01, ***p < 0.001. Statistical analyses were performed using unpaired Student's *t*‐test (B–G and J–M), one‐way ANOVA (A), and two‐way ANOVA (O).

To further investigate the mechanisms underlying radiation‐induced frailty, we first validated the aforementioned sequencing findings. Circulating GAL‐9^high^ neutrophils showed no significant difference compared to sham controls at 10 days post‐radiation, but exhibited progressive accumulation at 20, 40, 60, and 80‐day post‐radiation (Figure 4A and Figure S9A). We performed fluorescence‐activated cell sorting (FACS) to isolate circulating GAL‐9^high^ neutrophils at 80 days post‐radiation. We found lifespan extension (Figure 4B) compared with the sham group. Pre‐screening transcriptional profiling revealed upregulated IFN‐γ pathway activity in MA2 neutrophil subsets (Figure 3F). Functional profiling revealed ROS^+^ subpopulation expansion (Figure 4C and Figure S9B), phagocytic capacity elevation (Figure 4D), and IFN‐γ expression elevation (Figure 4E and Figure S9C) compared with the sham group after treating with PGN. Furthermore, ELISA results showed that the circulating IFN‐γ level was elevated 80 days after radiation (Figure 4F). Smear staining of sorted GAL‐9^high^ neutrophils after treatment with PGN showed that the levels of MPO and NETs were significantly higher in the radiation group (Figure 4G). Previous studies have shown that overexpression of NETs can lead to multiorgan injuries (Shiratori‐Aso et al. 2023; Cho et al. 2023; Bukong et al. 2018; Grégoire et al. 2018). Furthermore, immunofluorescence staining results in multiple organs showed a significant increase in infiltrating GAL‐9^high^ neutrophils (Figure 4H–J), MPO (Figure S9D–E), and NETs levels (Figure 4I–K). Physiological clearance of aging neutrophils depends on their bone marrow homing for macrophage‐dependent efferocytosis (Loh and Vermeren 2022; Seyfried et al. 2020; Casanova‐Acebes et al. 2013). Thus, we detected the effect of GAL‐9^high^ neutrophil homing on the bone marrow. We found that the levels of GAL‐9^high^ neutrophils, MPO, and NETs in the bone marrow were significantly higher (Figure 4H–K and Figure S9D,E), and the ROS (Figure 4L and Figure S9F) and IFN‐γ level (Figure 4M and Figure S9G) were significantly elevated in bone marrow after treating with PGN, similar to the circulating GAL‐9^high^ neutrophil profile. These inflammatory factors might be the key factors contributing to the development of a myeloid‐biased differentiation in bone marrow. HE staining of bone marrow showed that the number of cells was significantly reduced in the radiation group in the bone marrow niche, which was filled with adipose vacuoles (Figure 4N and Figure S9H). Non‐immune cells in the bone marrow were significantly reduced (Figure S9I,J), including endothelial cells, SECs, and stromal cells (Figure 4O and Figure S9K), which indicated that the bone marrow microenvironment was markedly disrupted. Consistent with single‐cell sequencing results, the above data revealed that the GAL‐9^high^ neutrophils were in a hyperactive state, infiltrating into multiple organs to result in injuries and disturbing the bone marrow microenvironment through the secretion of excessive NETs, IFN‐γ, etc.

The homing aging neutrophils should have been cleared by bone marrow macrophages, which was opposite to the phenotype that the presence of large numbers of GAL‐9^high^ neutrophils, the microenvironment was markedly disrupted, and a time‐dependent myeloid‐biased differentiation of the bone marrow, so we presumed that the obstacle to bone marrow macrophages' clearance of GAL‐9^high^ neutrophils. Thus we isolated circulating neutrophils with bone marrow‐resident macrophages in the sham group, and GAL‐9^high^ circulating neutrophils paired with bone marrow macrophages at 80 days post radiation, and then cultured them in a mixture and obtained macrophage‐to‐neutrophil phagocytic videos (Figure 4P) in a live‐cell workstation. The results showed that bone marrow macrophages in the sham group could phagocytose circulating neutrophils in the sham group (Panel 1, Figure 4Q,R and Video S1); while the phagocytosis of GAL‐9^high^ neutrophils by bone marrow macrophages from both the sham group and the radiation group was slowed down (Panel 2,3, Figure 4Q,R; Videos S2 and S3). The above data revealed that GAL‐9^high^ neutrophils homing to the bone marrow resisted clearance by bone marrow macrophages.

### 3.5 GAL‐9^high^ Neutrophils Adoptive Transfer and TCGA Database Analysis

**FIGURE 5:**
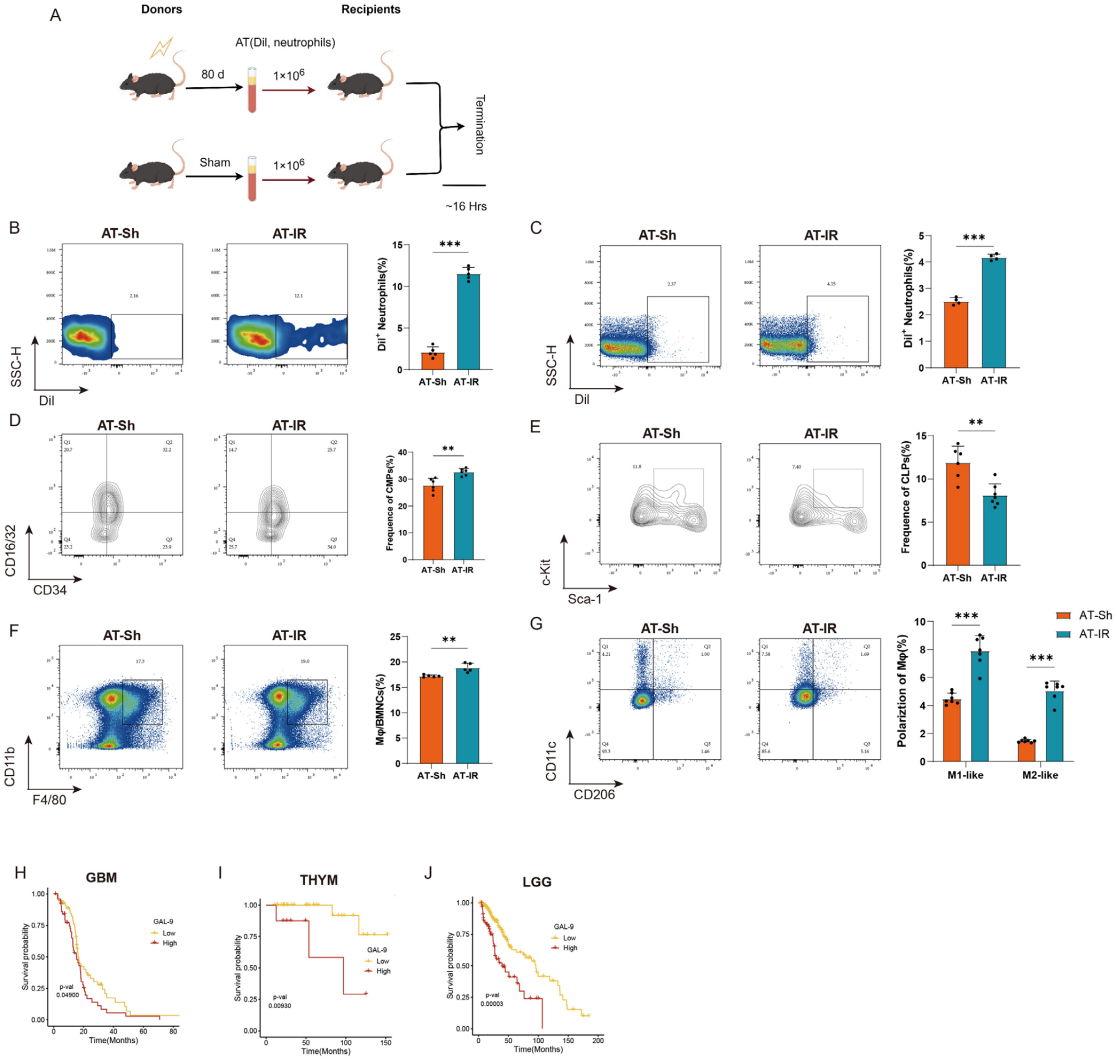
GAL‐9^high^ neutrophils adoptive‐transfer assay and TCGA database analysis. (A–G) Assessment of the effect of circulating neutrophils in sham group and 80 days post‐radiation adoptive‐transfer to recipients, and the neutrophils were marked by Dil. (A) The scheme was shown. Representative flow plots and frequency of Dil^+^ neutrophils in (B) circulation and (C) bone marrow, and the frequency of bone marrow (D) CMP cells, (E) CLP cells, (F) macrophages and their (G) polarization state in different groups of recipients. *n* = 4–7. (H–J) K‐M curve analysis of GAL‐9^high^ neutrophils‐related genes in (H) GBM, (I) THYM and (J) LGG in TCGA database. *p*‐value was shown. Data are presented as mean ± SD, each dot represents an individual animal from at least 2–3 independent experiments that used male and female mice. **p < 0.01, ***p < 0.001. Statistical analyses were performed using unpaired Student's *t*‐test (B–F), two‐way ANOVA (G) and log‐rank test (H–J). *p*‐value was shown.

Our previous data have suggested that GAL‐9^high^ neutrophils were key mediators of damage to multiple organs, disrupted the bone marrow microenvironment, resisted clearance by bone marrow macrophages, and induced a persistent myeloid‐biased differentiation and frailty after local radiation. To further clarify the effect of GAL‐9^high^ neutrophils on myeloid‐biased differentiation, we performed a neutrophil adoptive‐transfer assay. Specifically, circulating GAL‐9^high^ neutrophils in the radiation group were incubated in vitro with Dil to label them, and then transferred into recipient mice, which were evaluated after 16 h (Figure 5A). The results showed that Dil^+^ neutrophils in both circulation (Figure 5B) and bone marrow (Figure 5C) were significantly higher in recipient mice transplanted with GAL‐9^high^ neutrophils than in control mice, suggesting that GAL‐9^high^ neutrophils had a longer lifespan or were more resistant to clearance. Bone marrow CMP cells were increased (Figure 5D), and CLP cells were decreased (Figure 5E), indicating myeloid‐biased differentiation in recipient mice. Bone marrow macrophages were increased compared to controls (Figure 5F), among which an increase in M_1_‐like macrophages was observed (Figure 5G). Consistent with the previous data, this further validates the perturbation of the bone marrow environment by GAL‐9^high^ neutrophils.

It is an important issue to address how GAL‐9^high^ neutrophils infiltrate multiple distant organs. There are several studies that have shown that formyl peptide receptor 1 (FPR‐1) (Scharf et al. 2024; Leslie et al. 2020), very‐late antigen‐4 (VLA‐4) (Futosi et al. 2013; Neumann et al. 2015), CXCR2 (Adrover et al. 2019; Xie et al. 2024; Hussain et al. 2025), CXCR4 (Ballesteros et al. 2020; Zhang et al. 2015; Sainz and Sata 2008), and CD11b (Scott et al. 2020; Fu, Han, et al. 2025) were important regulators of neutrophil recruitment. Based on single‐cell sequencing results in our study, GAL‐9^high^ neutrophils exhibit significantly upregulated chemotaxis‐related pathways (leukocyte chemotaxis, myeloid leukocyte migration, leukocyte transendothelial migration) (Figure 3F and Figure S7A). CellChat analysis revealed significant upregulation of the ‘CXCL2‐CXCR2’ pathway in GAL‐9^high^ neutrophils (Figure S10A). Expression profiling of CXCR2 across neutrophil populations demonstrated the highest expression in GAL‐9^high^ neutrophils (Figure S10B). CXCR2 expression significantly increased at 80 days post‐radiation (Figure S10C). Flow cytometry results showed significantly elevated CXCR2 expression in circulating neutrophils post‐radiation (Figure S10D). Immunofluorescence staining revealed a markedly increased CXCR2^+^ GAL‐9^+^ neutrophils in multiple organs following radiation (Figure S10E). We further validated the expression of another key neutrophil chemokine receptor, CXCR4. Results showed no significant difference in CXCR4 expression among circulating neutrophils post‐radiation (Figure S10F). Consequently, we propose that CXCR2 is involved in playing a role in GAL‐9^high^ neutrophils infiltrating multiple organs.

Tumor patients might become debilitated after radiotherapy, which worsens their clinical prognosis. To clarify the clinical role of GAL‐9^high^ neutrophils, we conducted a prognostic analysis of the TCGA database, incorporating radiation therapy as a screening criterion. It was found that the presence of GAL‐9^high^ neutrophils after radiotherapy in GBM, THYM, and LGG tumors led to a significant increase in their mortality rate (Figure 5H–J), and in ESCA, LUSC, PAAD, and STAD tumors led to a worse prognosis with no significant differences (*p* > 0.05) (Figure S10G–J). This indicated that GAL‐9^high^ neutrophils were related to worse prognosis after radiotherapy, expanding the significance of their clinical implications.

### 3.6 GAL‐9 Protein Is an Important Regulatory Molecule in Neutrophil Hyperactivity

**FIGURE 6:**
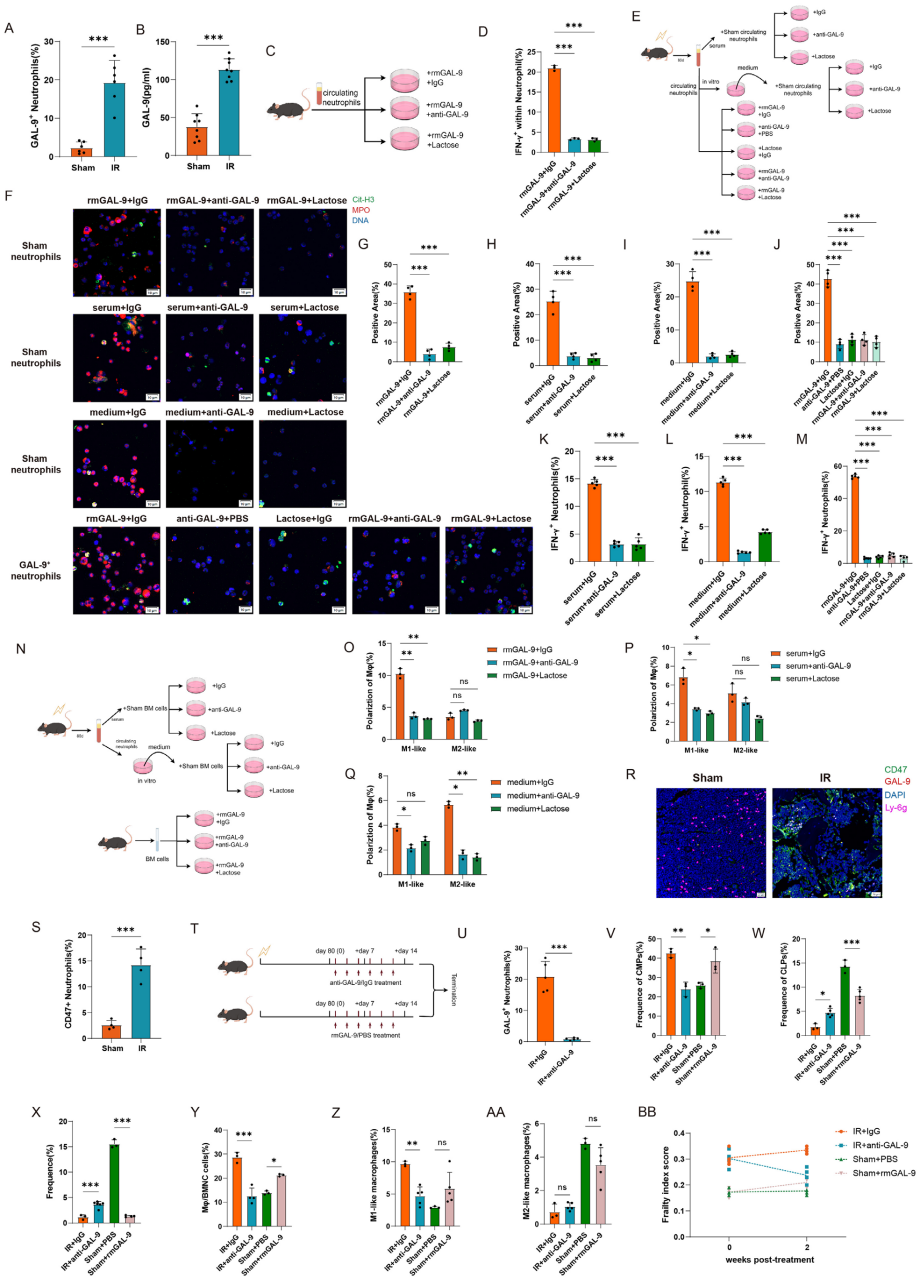
GAL‐9 protein is an important regulatory molecule in neutrophil hyperactivity. (A) Intracellular GAL‐9 protein expression of circulating neutrophils in the sham group and 80 days post‐radiation. *n* = 6. (B) Circulating GAL‐9 protein level by ELISA kits. *n* = 8. (C, D) (C) Scheme and (D) the IFN‐γ expression of circulating neutrophils in the sham group under different conditions. *n* = 3. (E–M) (E) Scheme of the IFN‐γ and NETs expression of circulating neutrophils in the sham group by treatment with (F, H, K) circulating serum at 80 days post‐radiation and (F, I, L) culture supernatant of GAL‐9^high^ neutrophils. (F, J, M) IFN‐γ and NETs expression of GAL‐9^high^ neutrophils 80 days post‐radiation under different stimuli. *n* = 4–5. (N–Q) Assessment of the effect of GAL‐9 protein on the polarization of bone marrow macrophages under different conditions. (N) The scheme and the effect of (O) rmGAL‐9 protein, (P) culture supernatant of GAL‐9^high^ neutrophils, and (Q) circulating serum at 80 days post‐radiation were shown. *n* = 3. (R–S) Representative plots and statistics of bone marrow CD47^+^ neutrophils in the sham group and 80 days post‐radiation. *n* = 4. (T‐BB) Assessment of the reversal effect of GAL‐9 intervention in mice. (T) Scheme of the administration of anti‐GAL‐9 and rmGAL‐9 proteins in the local radiation group and the sham group, respectively. (U) Circulating GAL‐9^high^ neutrophils, bone marrow (V) CMP cells, (W) CLP cells, (X) non‐immune cells, (Y) macrophages, and (Z, AA) their polarization state and (BB) frailty index score were shown after different treatments in the local radiation group and the sham group. *n* = 3–5. Data are presented as mean ± SD; each dot represents an individual animal from at least 2–4 independent experiments that used male and female mice. ns, not significant, *p < 0.05, **p < 0.01, ***p < 0.001. Statistical analyses were performed using unpaired Student's *t*‐test (A, B, S, U), one‐way ANOVA (D, G–M, V–AA), and two‐way ANOVA (O–Q).

To further clarify the mechanism of GAL‐9^high^ neutrophil‐induced frailty, we explored the role of GAL‐9 protein in this process. Interestingly, we found that an upregulation of intracellular GAL‐9 protein in neutrophils (Figure 6A and Figure S11A) and GAL‐9 protein level in circulation (Figure 6B). Taking into account the regulation of GAL‐9 protein in neutrophils (Iqbal et al. 2022; Wiersma et al. 2019; Vega‐Carrascal et al. 2014; Steichen et al. 2015; Bozorgmehr et al. 2021), we hypothesized that GAL‐9 protein might play an important role in the hyperactivity of neutrophils. To verify this hypothesis, we isolated circulating neutrophils from the sham group and treated them with rmGAL‐9 protein and its antibody or lactose for neutralization (Figure 6C), after validating the rmGAL‐9 protein in vitro gradient experiments (Figure S11B). The results showed that rmGAL‐9 protein could increase the neutrophil IFN‐γ^+^ ratio (Figure 6D and Figure S11C) and NETs expression (Figures 6F and 5G), and this effect was inhibited when antibody or lactose was added. Previous studies have indicated that some intracellular proteins of neutrophils were released extracellularly during exerting function, such as MPO, S100A8/A9, NE, etc (Chen et al., 2021; Sprenkeler et al. 2022). Thus, we hypothesized that GAL‐9^high^ neutrophils might release GAL‐9 protein to exert function. To further verify the above hypothesis, we designed three sets of parallel experiments as follows: (1) obtained the serum from the radiation group after 80 days, and co‐cultivated it with the circulating neutrophils of the sham group under different conditions; (2) took the culture medium of the circulating GAL‐9^high^ neutrophils of the radiation group after 80 days, and co‐cultivated it with the circulating neutrophils of the sham group under different conditions; (3) isolated the circulating GAL‐9^high^ neutrophils of the radiation group after 80 days, and treated them under different conditions; and detected the expression of IFN‐γ in neutrophils by flow cytometry, respectively (Figure 6E). The results showed that serum from the radiation group (Figure 6F,H,K and Figure S11D) and culture medium of GAL‐9^high^ neutrophils (Figure 6F,I,L and Figure S11E) could increase the level of IFN‐γ and NETs expression in neutrophils, and these effects were inhibited when anti‐GAL‐9 antibody or lactose was added; GAL‐9^high^ neutrophils can be stimulated by rmGAL‐9 protein towards a dramatic increase in the expression of IFN‐γ and NETs, and the effect disappeared when antibody or lactose was added or when both were treated with rmGAL‐9 protein (Figure 6F,J,M and Figure S11F). The above data revealed GAL‐9 proteins played an important role in neutrophils' hyperactivity, such as the expression of IFN‐γ and NETs, which were released from GAL‐9^high^ neutrophils.

Furthermore, we explored the role of GAL‐9 proteins on bone marrow macrophages. Single‐cell analysis of bone marrow showed that the strengths/numbers of interaction between bone marrow macrophages and neutrophils significantly increased after radiation (Figure S12A). The NF‐κB pathway, NOD‐like receptor pathway, Toll‐like receptor pathway, TNF pathway, the chemotaxis, migration, and activation of leukocytes/myeloid cells were significantly up‐regulated in the 80 days radiation mice's bone marrow macrophages compared with the sham group, indicating that pro‐inflammatory of bone marrow macrophages in the radiation group increased significantly (Figure S12B). We observed that bone marrow macrophages gradually increased in the radiation group (Figure S12C), with a gradual increase of M_1_‐like macrophages and a gradual decrease of M_2_‐like macrophages (Figure S12D). Previous studies have shown that macrophages secreted a range of anti‐inflammatory factors during the efferocytosis of neutrophils, and their own polarization state gradually shifted from M_1_ to M_2_, thereby preventing the further expansion of inflammation (Bukong et al. 2018; Grégoire et al. 2018; Casanova‐Acebes et al. 2013; Li et al. 2023). However, we further evaluated the levels of inflammatory factors in bone marrow macrophages, and the results showed that the expression levels of pro‐inflammatory factors such as *Il‐1b*, *Il‐6*, and *iNos* were significantly elevated in bone marrow macrophages (Figure S12E), whereas the expression levels of pro‐inflammatory resolution related genes, such as *Tgm2*, *Lxra*, *Cd36*, *Lxrb*, and *Ucp2* were decreased (Figure S12F). This is consistent with the single‐cell sequencing results, suggesting that the cell efferocytosis process of bone marrow macrophages after radiation is not completed. The CellChat results showed that the GALECTIN‐related pathway was up‐regulated in the bone marrow after radiation (Figure S12G), in which macrophages were significantly enhanced by the regulation of the GALECTIN pathway (Figure S12H); thus, we hypothesized that GAL‐9 protein might influence bone marrow macrophage function. To verify the above conjecture, we designed a set of parallel experiments: (1) the bone marrow cells of mice in the sham group were cultured under different conditions; (2) the serum of mice 80 days after radiation was cultured with the bone marrow cells of mice in the sham group under different conditions; (3) the circulating GAL‐9^high^ neutrophils of mice 80 days after radiation were cultured in vitro, and the culture medium was then cultured with the bone marrow cells of mice in the sham group under different conditions; and the polarization of bone marrow macrophages was subsequently detected (Figure 6N). The results showed that rmGAL‐9 could promote the polarization of bone marrow macrophages towards M_1_ state, and the polarizing effect was attenuated when anti‐GAL‐9 antibody or lactose was added (Figure 6O and Figure S12I); serum (Figure 6P and Figure S12J) and in vitro culture medium of GAL‐9^high^ neutrophils (Figure 6Q and Figure S12K) from mice 80 days after radiation could promote the polarization of bone marrow macrophages towards M_1_ state, and the polarizing effect was attenuated when anti‐GAL‐9 antibody or lactose was added, suggesting that GAL‐9 protein could influence the function of macrophages to promote their M_1_‐like polarization. To clarify the suppression effect of the clearance on homing neutrophil in the bone marrow, we found that CD47 was enhanced in bone marrow macrophage‐neutrophil interactions (Figure S12L), which was well accepted as a canonical ‘don't eat me’ marker (Liao et al. 2024; Logtenberg et al. 2020; Liu et al. 2023). Immunofluorescence staining results were consistent with single‐cell sequencing results, showing that the expression of CD47 in bone marrow neutrophils was significantly increased after radiation (Figure 6R,S). The expression of bone marrow macrophage‐related genes, including *Sirpa* and *Thbs1*, was also elevated (Figure S12M). The above data revealed that GAL‐9 protein could influence the function of macrophages to promote their M_1_‐like polarization, and GAL‐9^high^ neutrophils resisted clearance by bone marrow macrophages through the expression of molecules such as CD47.

To further clarify that GAL‐9 protein played an important role in the induction of frailty, we intervened in mice that had already developed frailty 80 days after radiation by administering anti‐GAL‐9 antibody for 2 weeks to reverse the above phenotype, and mice in the sham group were given rmGAL‐9 protein for 2 weeks in parallel to evaluate whether there was a worsening effect (Figure 6T). The results showed that administration of anti‐GAL‐9 antibody to radiation mice significantly decreased circulating GAL‐9^high^ neutrophils (Figure 6U), lowered bone marrow CMP cells (Figure 6V and Figure S13A), and increased CLP cells (Figure 6W and Figure S13B), thus reversing the myeloid‐biased differentiation to some extent. In contrast, administration of rmGAL‐9 protein to mice in the sham group increased bone marrow CMP (Figure 6V and Figure S13A) and lowered CLP cells (Figure 6W and Figure S13B), worsening the myeloid‐biased differentiation. Administration of anti‐GAL‐9 antibody to radiation mice restored non‐immune cells in the bone marrow and reduced the damage to the bone marrow microenvironment to a certain extent (Figure 6X and Figure S13C); in contrast, administration of rmGAL‐9 protein to the sham group worsened the damage to the bone marrow microenvironment (Figure 6X and Figure S13C). In terms of bone marrow macrophages, administration of anti‐GAL‐9 antibody to radiation mice decreased bone marrow macrophages (Figure 6Y and Figure S13D) and pro‐inflammatory‐associated M_1_‐like macrophages (Figure 6Z and Figure S13E), and lowered the interference with bone marrow macrophages. In contrast, administration of rmGAL‐9 protein to mice in the sham group elevated bone marrow macrophages (Figure 6Y and Figure S13D) and pro‐inflammatory‐associated M_1_‐like macrophages (Figure 6Z and Figure S13E), worsening the disturbance to the bone marrow microenvironment. However, the above operations did not affect the M_2_‐like bone marrow macrophages (Figure 6AA and Figure S13E). Finally, we evaluated the frailty index of mice, and the results showed that administration of anti‐GAL‐9 antibody to mice in the radiation group lowered the frailty index of mice; on the contrary, administration of rmGAL‐9 protein to mice in the sham group elevated the frailty index of mice (Figure 6BB). The above experiments further clarified the important role of GAL‐9 protein in the process of inducing frailty, and these findings provided potential evidence for further intervention.

The safety analysis of the anti‐GAL‐9 antibody is crucial for evaluating the therapeutic outcomes. In our experiments, no significant adverse reactions were observed in mice administered anti‐GAL‐9 antibody after 200 days (1 mg, administered three times weekly for 2 weeks). There are also several published works to exhibit favorable targeting properties of these anti‐GAL‐9 antibodies, with no mention of safety concerns, such as in pancreatic ductal adenocarcinoma mouse model (Daley et al. 2016), breast cancer model (de Mingo et al. 2018), preeclampsia mouse model (Li et al. 2024), colorectal liver metastases (Wang et al. 2025), etc. We conducted safety evaluations of the anti‐GAL‐9 antibody in C57 mice (1 mg, administered three times weekly for 2 weeks) of cells exhibiting high GAL‐9 expression (e.g., endothelial cells, B cells, T cells). Results showed no significant difference in B cells between groups (Figure S14A) nor in T cells (Figure S14B). Subsequently, we established a human umbilical vein endothelial cell (HUVEC) line to evaluate the effects of anti‐GAL‐9 on endothelial cells. HUVEC cells were treated with anti‐GAL‐9 antibody (10 μg/mL) and IgG antibody (10 μg/mL), respectively. Results showed no significant difference in apoptosis between the two groups at 24 h (Figure S14C) and 48 h (Figure S14D). Statistical analysis of proliferation revealed no significant difference between the two groups at either 24 or 48 h (Figure S14E). Through the aforementioned evaluation and analysis of the primary cells expressing GAL‐9 protein, we propose the anti‐GAL‐9 antibody to be well‐tolerated in mice under this model.

### 3.7 Skin eccDNA Triggers JAK1/2‐STAT1 Phosphorylation in Splenic GMP Cells to Produce GAL‐9^high^ Neutrophils

**FIGURE 7:**
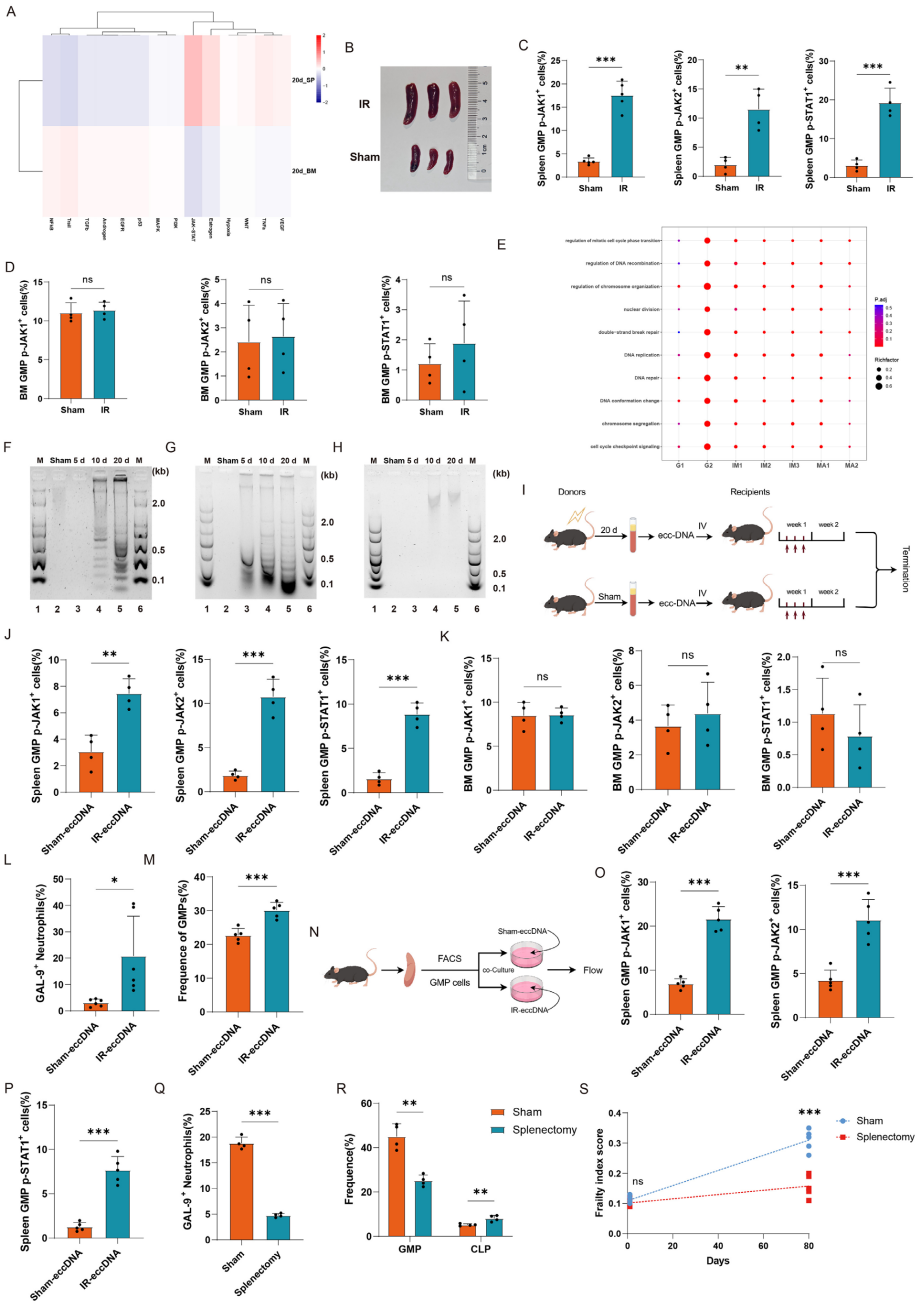
Skin eccDNA triggers JAK1/2‐STAT1 phosphorylation in splenic GMP cells to produce GAL‐9^high^ neutrophils. (A) Pathway enrichment of splenic and bone marrow neutrophils at 20 days post‐radiation by DecoupleR analysis. (B) Representative plots of spleen in the sham group and 20 days post‐radiation. (C, D) Phosphorylation levels of JAK1, JAK2, and STAT1 in (C) splenic and (D) bone marrow GMP cells in the sham group and 20 days post‐radiation. *n* = 4. (E) Pathway enrichment of DNA damage repair pathways in various populations of splenic neutrophils at 20 days post‐radiation by bubble plots. (F–H) Representative plots of eccDNA of (F) spleen, (G) skin, and (H) bone marrow in the sham group, 5, 10, and 20 days post‐radiation by agarose gel. (M), linear DNA marker. (I–M) Assessment of the dosing regimen for extracting different groups of circulating eccDNA for tail‐vein injection into recipients. (I) The scheme and phosphorylation levels of JAK1, JAK2, and STAT1 in (J) splenic and (K) bone marrow GMP cells, (L) circulating GAL‐9^high^ neutrophils, and (M) splenic GMP cells of recipients injected with Sham‐eccDNA or IR‐eccDNA. *n* = 4–6. (N–P) Assessment of the co‐culture of splenic GMP cells in the sham group with Sham‐eccDNA or IR‐eccDNA in vitro. (N) The scheme and phosphorylation levels of (O) JAK1, JAK2, and (P) STAT1 in splenic GMP cells under different treatment conditions. *n* = 5. (Q–S) Assessment of the effect of splenectomy and the sham group. (Q) Circulating GAL‐9^high^ neutrophils, (R) bone marrow GMP and CLP cells, and (S) Frailty index score after radiation in splenectomy and sham group. *n* = 4–5. Data are presented as mean ± SD; each dot represents an individual animal from at least 2–4 independent experiments that used male and female mice. ns, not significant, *p < 0.05, **p < 0.01, ***p < 0.001. Statistical analyses were performed using unpaired Student's *t*‐test (except R) and two‐way ANOVA (R).

GAL‐9^high^ neutrophils emerged as pivotal mediators of radiation‐induced frailty and multiple organ pathology. In search of better intervention strategies, we further explored the origin of these abnormally emerged neutrophils. The previous results indicated that GAL‐9^high^ neutrophils in the radiation group significantly elevated at 20 days post radiation (Figures 3N and 4A), therefore, we did DecoupleR analysis on the single‐cell results from bone marrow and spleen, the main neutrophil‐origin organs in this period, and the results suggested that at 20 days post radiation, the janus kinase‐signal transduction and transcription activation (JAK–STAT) pathway in the spleen was significantly activated in comparison to bone marrow (Figure 7A). The spleen was significantly enlarged in the radiation group (Figure 7B), which was consistent with the previous splenic weight abnormality results (Figure S2I). We subsequently transplanted splenic cells from the radiation and sham group into the spleen of recipient mice, and we found that splenic GMP cells (Figure S15A) and bone marrow GMP cells were elevated in the SPL‐IR group compared to the SPL‐sham group (Figure S15B). In combination with the strong extramedullary hematopoiesis in the spleen at 20 days post radiation (Figure 2J–O), we hypothesized that GAL‐9^high^ neutrophils might originate from the spleen rather than from the bone marrow. It has been reported by several studies that GAL‐9 is one of the immune molecules dependent on JAK1/2‐STAT1 phosphorylation (Park et al. 2011; Ye et al. 2023; Lv et al. 2017), and other studies also showed that TGFβ‐Smad3 pathway phosphorylation correlates with GAL‐9 production (Wu et al. 2014; Zhao et al. 2025; Abooali et al. 2025). Therefore, we measured the phosphorylation levels of the above molecules in splenic GMP cells from the sham group and 20 days post‐radiation. The phosphorylation levels of Smad3 indicated no significant difference between the two groups (Figure S15C). The phosphorylation levels of JAK1, JAK2, and STAT1 in splenic GMP cells of the radiation group were enhanced (Figure 7C and Figure S16A). We then examined the phosphorylation levels of the JAK1/2‐STAT1 in GMP cells in bone marrow, while there was no significant difference in the above indexes in bone marrow GMP cells (Figure 7D and Figure S16B). To further explore the substances that activate the spleen, we first examined the common activators that have been previously reported. Surprisingly, there was no increase in local IL‐2, IL‐4, IFN‐γ, and IL‐6 levels in the spleen (Figure S15D–G); so we speculated whether there was a new activator of the JAK1/2‐STAT1 pathway in the spleen following radiation. Then, we found that DNA damage and repair‐related pathways were significantly activated in various populations of splenic neutrophils compared with bone marrow at 20 days post radiation (Figure 7E). EccDNA, a ubiquitous chromosomal‐independent double‐stranded circular DNA in eukaryotes, exhibits amplified biogenesis following radiation‐induced DNA double‐strand breaks (DSBs) and triggers a strong DNA damage repair response (Yang, Su, et al. 2023; Li, Wang, Li, and Zhou 2022; Yang et al. 2022). So we speculated on the possibility of splenic activation by eccDNA after the local skin radiation. To verify the conjecture, we firstly examined the eccDNA content of spleen, and the results showed that a small amount of eccDNAs appeared in the spleen at 10 days after radiation, and the content increased markedly at 20 days (Figure 7F). Next, we examined the eccDNA content in the skin at different time after radiation, and the results showed that a small amount of eccDNA appeared in the skin at 5 days after radiation, and the content gradually increased at 10 and 20 days after radiation (Figure 7G), while no eccDNA was detected in the bone marrow (Figure 7H), which supported the conjecture that the spleen was activated by the eccDNA originated from the skin.

Further, we speculated that skin radiation‐induced eccDNA might undergo circulation trafficking to splenic reservoirs. To verify this conjecture, we extracted the circulating eccDNA of the sham and the radiation group for 20 days, and injected it into the recipient mice via the tail vein to evaluate the activation of recipient spleen (Figure 7I). The results showed that the phosphorylation levels of JAK1, JAK2, and STAT1 proteins in splenic GMP cells (Figure 7J and Figure S16C), the circulating GAL‐9^high^ neutrophils (Figure 7L and Figure S16E), and splenic GMP cells (Figure 7M and Figure S16F) were elevated in mice injected with circulating eccDNA in the radiation group compared with the control. There was no significant difference in the phosphorylation levels of JAK1, JAK2, and STAT1 proteins in bone marrow GMP cells (Figure 7K and Figure S16D). To further exclude the interference of other factors, an in vitro experiment was applied by incubating the sorted mouse splenic GMP cells with eccDNA from the sham and radiation groups, respectively (Figure 7N), to further clarify the relationship between the two. The results showed that the phosphorylation levels of JAK1, JAK2, and STAT1 proteins in splenic GMP cells could be activated by IR‐eccDNA (Figure 7O,P and Figure S16G). Finally, we irradiated the local skin of mice after splenectomy or sham with a single dose of 60 Gy, and the results showed that the splenectomy group had fewer GAL‐9^high^ neutrophils (Figure 7Q and Figure S16H) and bone marrow GMP cells compared with the sham group, while a certain degree of elevated CLP cells was observed (Figure 7R and Figure S16I,J). The bone marrow myeloid bias was partially reversed, and interestingly, the frailty index of the mice was significantly reduced after splenectomy (Figure 7S). In summary, we found that GAL‐9^high^ neutrophils derived from spleen rather than bone marrow, and that splenic GMP cells were induced by eccDNA from the skin, leading to activation of the JAK1/2‐STAT1 pathway.

## 4 Discussion

**FIGURE 8:**
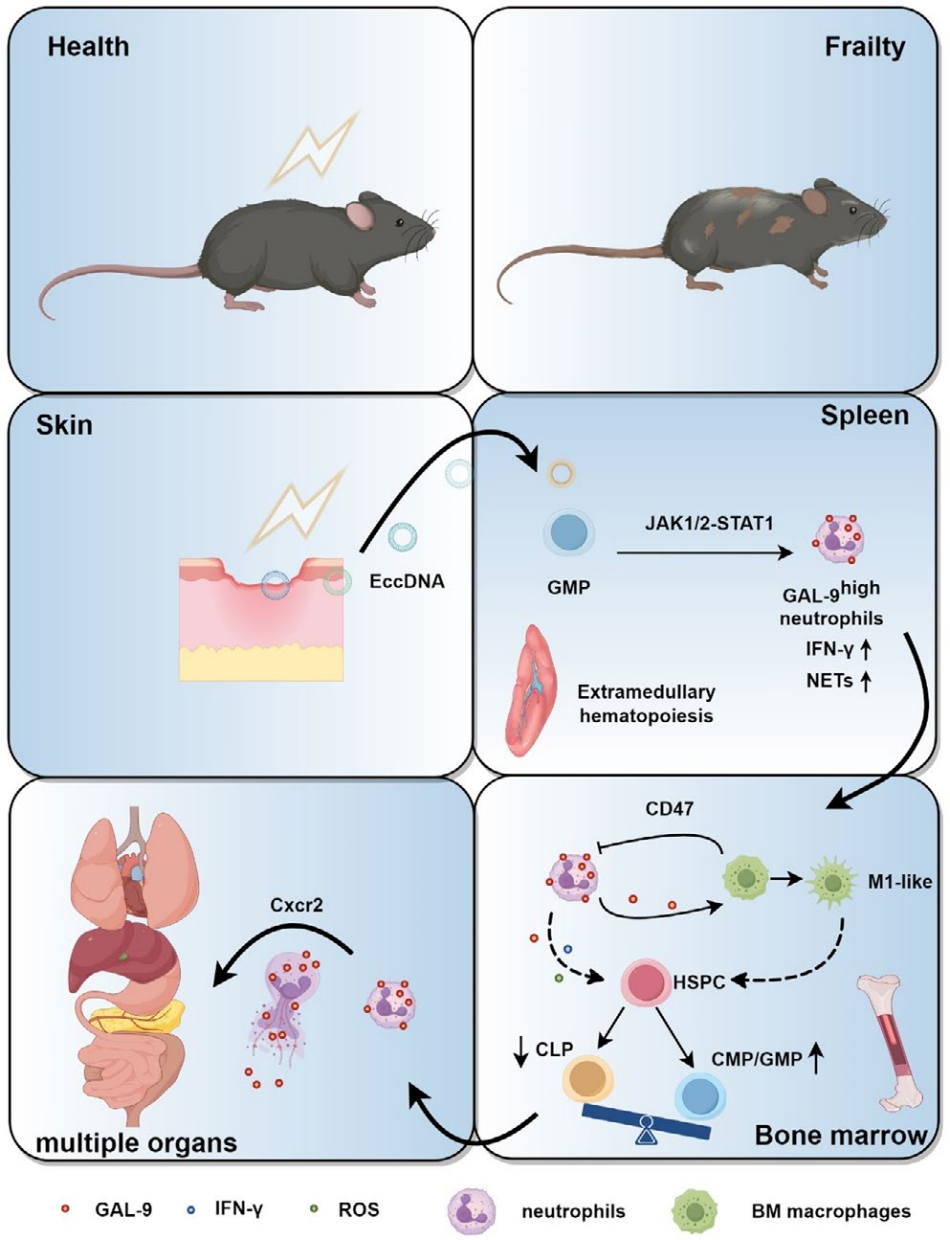
EccDNA shedding after skin radiation injury activates the JAK1/2‐STAT1 pathway in splenic GMP cells to induce the production of GAL‐9^high^ neutrophils. This unique population of hyperactive GAL‐9^high^ neutrophils is identified with characteristics of elevated NETs and IFN‐γ, etc. These neutrophils infiltrate into multiple organs to induce injuries, disrupt the bone marrow microenvironment, drive sustained bone marrow myeloid‐biased differentiation and polarization of bone marrow macrophages towards the M_1_ state, and resist clearance by bone marrow macrophages. Highlight the ‘skin‐spleen‐bone marrow‐multiple organs’ axis drives the generation of GAL‐9^high^ neutrophils to exacerbate frailty.

Although there is growing evidence that local radiation injury, such as in radiotherapy‐induced or nuclear accident casualties, can induce damage to distal organs (Piffkó et al. 2025), many childhood cancer survivors receiving radiotherapy suffer serious consequences in adulthood, including muscle loss (Ness et al. 2015; Kallenbach et al. 2022), premature aging (Rossi et al. 2021), and vital organ failure. This damage is more prevalent in the elderly, (O'Donovan et al. 2017; Güzelöz and Gök 2023; Kerstens et al. 2023; Eriksen et al. 2023; Wang, Zhen, et al. 2023) and these clinical manifestations, or what is called frailty, result in significant medical and societal burden. However, the underlying mechanisms and key regulatory factors remain unknown. Through the establishment of a local skin radiation injury murine model with frailty to investigate the underlying mechanisms, we applied single‐cell sequencing and drew the conclusion that a population of GAL‐9^high^ neutrophils characterized by hyperactivation emerged after radiation. This group of neutrophils could express high levels of NETs and IFN‐γ, which infiltrated into multiple organs and induced injuries and frailty. Although the formation of NETs was a key antimicrobial defense strategy, uncontrolled NETs formation led to sustained activation of the inflammatory cascade response and the recruitment of excess immune cells through the release of damage‐associated molecular patterns (DAMPs), including DNA‐containing ones (Cho et al. 2023), which posed a threat to highly vascularized tissues, such as the lungs (Du et al. 2024; Adrover et al. 2020). In addition, IFN‐γ could induce vascular endothelial cell injury (Xu et al. 2018). We also found that GAL‐9^high^ neutrophils could resist clearance by bone marrow macrophages, which resulted in the persistence of this highly aggressive population of neutrophils and led to disruption of the bone marrow microenvironment. Therefore, we suggested that GAL‐9^high^ neutrophils induced frailty through the mechanisms described above. Then we found that GAL‐9 protein was an important regulatory molecule in neutrophils' hyperactivity. Moreover, we discovered that eccDNA from the skin initiated the phosphorylation of the JAK1/2‐STAT1 in splenic GMP cells, which resulted in the generation of the GAL‐9^high^ neutrophils. Our study thus unravels the important role of a ‘skin‐spleen‐bone marrow‐multiple organs’ axis, offering new insights into the occurrence of frailty after local radiation injury and potential approaches (Figure 8).

Radiation alters cellular states by inducing DNA damage, leading to cell necrosis, apoptosis, or senescence, which accelerates cell senescence in tissues such as pulmonary fibroblasts and type II airway epithelial cells, subsequently promoting radiation‐induced pulmonary fibrosis (RIPF) (Su et al. 2021). Fibroblasts constitute a primary subpopulation of radiation‐induced skin senescence cells, and IL‐33 represents one of the most highly expressed cytokines in senescent fibroblasts, playing a crucial role in early radiation ulcer healing (Chen et al. 2024). Research indicates that tumor radiotherapy is associated with radiation‐induced bone damage and cellular senescence (Silwal et al. 2023). Radiation diminishes the viability of primary osteoblasts from the femur and tibia, alters functional protein expression, induces upregulation of NF‐κB ligands and osteonectin, and disrupts bone remodeling equilibrium, leading to osteoporosis and senescence (Wang, Xu, et al. 2021). Radiation correlates with age‐related intervertebral disc degeneration, though underlying mechanisms require further investigation (Zhong et al. 2022). Research has elucidated the molecular pathway whereby the Ca^2+^‐NFATc1‐Fos1 signaling axis mediates radiation‐induced osteodysplasia in mesenchymal stem cells by regulating mitochondrial dynamics, offering potential clinical strategies to mitigate radiation‐induced bone loss and aging (Ren et al. 2025). Recent studies confirm that radiation‐induced excessive ROS production in neurons directly attacks mitochondria, leading to mitochondrial DNA leakage into the cytoplasm and triggering sustained release of pro‐inflammatory factors by microglia. This reveals the key pathogenic mechanism of the ‘mitochondria‐immune axis’ in radiation‐induced brain injury (Shang et al. 2025).

Galectins are a class of animal lectins named for their high affinity for β‐galactosides (Rodrigues et al. 2024). GAL‐9 is involved in apoptosis and aggregation and is abundantly expressed in many cell types, possessing the ability to regulate cellular functions, such as in endothelial cells, (Iqbal et al. 2022) B cells, (Cao et al. 2018) and T cells, (Wu et al. 2014) but the relationship between its expression and neutrophil function is not well understood. In this study, we identified GAL‐9^high^ neutrophils in the circulation after local radiation for the first time. These neutrophils are hyperactive and characterized by a high level of NETs, IFN‐γ, ROS, etc. They also have significantly enhanced phagocytosis and life span and were a crucial mediator for frailty being induced (Adrover et al. 2019; Bukong et al. 2018; Grégoire et al. 2018). We found that GAL‐9^high^ neutrophils expressed a high level of IFN‐γ; this observation aligned with recent findings (Gour et al. 2024; Gomez et al. 2015; Yamada et al. 2011), a phenomenon requiring further investigation to elucidate its biological significance. Interestingly, we found that both surface and intracellular GAL‐9 protein expression in neutrophils were elevated after local radiation. Considering that the circulating GAL‐9 protein level was also elevated, we speculated that neutrophils might release GAL‐9 protein during functioning processes such as NETs formation. Previous studies have reported that GAL‐9 protein was elevated in the peripheral circulation in various disease models, such as *Francisella novicida* ‐infected murine model of sepsis, (Vega‐Carrascal et al. 2014) patients with rheumatoid arthritis, (Wiersma et al. 2019) and COVID‐19 infection (Bozorgmehr et al. 2021). Furthermore, a broad range of epidemiologic evidence pointed to a positive correlation between the level of circulating GAL‐9 protein in patients and the onset and progression of ANCA‐associated vasculitis, (Yoon et al. 2022) hepatic fibrosis, (Fujita et al. 2018) type 2 diabetes mellitus and chronic kidney disease, (Kurose et al. 2013) rheumatoid arthritis, (Vilar et al. 2019) and COVID‐19, (Bozorgmehr et al. 2021) among other diseases. Here, we revealed GAL‐9 protein played an important role in the hyperactivity of neutrophils.

Tumor‐associated neutrophils (TANs) are recognized as a collection of neutrophil states co‐shaped by the tumor microenvironment and systemic factors (Hedrick and Malanchi 2022). TANs/PMN‐MDSCs frequently exhibit ‘prolonged lifespan/apoptosis resistance’ within tumors (Cerezo‐Wallis et al. 2026). The GAL‐9^high^ neutrophils similarly demonstrate extended lifespan and persistent multiple organs infiltration, suggesting that ‘prolonged lifespan’ may represent a key common characteristic of these pathogenic neutrophil states (Ng et al. 2024; Gungabeesoon et al. 2023). The expansion of PMN‐MDSC/TANs in tumors is frequently associated with myeloid‐biased differentiation and bone marrow emergency granulopoiesis (Jaillon et al. 2020; Ghosh et al. 2023; Giese et al. 2019). The GAL‐9^high^ neutrophils can disrupt the bone marrow microenvironment and drive sustained myeloid‐biased differentiation. The classic pathogenic phenotype of TANs/PMN‐MDSCs involves suppressing T cells, promoting tumor immune evasion, and fostering treatment resistance (Raskov et al. 2022; Wang, Zheng, et al. 2023). The GAL‐9^high^ neutrophils primarily follow a ‘multiple organs infiltration‐tissue injury‐frailty’ pattern. Another type of neutrophil, low‐density neutrophils (LDNs), is typically defined by density gradient separation and is associated with excessive inflammation and immune imbalance in various diseases. It shows significant similarity to the GAL‐9^high^ neutrophil high ROS/NETs tendency, though LDNs are usually obtained via density gradient centrifugation, whereas GAL‐9^high^ neutrophils are characterized by high GAL‐9 expression (Blanco‐Camarillo et al. 2021; Fu, Wen, and Fan 2025).

Distal bone marrow damage and sustained myeloid‐biased differentiation after local radiation injury might be a critical part of the induction of frailty. The biased differentiation of bone marrow HSPCs towards myeloid cells is a crucial signal for the peripheral stress response, enabling the rapid generation of intrinsic immune cells like neutrophils and macrophages to perform their functions (Chavakis et al. 2019; Li, Wang, Yu, et al. 2022). However, sustained myeloid mobilization intensifies the burden on the bone marrow to the point of depletion (Chavakis et al. 2019). Neutrophils homing to the bone marrow under the aging condition (Casanova‐Acebes et al. 2013) have attracted attention as a bridge between peripheral organs and the bone marrow to achieve crosstalk. Additionally, Sreejit G et al. noted that during acute myocardial infarction, neutrophils deliver IL‐1β to the bone marrow, thereby inducing myelopoiesis (Sreejit et al. 2022). In our model, GAL‐9^high^ neutrophils homing to the bone marrow disrupted the microenvironment by releasing NETs, MPO, etc., and induced myeloid‐biased differentiation of HSPCs by releasing inflammatory factors such as IFN‐γ, ROS, etc., which were key factors that induced myeloid‐biased differentiation of the bone marrow (Chavakis et al. 2019). Bone marrow macrophages can secrete a series of anti‐inflammatory factors during efferocytosis of neutrophils and are converted from a pro‐inflammatory to an anti‐inflammatory state, preventing further expansion of inflammation (Bukong et al. 2018; Grégoire et al. 2018; Casanova‐Acebes et al. 2013; Li et al. 2023). However, we found that the GAL‐9 protein could induce polarization of bone marrow macrophages in a pro‐inflammatory manner, (Li et al. 2024) and GAL‐9^high^ neutrophils inhibited macrophage efferocytosis, reduced clearance of neutrophils themselves by elevating the expression of don't eat me signaling, and amplifying damage to the bone marrow. In the early stage following local skin radiation, particularly after the formation of a radiation ulcer, HSPCs underwent myeloid‐biased differentiation in response to local chemokines and recruitment factors that tended to the clearance of bacteria, pathogens, dead or aging cells. But we found that the bone marrow developed a time and dose‐dependent exacerbation of the myeloid‐biased state, which markedly aggravated the burden on the HSPCs and worsened the damage to the bone marrow. In addition, HSPCs are situated at the top of the hematopoietic hierarchy, undergoing profound functional decline during aging or injury, characterized by uncontrolled differentiation propensity, clonal hematopoiesis, myeloid differentiation bias, and diminished long‐term reconstitution capacity. These alterations drive immune system dysfunction and propagate systemic multiple organ injuries, (Yousefzadeh et al. 2021; Zhang et al. 2023) potentially constituting a novel contributing factor to frailty pathogenesis.

Our study revealed that the spleen was an important transit point connecting the skin and the bone marrow after local skin radiation, and was widely recognized as a major extramedullary hematopoietic organ, (Yang, Chen, et al. 2020; Mende et al. 2022; Liu et al. 2022) that recruited bone marrow HSPCs as an independent ecological niche corresponding to peripheral stress (Wu et al. 2018). In physiological conditions, less than 1% of HSPCs are present in the circulation for patrolling (Mende et al. 2022). However, the proportion significantly increases after the occurrence of a stress response. We found a significant increase in splenic weight 20 days after local skin radiation and a significant elevation of circulating LSK cells, coinciding with the time after the formation of radiation ulcers on the skin, suggesting the initiation of potent EMH by the spleen. Interestingly, the JAK1/2‐STAT1 pathway was markedly activated in the spleen, while previous reports of activation mediators such as IL‐2, IL‐6, and IFN‐γ were not significantly elevated in the spleen (Xue et al. 2023). However, considering the universal activation of DNA damage repair pathways across neutrophil subsets in the spleen, combined with the established role of radiation‐induced DNA double‐strand breaks in triggering robust DNA damage responses that generated abundant eccDNA, (Yang, Su, et al. 2023; Li, Wang, Li, and Zhou 2022; Yang et al. 2022) we proposed eccDNA as a candidate mediating neutrophil activation under these conditions. EccDNA is an aggregate of extrachromosomal circular DNAs of different fragment sizes that can activate multiple immune‐related signals, such as cGAS‐STING (Mackenzie et al. 2017; de Oliveira Mann and Kranzusch 2017). It is now broadly studied in tumors, whereas ecDNA in tumors refers to eccDNA whose fragments are often larger and might contain the complete gene sequences that can promote tumor metastasis and progression (Yang et al. 2022; Hung et al. 2024; Bailey et al. 2024). Nevertheless, the fragments of radiation‐associated eccDNA are mainly distributed in a few hundred bp. Fragments of eccDNA containing the complete gene were rarely reported, (Shi et al. 2025) thereby the elucidation of the functional identification, biological effects, and cellular origin of this part of eccDNA still needs further studies. Currently, the discussion on the biological functions of eccDNA is mainly focused on the nucleus, e.g., it can increase the copies of oncogenes (Shimizu 2021; Zhu et al. 2022; Turner et al. 2017). We found that it could be released into the circulation and regulate the functions of distal cells, (Shi et al. 2025) but the mechanism needs to be further explored. In addition, eccDNA is more stable due to its ring‐forming properties (Wang, Wang, et al. 2021) and has been observed in the plasma of pregnant women, (Sin et al. 2020) some tumor patients, (Kumar et al. 2017; Fu et al. 2024; Wu et al. 2022) and patients with systemic lupus erythematosus (Gerovska and Araúzo‐Bravo 2023). How eccDNA is recognized by cells and exerts its biological functions remains a major challenge in the field. Very recently, a paper demonstrated that the transmembrane protein CCDC25 functions as a NET‐DNA receptor on cancer cells (Yang, Liu, et al. 2020). This study further supports the existence of specific receptors for eccDNA on cell membranes and we will continue to identify the potential receptors to recognize eccDNA in the furture studies.

## Author Contributions

C.S., Z.C., L.M., and Y.C. conceived and designed the project. Z.C. performed the most experiments. Z.C., L.M., and Y.C. analyzed the data and drafted the manuscript; Y.D. edited the manuscript in English. Y.C., J.L., Y.L., W.X., and T.X. took part in animal experiments. L.M., W.H., J.Z., J.W., and L.C. took part in cell in vitro experiments. L.M. performed eccDNA‐related experiments. Y.C. took part in analyzing the data from single‐cell transcriptome sequencing. C.S., Z.C., and L.M. edited the manuscript. C.S. designed the study, supervised the experiments, and revised the manuscript. All authors have read and approved the final manuscript.

## Funding

This work was supported by National Natural Science Foundation of China, 82030056 and 82192884.

## Disclosure

The authors have nothing to report.

## Ethics Statement

Sex matched C57/BL6J mice (8–12 weeks of age) were obtained and followed the care and use of guidelines from Laboratory Animals of the AMU. The AMU Animal Care and Use Committee approved all experimental animal procedures (AMEWEC20230024).

## Conflicts of Interest

The authors declare no conflicts of interest.

## Supporting information


**Figure S1:** Radiation‐induced adipose loss and osteoporosis in mice. (A) Scheme of murine local skin radiation model, the bright part was the radiation field, where the mice's dorsal skin was exposed with a skeleton frame, and the remainder of the body was covered with a lead block. (B, C) Representative plots and statistical analysis of HE staining of subepidermis thickness at 80 day post‐radiation and the sham group. *n* = 10. (D) Adipose volume assessment at 80 day post‐radiation and sham group. *n* = 10. (E) Cataract incidence assessment in the radiation and sham group. *n* = 10. (F, G) The different groups of mouse femurs were observed by micro‐CT analysis at 80 days post‐radiation and the sham group. (F) Representative plots of femur micro‐CT scanning results and (G) statistical analysis of specific parameters at 80 days post‐radiation and sham group. *n* = 7. Data are presented as mean ± SD; each dot represents an individual animal from at least 2–4 independent experiments that used male and female mice. *p < 0.05, **p < 0.01, ***p < 0.001. Statistical analyses were performed using an unpaired Student's *t*‐test.
**Figure S2:** Radiation‐induced multiple organ injuries and senescence in mice. (A–E) *Cdkn1a* mRNA expression relative to *Actin* mRNA housekeeping gene in the (A) heart, (B) kidney, (C) liver, (D) lung, and (E) spleen at 0 day, 40 days, 60 days, and 80 days post‐radiation. *n* = 6. (F, G) Representative plots and statistics of P16 expression in multi‐organs by immunofluorescence at 80 days post‐radiation and sham group. *n* = 4. (H) Representative plots of HE staining of heart, kidney, liver, lung, and spleen at 80 days post‐radiation and the sham group. (I, J) Representative plots and statistics of oil red O staining of the liver of mice at 80 days post‐radiation and the sham group. *n* = 3. (K–L) Spleen weight/body weight and heart weight/body weight in the local radiation and sham group. *n* = 4–6. (M–P) Circulating serum (M) TG, (N) TP, (O) ALP, and (P) TC assays at 80 days post‐radiation and the sham group. *n* = 5. Data are presented as mean ± SD; each dot represents an individual animal from at least 2–3 independent experiments that used male and female mice. Data were analyzed by *t*‐test or one‐way ANOVA followed by post hoc test. ns, not significant, *p < 0.05, **p < 0.01, ***p < 0.001. Statistical analyses were performed using one‐way ANOVA (K) and unpaired Student's *t*‐test (A–E, G, J, and L–P).
**Figure S3:** Detection of *Cdkn2a* and *Cdkn1a* levels in the sham group, the local radiation group, and the skin trauma group. (A–E) Assessment of the expression of *Cdkn2a* and *Cdkn1a* levels in the sham, local radiation, and skin trauma groups. (A) The Scheme and *Cdkn2a* and *Cdkn1a* mRNA expression in muti‐organs at (B, C) 10 days and (D, E) 30 days post‐treatment in different groups. *n* = 6. Data are presented as mean ± SD; each dot represents an individual animal from at least 2 independent experiments that used male and female mice. ns, not significant, *p < 0.05, **p < 0.01, ***p < 0.001. Statistical analyses were performed using one‐way ANOVA.
**Figure S4:** Alterations in circulating and splenic immune cells after local skin radiation in mice. (A–D) Assessment of circulating immune cells frequence at different time post‐radiation. Circulating (A) T cells (CD3^+^), (B) the CD4^+^ T cells/CD8^+^ T cells ratio, (C) Treg cells (CD3^+^ CD4^+^ CD25^+^ FoxP3^+^), and (D) macrophages (CD11b^+^ F4/80^+^) in the sham group and 80 days post‐radiation. *n* = 4–6. (E–I) Assessment of splenic immune cells frequence in the sham group and 20 days post‐radiation. Representative flow plots and frequence of splenic (E) macrophages (CD11b^+^ F4/80^+^), including (F) resident macrophages (CD11b^+^ F4/80^+^ Ly‐6C^−^ MHC‐II^−^) and circulating recruited macrophages (CD11b^+^ F4/80^+^ Ly‐6C^−^ MHC‐II^+^), and (G) T cells (CD3^+^), the (H) CD4^+^ T cells/CD8^+^ T cells ratio, and the (I) Treg cells (CD3^+^ CD4^+^ CD25^+^ FoxP3^+^). *n* = 5–6. (J–N) Assessment of LSK cells frequence and their cell cycle in the local radiation group and the sham group. Representative flow plots and frequence of (J) bone marrow CXCR4^+^ LSK cells (Lin^−^ Sca‐1^+^ c‐Kit^+^ CXCR4^+^), (K–N) bone marrow and splenic Ki67^+^ LSK cells (Lin^−^ Sca‐1^+^ C‐kit^+^ Ki67^+^) in the sham and radiation group. *n* = 4–7. Data are presented as mean ± SD; each dot represents an individual animal from at least 2–3 independent experiments that used male and female mice. ns, not significant, *p < 0.05, **p < 0.01, ***p < 0.001. Statistical analyses were performed using unpaired Student's *t*‐test (A–C, E, G–J, and M–N) and two‐way ANOVA (F).
**Figure S5:** Evaluation of distal bone marrow myeloid‐biased differentiation induced by different local skin radiation doses. (A–G) Assessment of distal bone marrow myeloid‐biased differentiation induced by different radiation doses. (A) The scheme and representative flow plots and frequence of bone marrow (B, C) MP cells, (D, E) CMP cells, GMP cells, MEP cells, and (F, G) CLP cells induced by different radiation doses. *n* = 3. Data are presented as mean ± SD; each dot represents an individual animal from at least 2 independent experiments that used male and female mice. ns, not significant, *p < 0.05, **p < 0.01, ***p < 0.001. Statistical analyses were performed using one‐way ANOVA (C, G) and two‐way ANOVA (E).
**Figure S6:** Single‐cell sequencing reveals the basic features of various populations of neutrophils. (A, B) UMAP and its cellular annotation of circulation, spleen, and bone marrow cells at different time in mice. (C, D) The pseudotime values and statistics of neutrophils for each population. (E) Heatmap of differentially expressed genes based on pseudotime values for each population of neutrophils. (F, G) Volcano and bubble plots of differentially expressed genes in each population of neutrophils. (H) Prediction of regulon values by Scenic analysis for each population of neutrophils. (I) Bubble plots of GO‐enriched pathways of differentially expressed genes in each population of neutrophils. (J, K) Splitting of neutrophils UMAP (Figure 3B) according to different sources.
**Figure S7:** Single‐cell sequencing compares differences in MA2 neutrophil subpopulations relative to other subpopulations. (A) MA2 neutrophil subpopulations relative to MA1 upregulated GO (CC) and KEGG pathways. (B–E) (B) Special granule score, (C) transcription factor score, (D) phagocytosis score, and (E) cell proliferation score for each population of neutrophils by violin plots. (F–G) Differential number/strength of interactions with each population of neutrophils in the local radiation group relative to the sham group by CellChat analysis. (H, I) ‘Incoming/Outgoing interantion strength’ and ‘Incoming signaling patterns’ of each population of neutrophils in the sham group and 80 days post‐radiation by CellChat analysis. (J) Prediction of MA2 neutrophil subpopulation Galectin pathway major ligand receptors by CellChat analysis. (K) Lgals9 gene expression in each population of neutrophils by violin plots. (L) Analysis of the major up‐ and down‐regulated pathways from the MA2 neutrophil subpopulation to other neutrophil subpopulations after radiation by CellChat.
**Figure S8:** Exclusion of SELPG and ANNEXIN pathways as markers for MA2 neutrophil subpopulations. (A–D) Characterization of the expression of the (A, B) SELPG and (C, D) ANNEXIN pathway and its major ligand receptors in the sham group and 80 days post‐radiation by CellChat analysis.
**Figure S9:** GAL‐9^high^ neutrophils are crucial mediators for inducing frailty after local radiation injury. (A) Representative flow plots of circulating GAL‐9^high^ neutrophils at different time post‐radiation. (B, C) Representative flow plots of (B) ROS and (C) IFN‐γ expression of circulating neutrophils after treatment in the sham group and 80 days post‐radiation. (D, E) Representative plots and statistics of MPO expression of neutrophils in multiple organs by immunofluorescence. *n* = 4. (F, G) Representative flow plots of bone marrow neutrophils (F) ROS and (G) IFN‐γ expression after treatment in the sham group and 80 days post‐radiation. (H) Representative plots of HE staining of bone marrow in the sham group and 80 days post‐radiation. (I–K) Representative flow plots and frequence of bone marrow (I, J) non‐immune cells (CD45^−^ Ter119^−^), (K) including endothelial cells, SECs, and stromal cells at different time post‐radiation. *n* = 3. Data are presented as mean ± SD; each dot represents an individual animal from at least 2–3 independent experiments that used male and female mice. ***p < 0.001. Statistical analyses were performed using unpaired Student's *t*‐test (E) and one‐way ANOVA (J).
**Figure S10:** Analysis of GAL‐9^high^ neutrophil chemotaxis and TCGA database analysis. (A) Analysis of the major up‐regulated pathways from other neutrophil subpopulations to the MA2 neutrophil subpopulation after radiation by CellChat. (B) CXCR2 expression of each population of neutrophils by violin plots. (C) CXCR2 expression of each group of neutrophils by violin plots. (D) Representative flow plots and frequency of circulating CXCR2^high^ neutrophils in the sham group and 80 days post‐radiation. *n* = 4. (E) Representative plots and statistics of CXCR2^+^ GAL‐9^+^ neutrophils in multi‐organs by immunofluorescence at 80 days post‐radiation and the sham group. *n* = 4. (F) Representative flow plots and frequency of circulating CXCR4^low^ neutrophils in the sham group and 80 days post‐radiation. *n* = 5. (G–J) K‐M curve analysis of GAL‐9^high^ neutrophils‐related genes in (G) ESCA, (H) LUSC, (I) PAAD, and (J) STAD in the TCGA database. *p*‐value was shown. Data are presented as mean ± SD; each dot represents an individual animal from at least 2–3 independent experiments that used male and female mice. ns, not significant, **p < 0.01, ***p < 0.001. Statistical analyses were performed using unpaired Student's *t*‐test (D–F) and the log‐rank test (G–J).
**Figure S11:** GAL‐9 protein induce neutrophils IFN‐γ expression. (A) Representative flow plots of intracellular GAL‐9 protein in circulating neutrophils in the sham group and 80 days post‐radiation. (B) Dose‐gradient experiments with exogenous supplementation of rmGAL‐9 protein to induce neutrophil IFN‐γ production. *n* = 3. (C) Representative flow plots of IFN‐γ expression of circulating neutrophils in the sham group after treatment with different conditions. (D–F) Representative flow plots of IFN‐γ expression of circulating neutrophils in the sham group by treatment with (D) circulating serum at 80 days post‐radiation, (E) culture supernatant of GAL‐9^high^ neutrophils, and (F) IFN‐γ expression of GAL‐9^high^ neutrophils 80 days post‐radiation under different stimuli. Data are presented as mean ± SD; each dot represents an individual animal from at least 2 independent experiments that used male and female mice.
**Figure S12:** GAL‐9 protein induce bone marrow macrophages pro‐inflammatory state. (A) Differential number/strength of interactions with bone marrow cells in the local radiation group relative to the sham group by CellChat analysis (red means higher, blue means lower, and the width of the line represents the interaction strength). (B) 80 days post‐radiation of bone marrow macrophages relative to the sham group majorly upregulated BP and the KEGG pathway. (C, D) Representative flow plots and frequence of bone marrow (C) macrophages and (D) their polarization state (M_1_‐like: CD11b^+^ F4/80^+^ CD11c^+^ CD206^−^, M_2_‐like: CD11b^+^ F4/80^+^ CD11c^−^ CD206^+^) at different time post‐radiation. *n* = 3. (E, F) (E) Inflammation‐related genes and (F) pro‐inflammatory resolution‐related genes expression in bone marrow macrophages in the sham group and 80 days post‐radiation. *n* = 3. (G) The main bone marrow cell interaction pathways by CellChat analysis for the sham group and 80 days post‐radiation. (H) The main Incoming signaling patterns of bone marrow cells in the sham group and 80 days post‐radiation by CellChat analysis. (I–K) Representative flow plots of the effect of (I) rmGAL‐9 protein, (J) circulating serum at 80 days post‐radiation, and (K) culture supernatant of GAL‐9^high^ neutrophils on the polarization of bone marrow macrophages. (L) Analysis of the major up‐ and down‐regulated pathways of bone marrow cells to neutrophils by CellChat analysis. (M) Bone marrow macrophages related genes expression in the sham group and 80 days post‐radiation. *n* = 6. Data are presented as mean ± SD; each dot represents an individual animal from at least 2–3 independent experiments that used male and female mice. ns, not significant, *p < 0.05, **p < 0.01, ***p < 0.001. Statistical analyses were performed using unpaired Student's *t*‐test (E, F, M), one‐way ANOVA (C), and two‐way ANOVA (D).
**Figure S13:** GAL‐9 protein intervention. (A–E) Representative flow plots of bone marrow (A) CMP cells, (B) CLP cells, (C) non‐immune cells, (D) macrophages, and (E) their polarization state after different treatments in local radiation and the sham group.
**Figure S14:** Safety analysis of anti‐GAL‐9 antibody. (A) Representative flow plots and frequency of circulating B cells in the IgG group and anti‐GAL‐9 antibody group. *n* = 4. (B) Representative flow plots and frequency of circulating T cells in the IgG group and anti‐GAL‐9 antibody group. *n* = 4. (C, D) Representative flow plots and frequency of apoptotic HUVEC cells in the IgG group and anti‐GAL‐9 antibody group after 24 h and 48 h. *n* = 4. (E) The cell counts of HUVEC cells in the IgG group and anti‐GAL‐9 antibody group analyzed by the CCK‐8 kit. *n* = 4. Data are presented as mean ± SD; each dot represents an individual animal from at least 2–3 independent experiments that used male and female mice. ns, not significant. Statistical analyses were performed using unpaired Student's *t*‐test.
**Figure S15:** Expression of common activation mediators of the splenic JAK1/2‐STAT1 pathway in the sham group and 20 days post‐radiation. (A, B) Representative flow plots and frequence of (A) splenic and (B) bone marrow GMP cells in SPL‐Sham or SPL‐IR. *n* = 3. (C) Representative flow plots and frequency of phosphorylation levels of Smad3 in splenic GMP cells from sham group and 20 days post‐radiation. *n* = 5. (D–G) Representative flow plots and frequence of splenic IL‐2, IL‐4, IL‐6, and IFN‐γ expression in the sham group and 20 days post‐radiation. *n* = 5–8. Data are presented as mean ± SD; each dot represents an individual animal from at least 2–3 independent experiments that used male and female mice. ns, not significant, *p < 0.05, **p < 0.01, ***p < 0.001. Statistical analyses were performed using an unpaired Student's *t*‐test (A–G).
**Figure S16:** Skin eccDNA triggers JAK1/2‐STAT1 pathway in splenic GMP cells to produce GAL‐9^high^ neutrophils. (A, B) Representative flow plots of phosphorylation levels of JAK1, JAK2, and STAT1 in (A) splenic and (B) bone marrow GMP cells in the sham group and 20 days post‐radiation. (C–F) Representative flow plots of phosphorylation levels of JAK1, JAK2, and STAT1 in (C) splenic and (D) bone marrow GMP cells, (E) circulating GAL‐9^high^ neutrophils, and (F) splenic GMP cells of recipients injected with Sham‐eccDNA or IR‐eccDNA. (G) Representative flow plots of phosphorylation levels of JAK1, JAK2, and STAT1 in splenic GMP cells under different treatment conditions. (H–J) Representative flow plots of (H) circulating GAL‐9^high^ neutrophils, bone marrow (I) GMP, and (J) CLP cells after radiation in the splenectomy and sham group.

**Video S1:** acel70448‐sup‐0002‐Video1.avi.

**Video S2:** acel70448‐sup‐0003‐Video2.avi.

**Video S3:** acel70448‐sup‐0004‐Video3.avi.

## Data Availability

The data that support the findings of this study are openly available in CNCB at https://www.cncb.ac.cn/, reference number PRJCA056484.
