# Supplementary material for: Galectin‐9high Neutrophils Exacerbate Radiation‐Induced Frailty
Source: Aging Cell. 2026 Apr 7;25(4):e70448. doi: 10.1111/acel.70448 (PMC13056513; doi:10.1111/acel.70448)
Supplement: Supplementary file 1 — Figure S1: Radiation‐induced adipose loss and osteoporosis in mice. (A) Scheme of murine local skin radiation model, the bright part was the radiation field, where the mice's dorsal skin was exposed with a skeleton frame, and the remainder of the body was covered with a lead block. (B, C) Representative plots and statistical analysis of HE staining of subepidermis thickness at 80 day post‐radiation and the sham group. n = 10. (D) Adipose volume assessment at 80 day post‐radiation and sham group. n = 10. (E) Cataract incidence assessment in the radiation and sham group. n = 10. (F, G) The different groups of mouse femurs were observed by micro‐CT analysis at 80 days post‐radiation and the sham group. (F) Representative plots of femur micro‐CT scanning results and (G) statistical analysis of specific parameters at 80 days post‐radiation and sham group. n = 7. Data are presented as mean ± SD; each dot represents an individual animal from at least 2–4 independent experiments that used male and female mice. *p < 0.05, **p < 0.01, ***p < 0.001. Statistical analyses were performed using an unpaired Student's t‐test. Figure S2: Radiation‐induced multiple organ injuries and senescence in mice. (A–E) Cdkn1a mRNA expression relative to Actin mRNA housekeeping gene in the (A) heart, (B) kidney, (C) liver, (D) lung, and (E) spleen at 0 day, 40 days, 60 days, and 80 days post‐radiation. n = 6. (F, G) Representative plots and statistics of P16 expression in multi‐organs by immunofluorescence at 80 days post‐radiation and sham group. n = 4. (H) Representative plots of HE staining of heart, kidney, liver, lung, and spleen at 80 days post‐radiation and the sham group. (I, J) Representative plots and statistics of oil red O staining of the liver of mice at 80 days post‐radiation and the sham group. n = 3. (K–L) Spleen weight/body weight and heart weight/body weight in the local radiation and sham group. n = 4–6. (M–P) Circulating serum (M) TG, (N) TP, (O) ALP, and (P) TC assays at 80 [file ACEL-25-e70448-s001.docx]

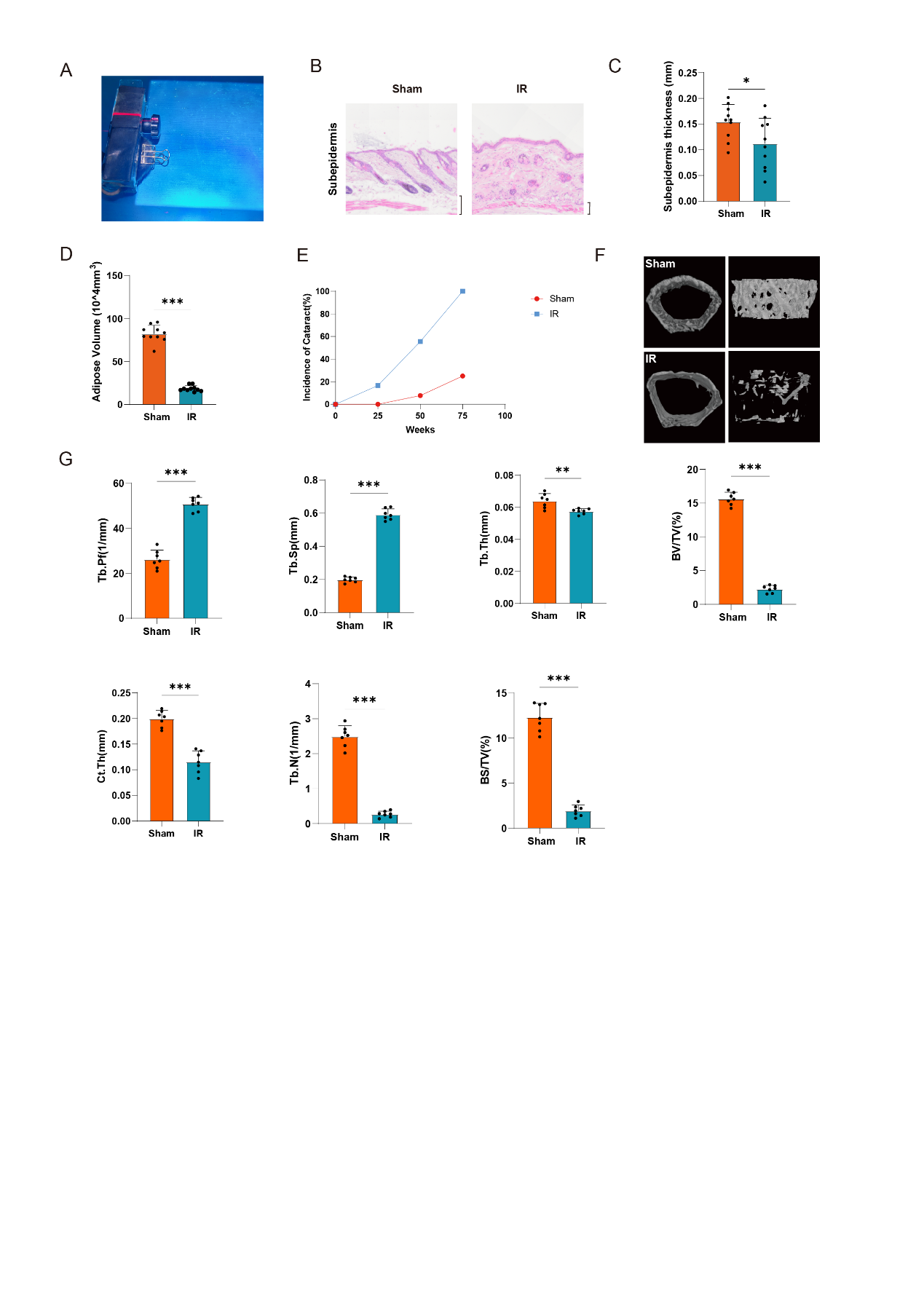


**Figure S1. Radiation-induced adipose loss and osteoporosis in mice.** (A) Scheme of murine local skin radiation model, the bright part was the radiation field, where the mice's dorsal skin was exposed with a skeleton frame, and the remainder of the body was covered with a lead block. (B-C) Representative plots and statistical analysis of HE staining of subepidermis thickness at 80d post-radiation and the sham group. n=10. (D) Adipose volume assessment at 80d post-radiation and sham group. n=10. (E) Cataract incidence assessment in the radiation and sham groups. n=10. (F-G) The different groups of mouse femurs were observed by micro-CT analysis at 80d post-radiation and the sham group. (F) Representative plots of femur micro-CT scanning results and (G) statistical analysis of specific parameters at 80d post-radiation and sham group. n=7. Data are presented as mean ± SD; each dot represents an individual animal from at least 2–4 independent experiments that used male and female mice. *p < 0.05, **p < 0.01,***p < 0.001. Statistical analyses were performed using an unpaired Student’s t-test.

**
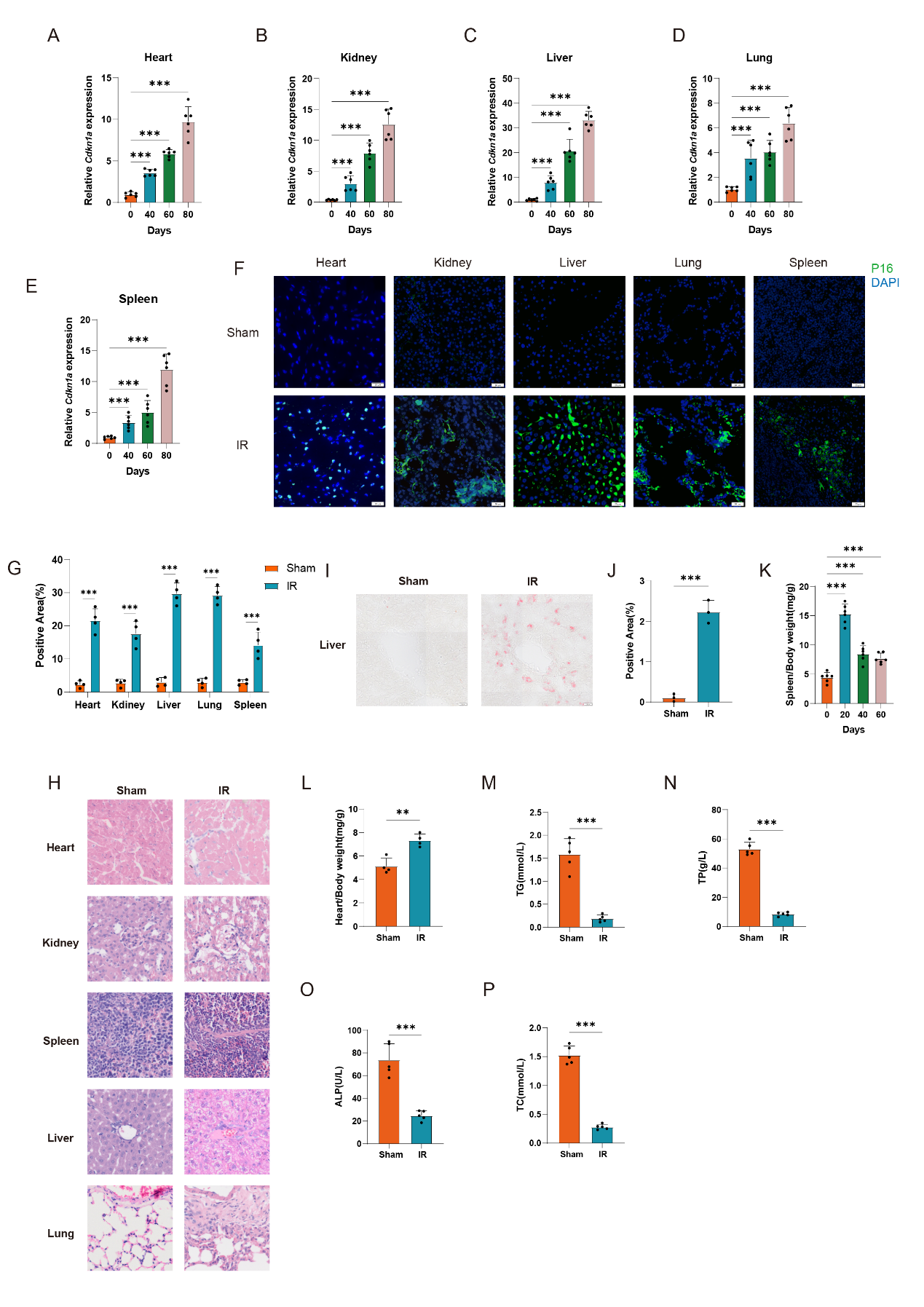
**

**Figure S2 Radiation-induced multiple organ injuries and senescence in mice.** (A-E) *Cdkn1a* mRNA expression relative to *Actin* mRNA housekeeping gene in the (A) heart, (B) kidney, (C) liver, (D) lung, and (E) spleen at 0d, 40d, 60d, and 80d post-radiation. n = 6. (F-G) Representative plots and statistics of P16 expression in multi-organs by immunofluorescence at 80d post-radiation and sham group. n = 4. (H) Representative plots of HE staining of heart, kidney, liver, lung, and spleen at 80d post-radiation and the sham group. (I-J) Representative plots and statistics of oil red O staining of the liver of mice at 80d post-radiation and the sham group. n = 3. (K-L) Spleen weight/body weight and heart weight/body weight in the local radiation and sham group. n = 4-6. (M-P) Circulating serum (M) TG, (N) TP, (O) ALP, and (P) TC assays at 80d post-radiation and the sham group. n = 5. Data are presented as mean ± SD; each dot represents an individual animal from at least 2–3 independent experiments that used male and female mice. Data were analyzed by t-test or one-way ANOVA followed by post hoc test. ns, not significant, *p < 0.05, **p < 0.01,***p < 0.001. Statistical analyses were performed using one-way ANOVA (K) and unpaired Student’s t test (A-E, G, J, and L-P).


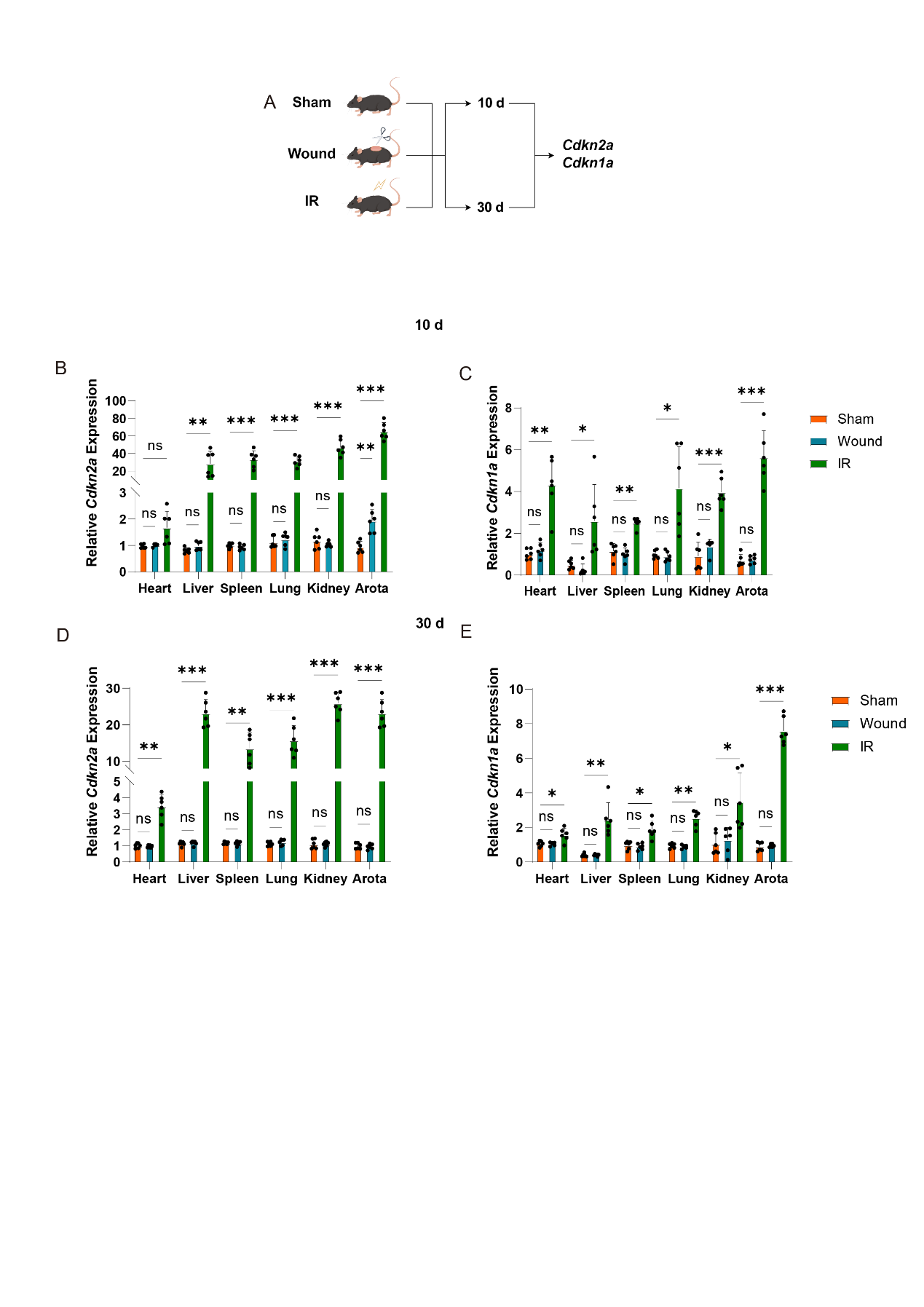


**Figure S3.** **Detection of *Cdkn2a* and *Cdkn1a* levels in** **the sham group, the local radiation group, and the skin trauma group.** (A-E) Assessment of the expression of *Cdkn2a* and *Cdkn1a* levels in the sham, local radiation, and skin trauma groups. (A) The Scheme and *Cdkn2a* and *Cdkn1a* mRNA expression in muti-organs at (B, C) 10d and (D, E) 30d post-treatment in different groups. n = 6. Data are presented as mean ± SD; each dot represents an individual animal from at least 2 independent experiments that used male and female mice. ns, not significant, *p < 0.05, **p < 0.01,***p < 0.001. Statistical analyses were performed using one-way ANOVA.


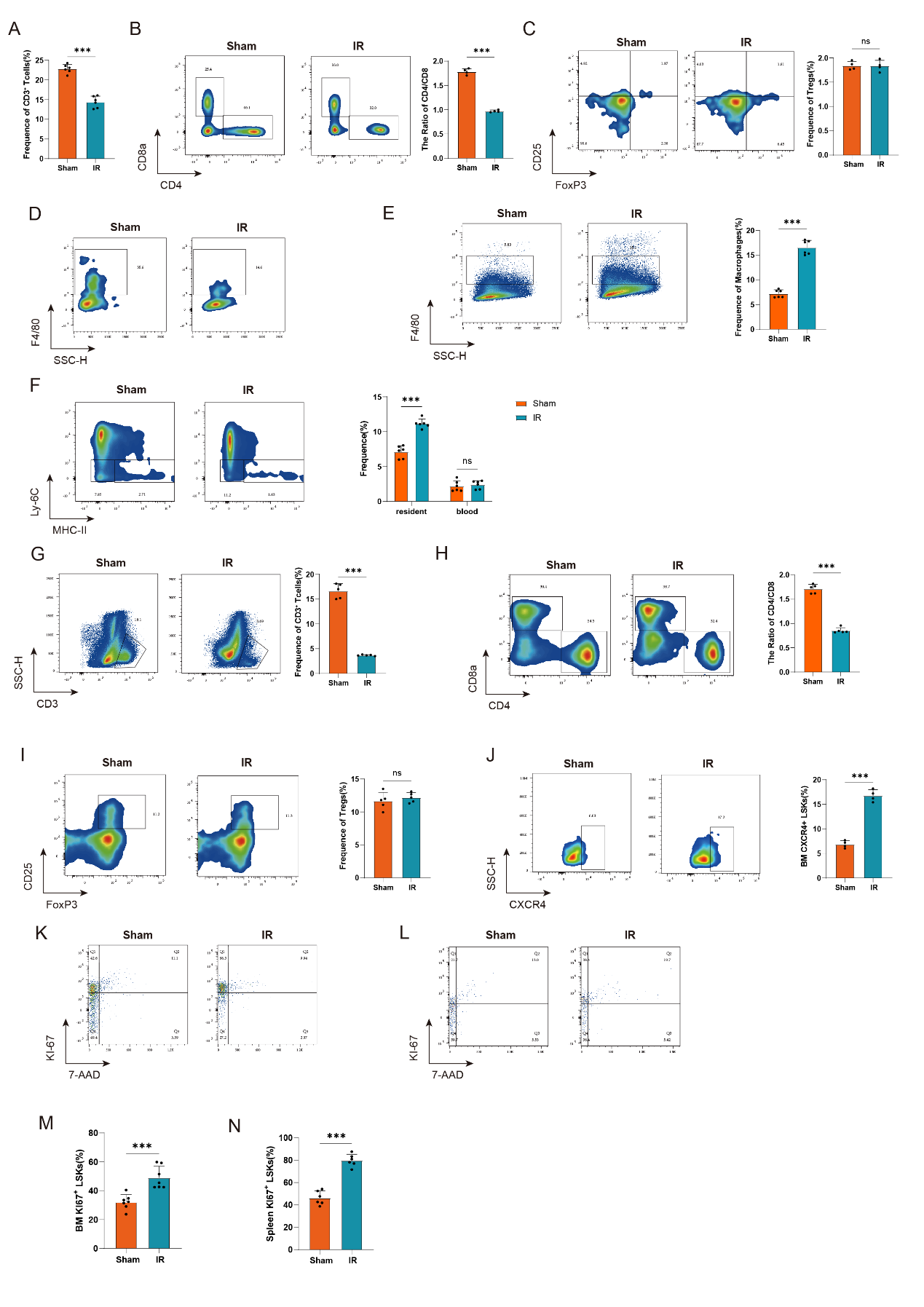


**Figure S4** **Alterations in circulating and splenic immune cells after local skin radiation in mice.** (A-D) Assessment of circulating immune cells frequence at different time points post-radiation. Circulating (A) T cells (CD3^+^), (B) the CD4^+^ T cells/CD8^+^ T cells ratio, (C) Treg cells (CD3^+^ CD4^+^ CD25^+^ FoxP3^+^), and (D) macrophages (CD11b^+^ F4/80^+^) in the sham group and 80d post-radiation. n= 4-6. (E-I) Assessment of splenic immune cells frequence in the sham group and 20d post-radiation. Representative flow plots and frequence of splenic (E) macrophages (CD11b^+^ F4/80^+^), including (F) resident macrophages (CD11b^+^ F4/80^+^ Ly-6C^-^ MHC-Ⅱ^-^) and circulating recruited macrophages (CD11b^+^ F4/80^+^ Ly-6C^-^ MHC-II^+^), and (G) T cells (CD3^+^), the (H) CD4^+^ T cells/CD8^+^ T cells ratio, and the (I) Treg cells (CD3^+^ CD4^+^ CD25^+^ FoxP3^+^). n = 5-6. (J-N) Assessment of LSK cells frequence and their cell cycle in the local radiation group and the sham group. Representative flow plots and frequence of (J) bone marrow CXCR4^+^ LSK cells (Lin^-^ Sca-1^+^ c-Kit^+^ CXCR4^+^), (K-N) bone marrow and splenic Ki67^+^ LSK cells（Lin^-^ Sca-1^+^ C-kit^+^ Ki67^+^）in the sham and radiation group. n = 4-7. Data are presented as mean ± SD; each dot represents an individual animal from at least 2–3 independent experiments that used male and female mice. ns, not significant, *p < 0.05, **p < 0.01,***p < 0.001. Statistical analyses were performed using unpaired Student’s t test (A-C, E, G-J, and M-N) and two-way ANOVA (F).


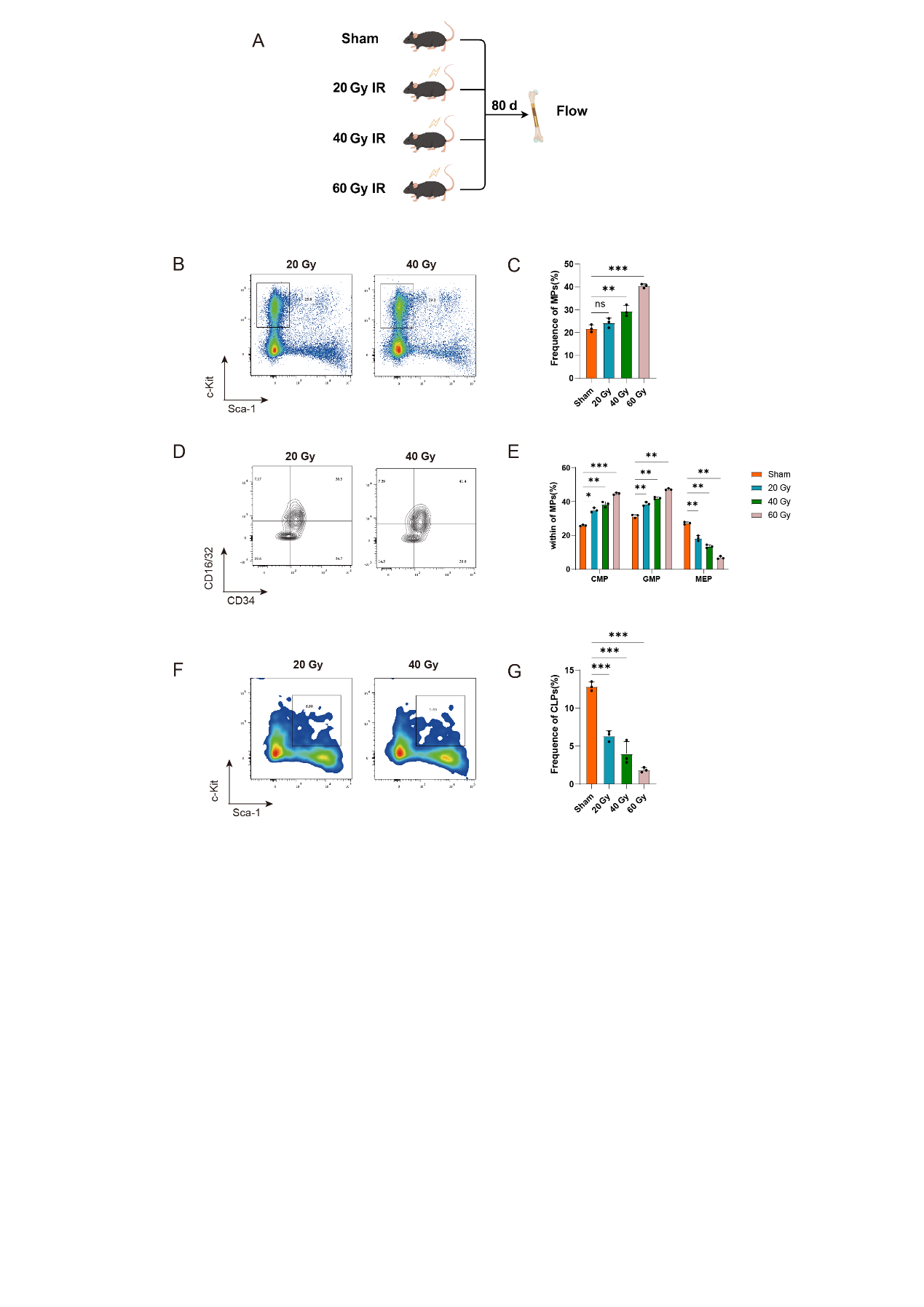


**Figure S5. Evaluation of distal bone marrow myeloid-biased differentiation induced by different local skin radiation doses.** (A-G) Assessment of distal bone marrow myeloid-biased differentiation induced by different radiation doses. (A) The scheme and representative flow plots and and frequence of bone marrow (B-C) MP cells, (D-E) CMP cells, GMP cells, MEP cells, and (F-G) CLP cells induced by different radiation doses. n =3. Data are presented as mean ± SD; each dot represents an individual animal from at least 2 independent experiments that used male and female mice. ns, not significant, *p < 0.05, **p < 0.01,***p < 0.001. Statistical analyses were performed using one-way ANOVA (C, G) and two-way ANOVA (E).
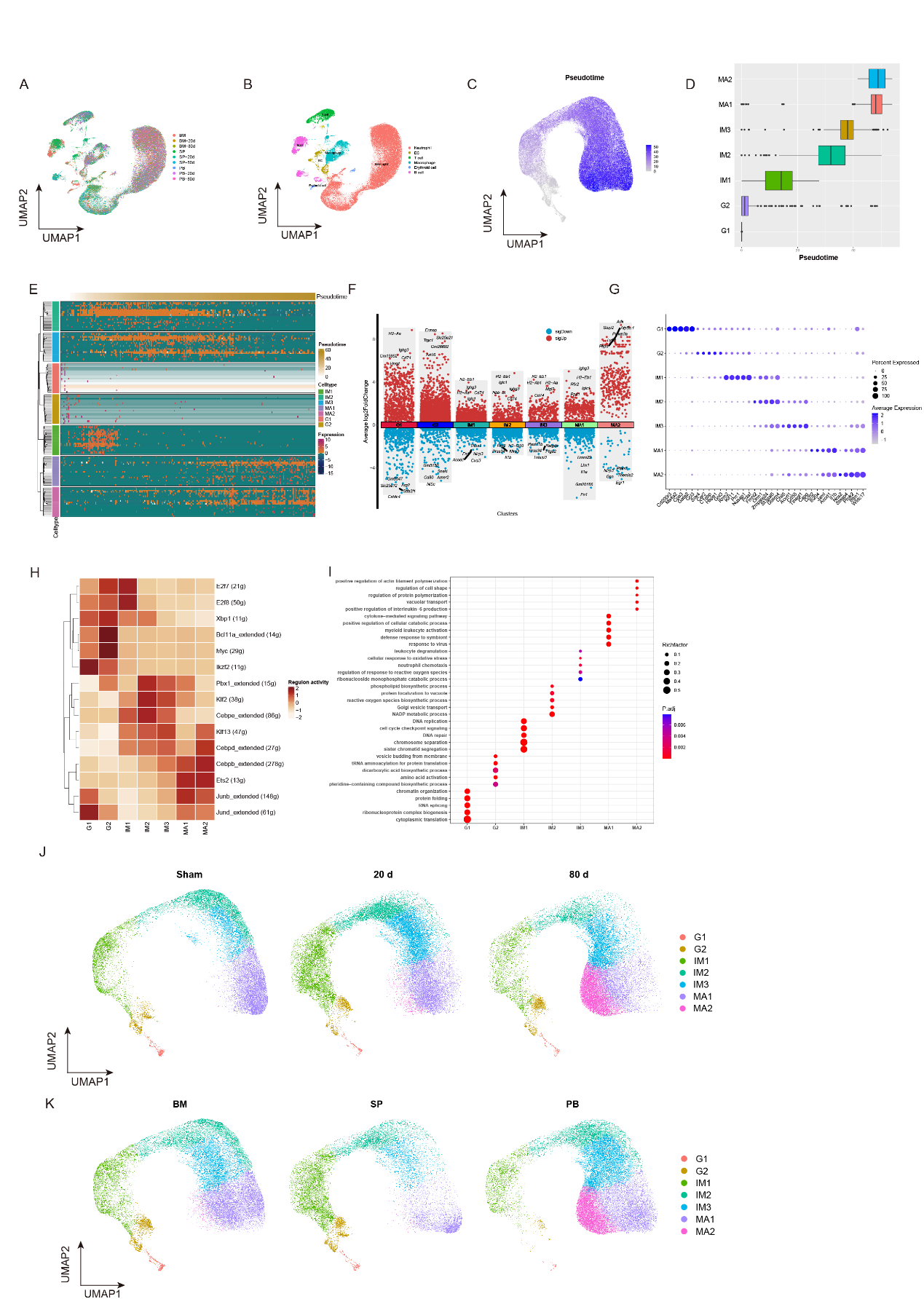


**Figure S6. Single-cell sequencing reveals the basic features of various populations of neutrophils.** (A-B) UMAP and its cellular annotation of circulation, spleen, and bone marrow cells at different time points in mice. (C-D) The pseudotime values and statistics of neutrophils for each population. (E) Heatmap of differentially expressed genes based on pseudotime values for each population of neutrophils. (F-G) Volcano and bubble plots of differentially expressed genes in each population of neutrophils. (H) Prediction of regulon values by Scenic analysis for each population of neutrophils. (I) Bubble plots of GO-enriched pathways of differentially expressed genes in each population of neutrophils. (J-K) Splitting of neutrophils UMAP (Fig. 2B) according to different sources.


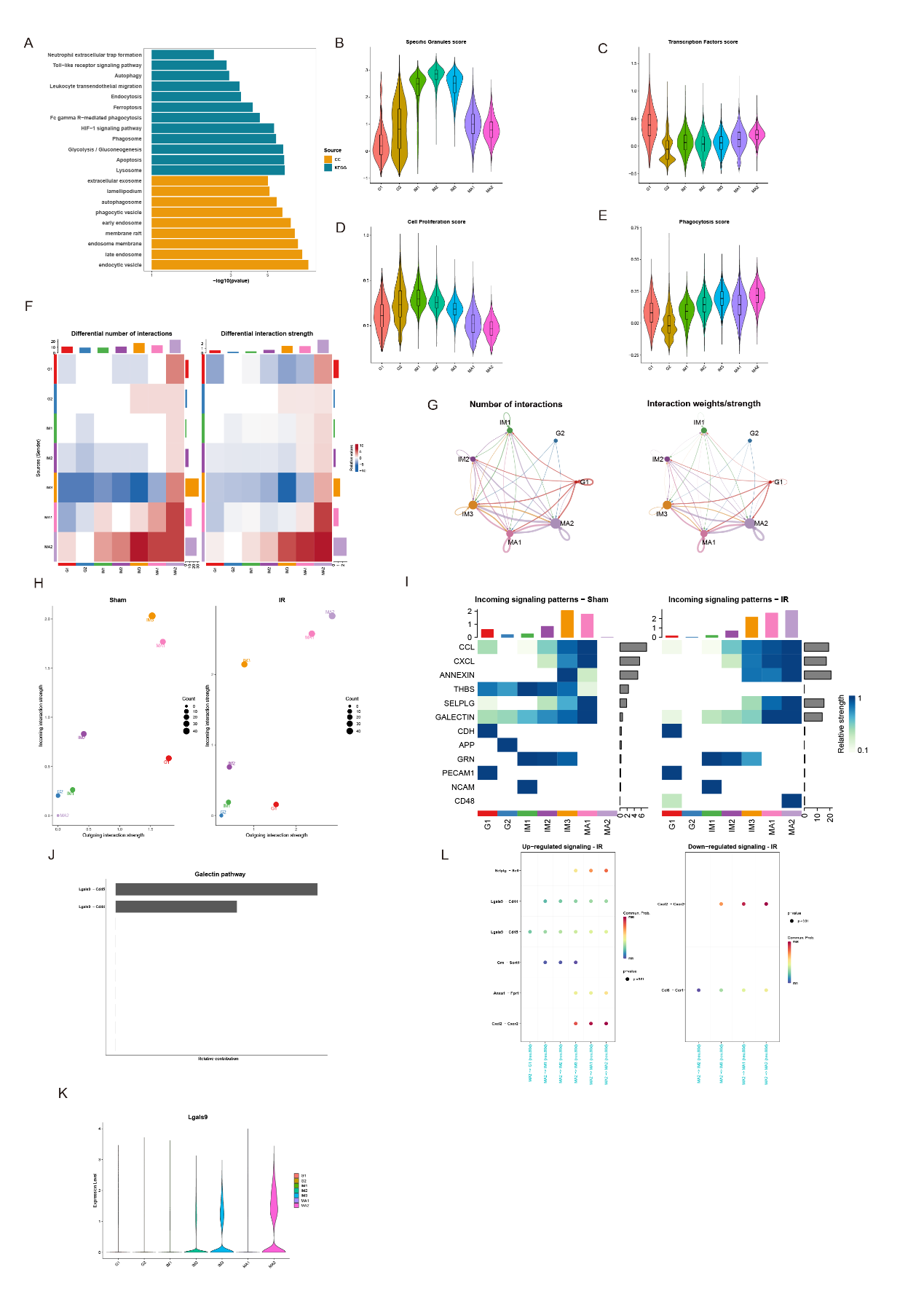


**Figure S7. Single-cell sequencing compares differences in MA2 neutrophil subpopulations relative to other subpopulations.** (A) MA2 neutrophil subpopulations relative to MA1 upregulated GO (CC) and KEGG pathways. B-E) (B) Special granule score, (C) transcription factor score, (D) phagocytosis score, and (E) cell proliferation score for each population of neutrophils by violin plots. (F-G) Differential number/strength of interactions with each population of neutrophils in the local radiation group relative to the sham group by CellChat analysis. (H-I) ‘Incoming/Outgoing interantion strength’ and ‘Incoming signaling patterns’ of each population of neutrophils in the sham group and 80d post-radiation by CellChat analysis. (J) Prediction of MA2 neutrophil subpopulation Galectin pathway major ligand receptors by CellChat analysis. (K) Lgals9 gene expression in each population of neutrophils by violin plots. (L) Analysis of the major up- and down-regulated pathways from the MA2 neutrophil subpopulation to other neutrophil subpopulations after radiation by CellChat.


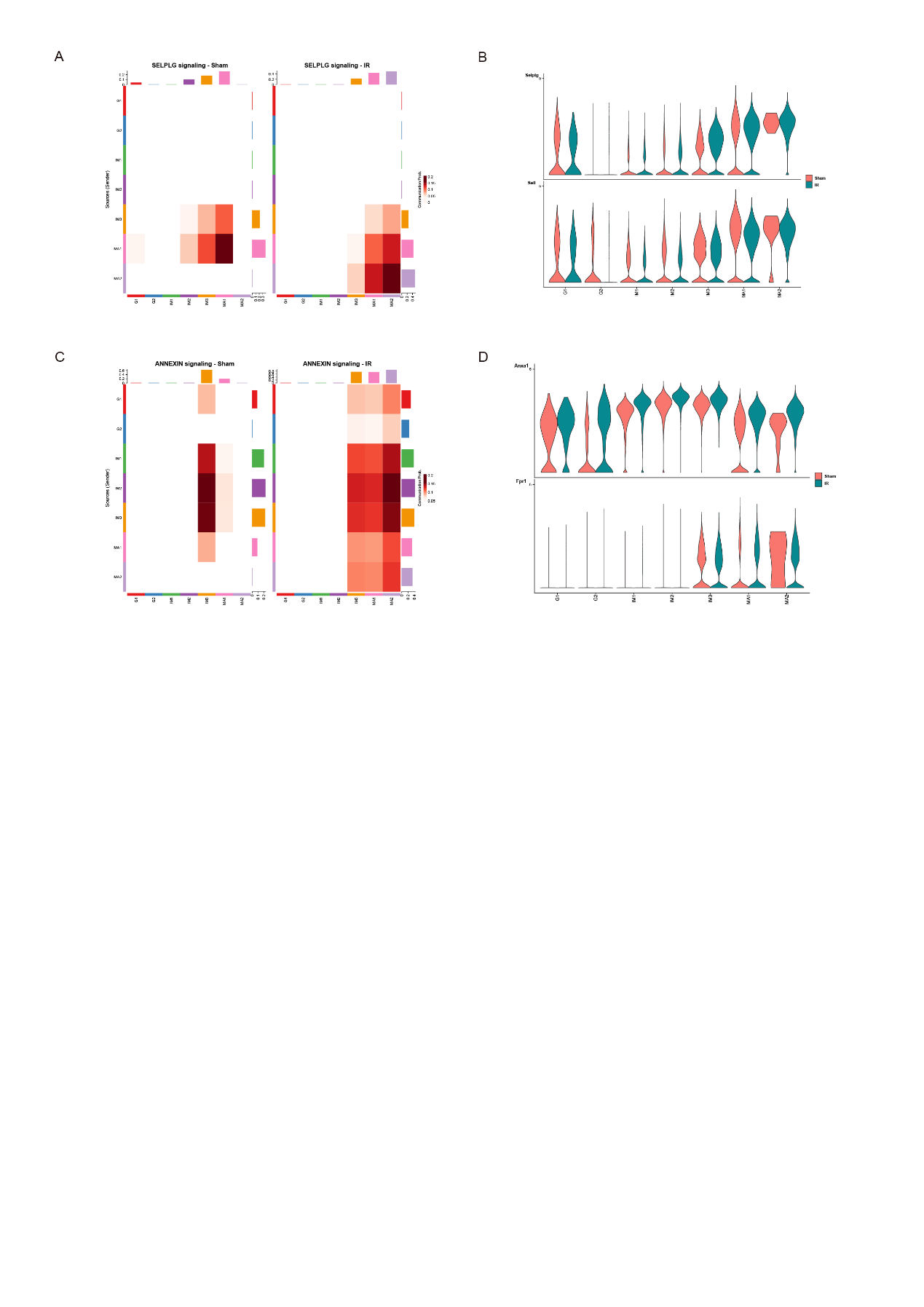


**Figure S8. Exclusion of SELPG and ANNEXIN pathways as markers for MA2 neutrophil subpopulations.** (A-D) Characterisation of the expression of the (A-B) SELPG and (C-D) ANNEXIN pathway and its major ligand receptors in the sham group and 80d post-radiation by CellChat analysis.


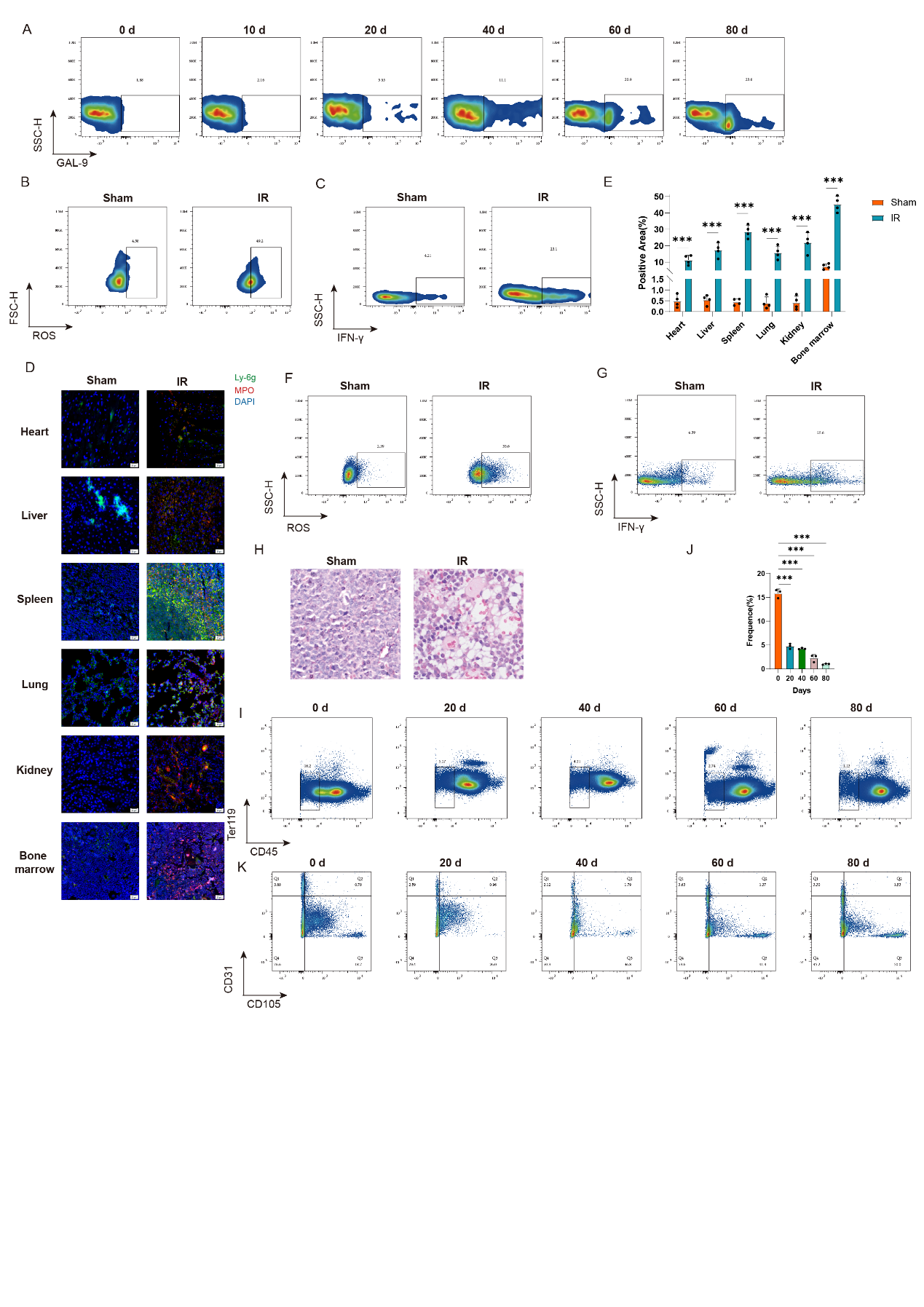


**Figure S9. GAL-9^high^ neutrophils are crucial mediators for inducing frailty after local radiation injury.** (A) Representative flow plots of circulating GAL-9^high^ neutrophils at different time points post-radiation. (B-C) Representative flow plots of (B) ROS and (C) IFN-γ expression of circulating neutrophils after treatment in the sham group and 80d post-radiation. (D-E) Representative plots and statistics of MPO expression of neutrophils in multiple organs by immunofluorescence. n=4. (F-G) Representative flow plots of bone marrow neutrophils (F) ROS and (G) IFN-γ expression after treatment in the sham group and 80d post-radiation. (H) Representative plots of HE staining of bone marrow in the sham group and 80d post-radiation. (I-K) Representative flow plots and frequence of bone marrow (I-J) non-immune cells (CD45^-^ Ter119^-^), (K) including endothelial cells, SECs, and stromal cells at different time points post-radiation. n=3. Data are presented as mean ± SD; each dot represents an individual animal from at least 2–3 independent experiments that used male and female mice. ***p < 0.001. Statistical analyses were performed using unpaired Student’s t test (E) and one-way ANOVA (J).


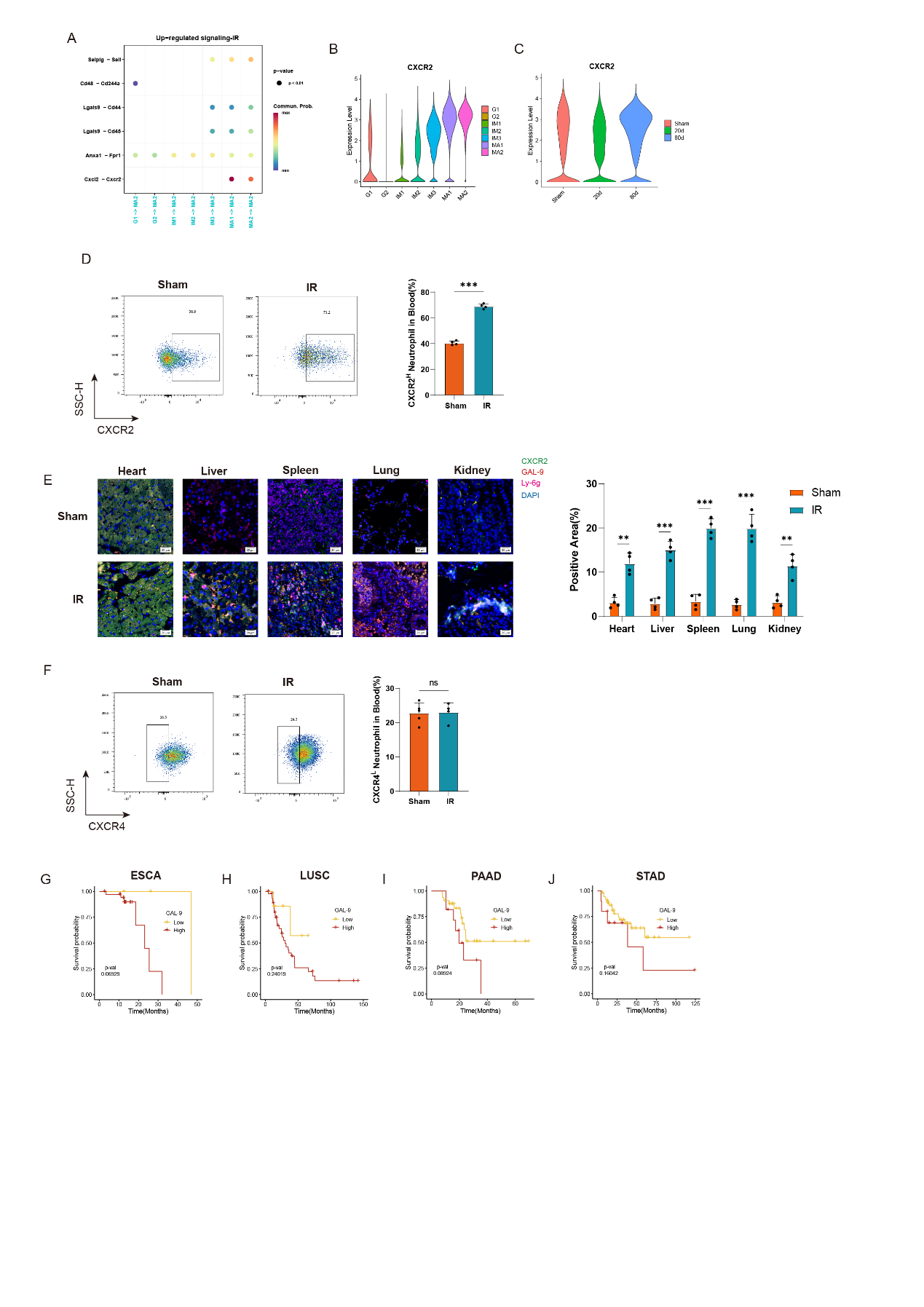


**Figure S10. Analysis of GAL-9^high^ neutrophil chemotaxis and TCGA database analysis.** (A) Analysis of the major up-regulated pathways from other neutrophil subpopulations to the MA2 neutrophil subpopulation after radiation by CellChat. (B) CXCR2 expression of each population of neutrophils by violin plots. (C) CXCR2 expression of each group of neutrophils by violin plots. (D) Representative flow plots and frequency of circulating CXCR2^high^ neutrophils in the sham group and 80d post-radiation. n=4. (E) Representative plots and statistics of CXCR2^+^ GAL-9^+^ neutrophils in multi-organs by immunofluorescence at 80d post-radiation and the sham group. n=4. (F) Representative flow plots and frequency of circulating CXCR4^low^ neutrophils in the sham group and 80d post-radiation. n=5. (G-J) K-M curve analysis of GAL-9^high^ neutrophils-related genes in (G) ESCA, (H) LUSC, (I) PAAD, and (J) STAD in the TCGA database. p-value was shown. Data are presented as mean ± SD; each dot represents an individual animal from at least 2–3 independent experiments that used male and female mice. ns, not significant, **p < 0.01,***p < 0.001. Statistical analyses were performed using unpaired Student’s t test (D-F) and the log-rank test (G-J).


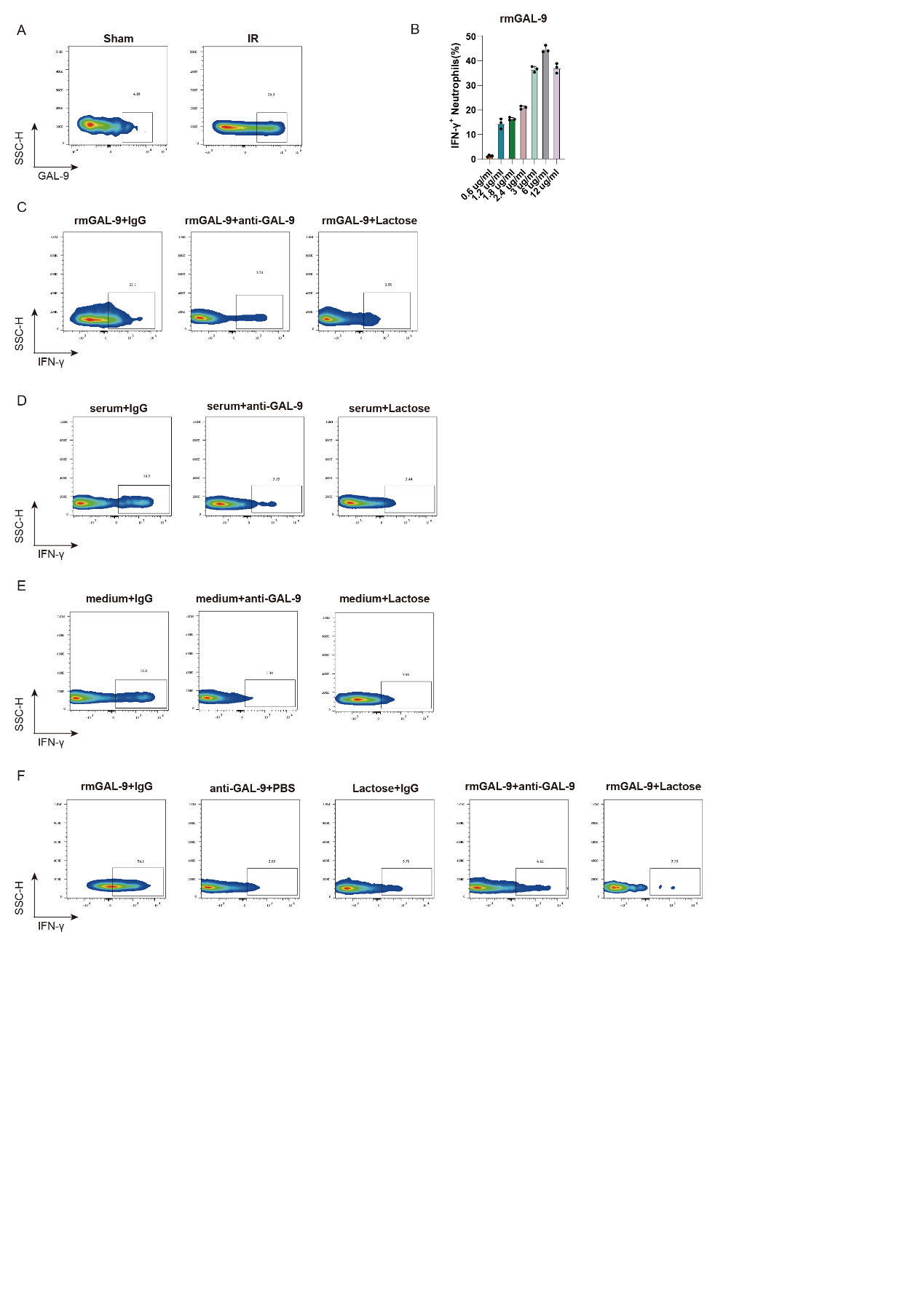


**Figure S11.** **GAL-9 protein induce neutrophils IFN-γ expression.** (A) Representative flow plots of intracellular GAL-9 protein in circulating neutrophils in the sham group and 80d post-radiation. (B) Dose-gradient experiments with exogenous supplementation of rmGAL-9 protein to induce neutrophil IFN-γ production. n=3. (C) Representative flow plots of IFN-γ expression of circulating neutrophils in the sham group after treatment with different conditions. (D-F) Representative flow plots of IFN-γ expression of circulating neutrophils in the sham group by treatment with (D) circulating serum at 80d post-radiation, (E) culture supernatant of GAL-9^high^ neutrophils, and (F) IFN-γ expression of GAL-9^high^ neutrophils 80d post-radiation under different stimuli. Data are presented as mean ± SD; each dot represents an individual animal from at least 2 independent experiments that used male and female mice.


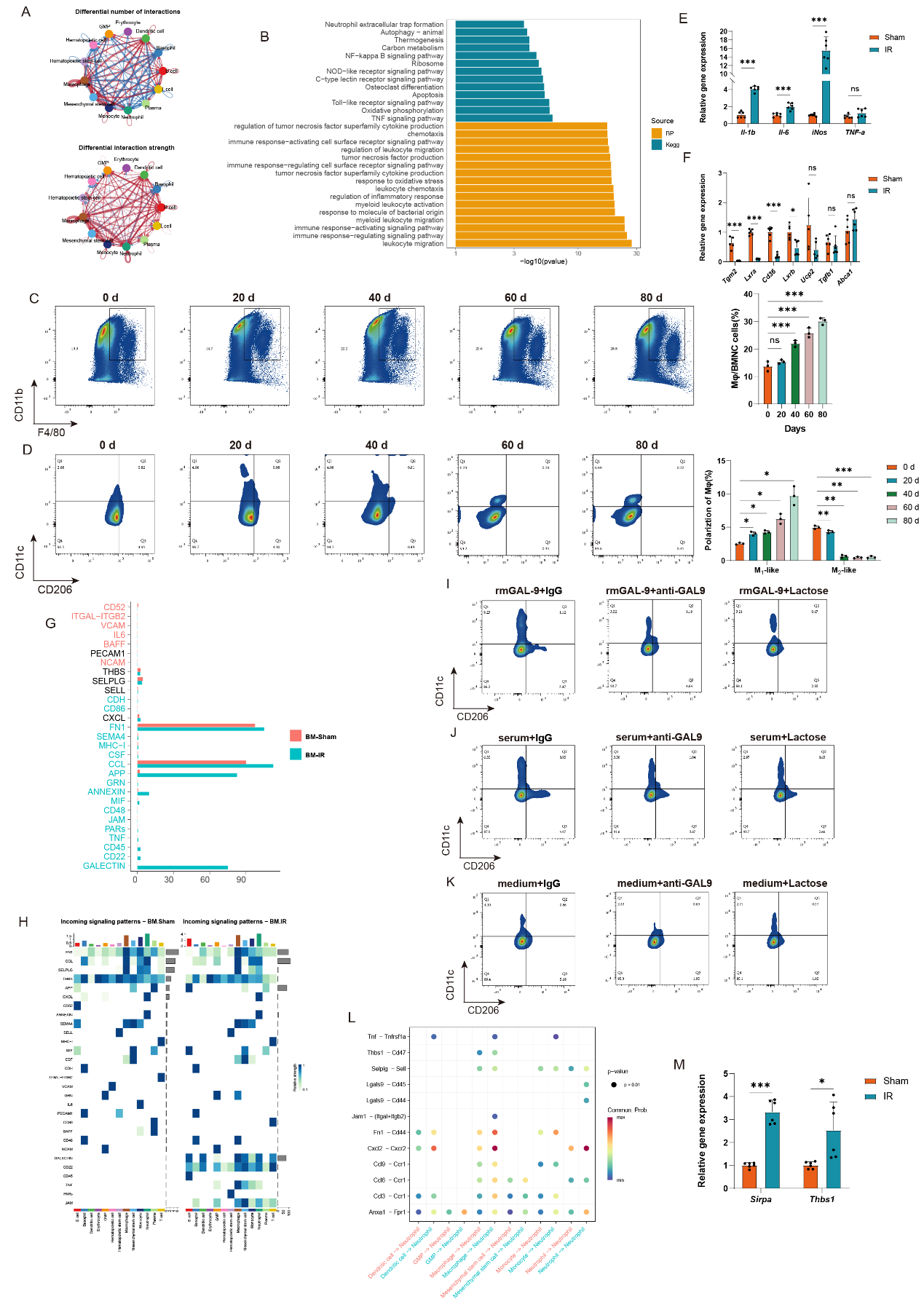


**Figure S12. GAL-9 protein induce bone marrow macrophages pro-inflammatory state.** (A) Differential number/strength of interactions with bone marrow cells in the local radiation group relative to the sham group by CellChat analysis (red means higher, blue means lower, and the width of the line represents the interaction strength). (B) 80d post-radiation of bone marrow macrophages relative to the sham group majorly upregulated BP and the KEGG pathway. (C-D) Representative flow plots and frequence of bone marrow (C) macrophages and (D) their polarization state(M_1_-like: CD11b^+^ F4/80^+^ CD11c^+^ CD206^-^, M_2_-like: CD11b^+^ F4/80^+^ CD11c^-^ CD206^+^) at different time points post-radiation. n=3. (E-F) (E) Inflammation-related genes and (F) pro-inflammatory resolution-related genes expression in bone marrow macrophages in the sham group and 80d post-radiation. n = 3. (G) The main bone marrow cell interaction pathways by CellChat analysis for the sham group and 80d post-radiation. H) The main Incoming signaling patterns of bone marrow cells in the sham group and 80d post-radiation by CellChat analysis. (I-K) Representative flow plots of the effect of (I) rmGAL-9 protein, (J) circulating serum at 80d post-radiation, and (K) culture supernatant of GAL-9^high^ neutrophils on the polarization of bone marrow macrophages. (L) Analysis of the major up- and down-regulated pathways of bone marrow cells to neutrophils by CellChat analysis. (M) Bone marrow macrophages related genes expression in the sham group and 80d post-radiation. n = 6. Data are presented as mean ± SD; each dot represents an individual animal from at least 2–3 independent experiments that used male and female mice. ns, not significant, *p < 0.05, **p < 0.01,***p < 0.001. Statistical analyses were performed using unpaired Student’s t test (E, F, M), one-way ANOVA (C), and two-way ANOVA (D).


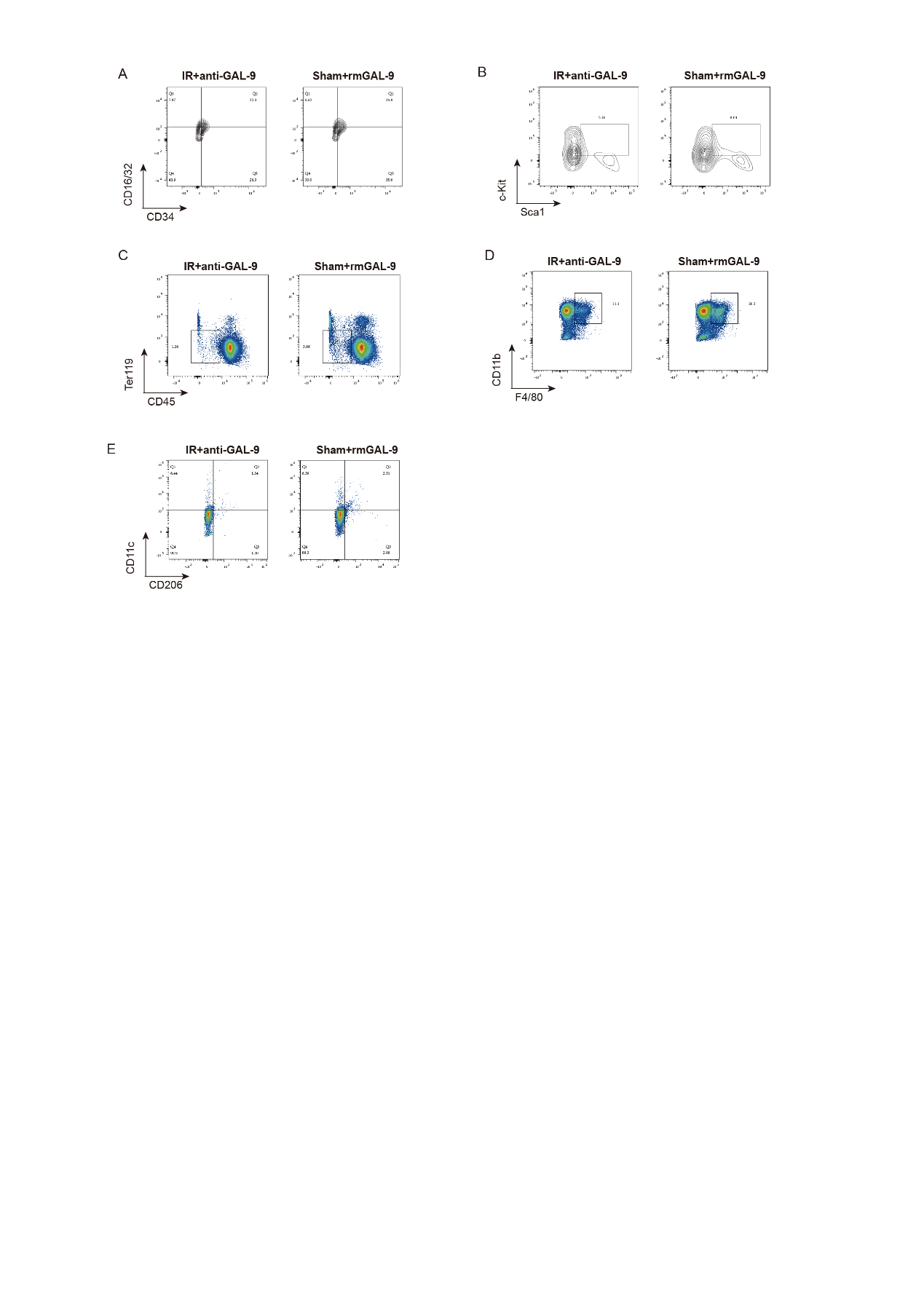


**Figure S13. GAL-9 protein intervention.** (A-E) Representative flow plots of bone marrow (A) CMP cells, (B) CLP cells, (C) non-immune cells, (D) macrophages, and (E) their polarization state after different treatments in local radiation and the sham group.


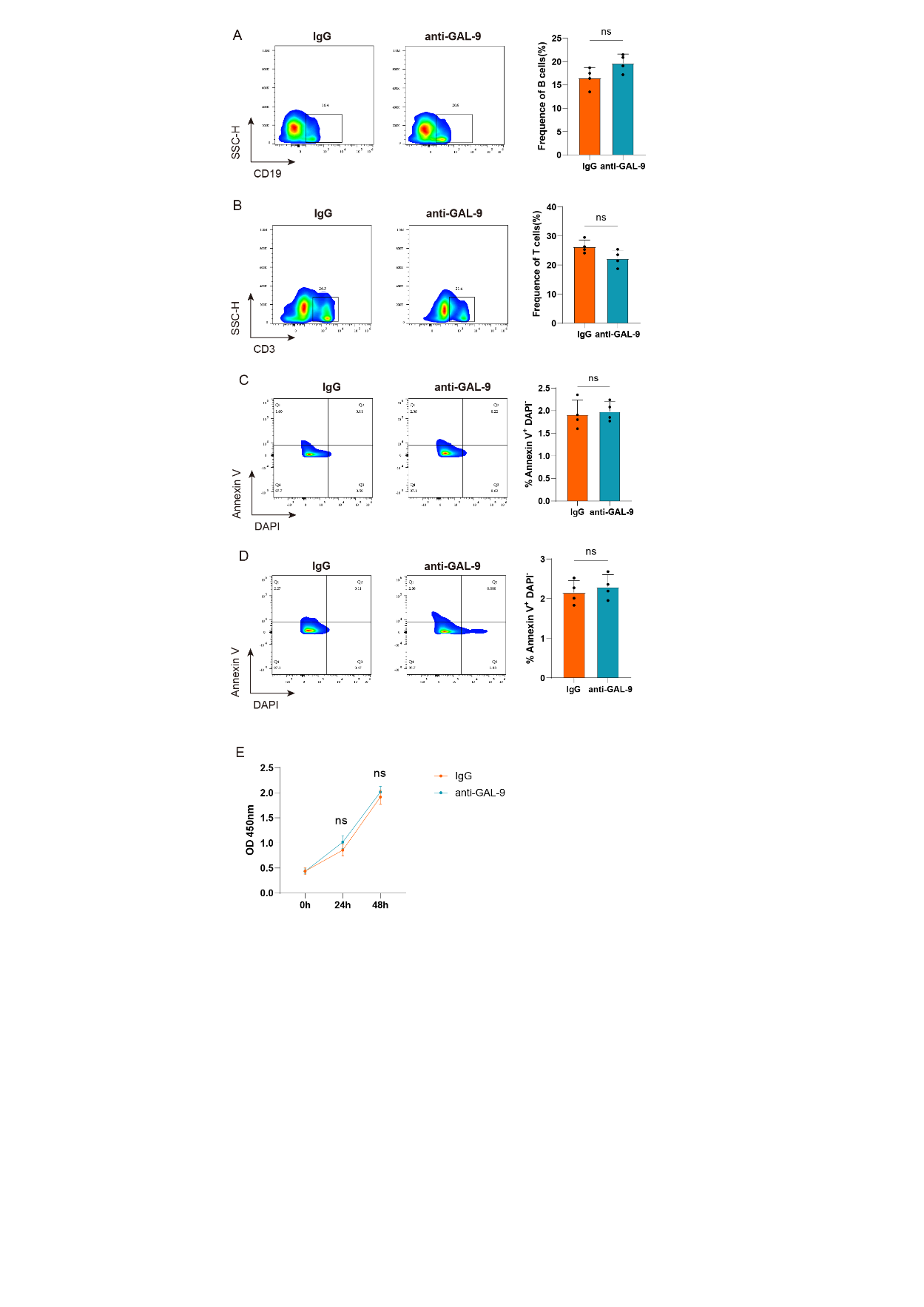


**Figure S14. Safety analysis of anti-GAL-9 antibody.** (A) Representative flow plots and frequency of circulating B cells in the IgG group and anti-GAL-9 antibody group. n=4. (B) Representative flow plots and frequency of circulating T cells in the IgG group and anti-GAL-9 antibody group. n=4. (C-D) Representative flow plots and frequency of apoptotic HUVEC cells in the IgG group and anti-GAL-9 antibody group after 24h and 48h. n=4. (E) The cell counts of HUVEC cells in the IgG group and anti-GAL-9 antibody group analysed by the CCK-8 kit. n=4. Data are presented as mean ± SD; each dot represents an individual animal from at least 2–3 independent experiments that used male and female mice. ns, not significant. Statistical analyses were performed using unpaired Student’s t test.


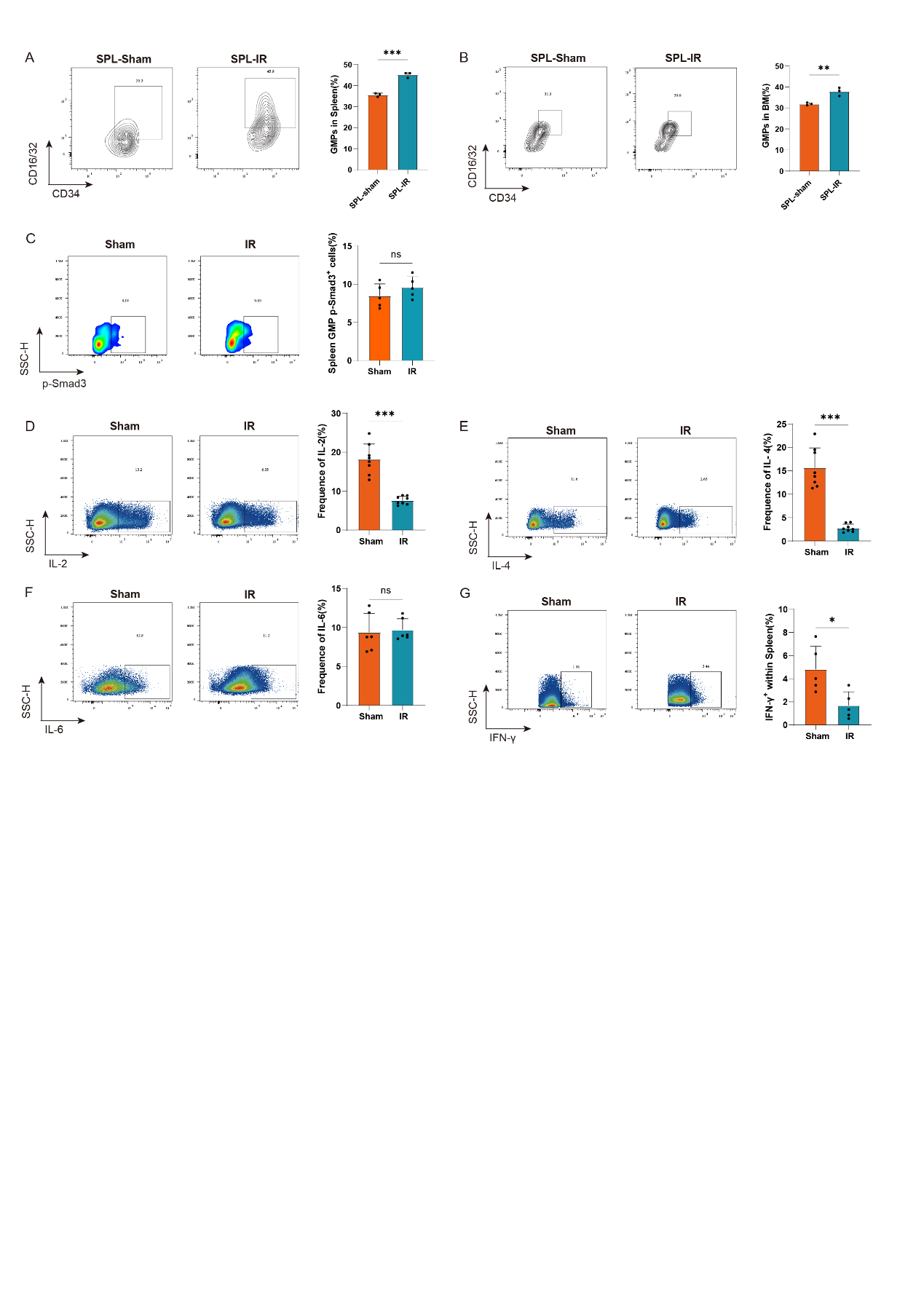


**Figure S15. Expression of common activation mediators of the splenic JAK1/2-STAT1 pathway in the sham group and 20 days post-radiation.** (A-B) Representative flow plots and frequence of (A) splenic and (B) bone marrow GMP cells in SPL-Sham or SPL-IR. n=3. (C)Representative flow plots and frequency of phosphorylation levels of Smad3 in splenic GMP cells from sham group and 20d post-radiation. n=5. (D-G) Representative flow plots and frequence of splenic IL-2, IL-4, IL-6, and IFN-γ expression in the sham group and 20d post-radiation. n=5-8. Data are presented as mean ± SD; each dot represents an individual animal from at least 2–3 independent experiments that used male and female mice. ns, not significant, *p < 0.05, **p < 0.01,***p < 0.001. Statistical analyses were performed using an unpaired Student’s t-test (A-G).


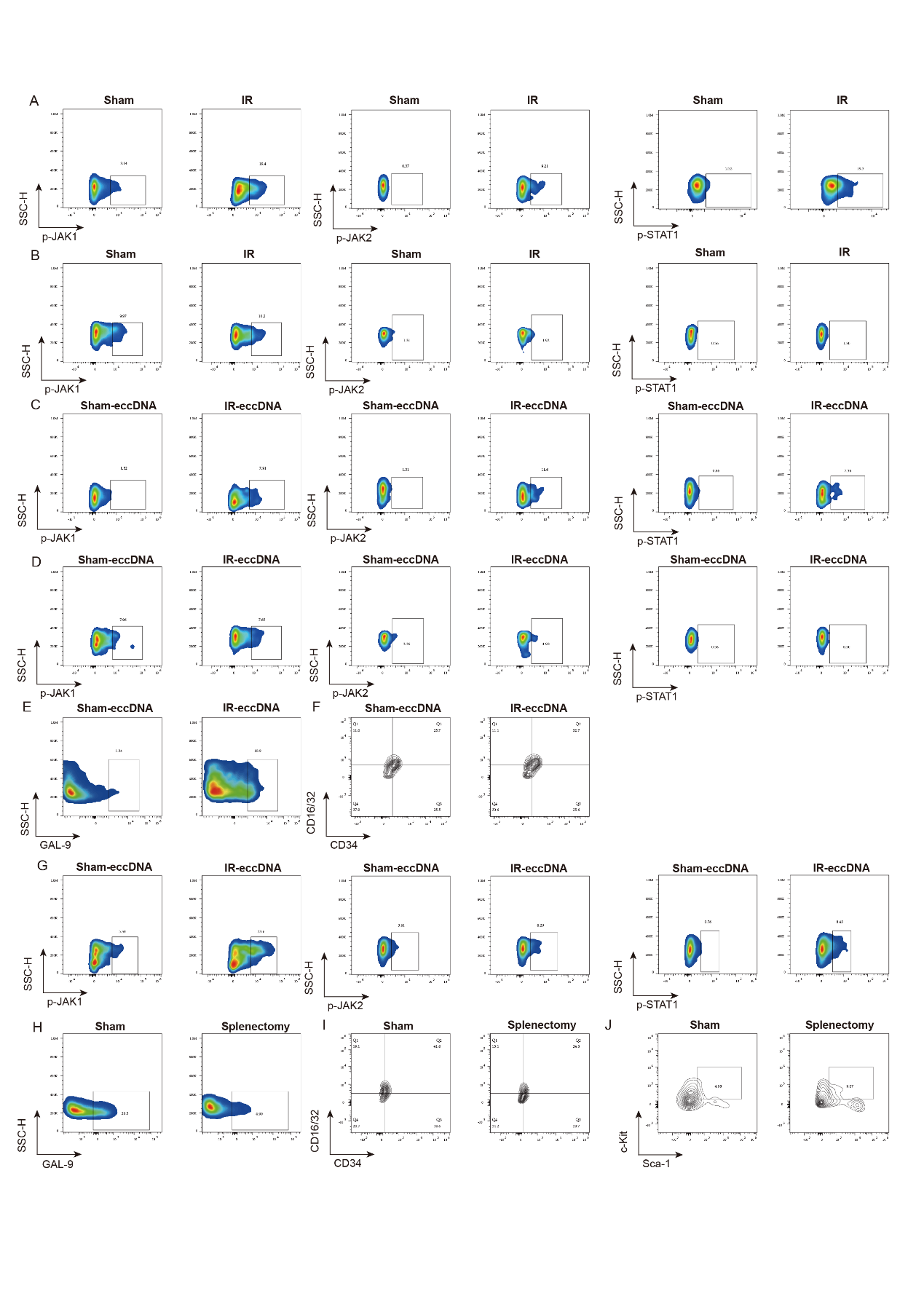


**Figure S16. Skin eccDNA triggers JAK1/2-STAT1 pathway in splenic GMP cells to produce GAL-9^high^ neutrophils.** (A-B) Representative flow plots of phosphorylation levels of JAK1, JAK2, and STAT1 in (A) splenic and (B) bone marrow GMP cells in the sham group and 20d post-radiation. (C-F) Representative flow plots of phosphorylation levels of JAK1, JAK2, and STAT1 in (C) splenic and (D) bone marrow GMP cells, (E) circulating GAL-9^high^ neutrophils, and (F) splenic GMP cells of recipients injected with Sham-eccDNA or IR-eccDNA. (G) Representative flow plots of phosphorylation levels of JAK1, JAK2, and STAT1 in splenic GMP cells under different treatment conditions. (H-J) Representative flow plots of (H) circulating GAL-9^high^ neutrophils, bone marrow (I) GMP, and (J) CLP cells after radiation in the splenectomy and sham group.
